# Silver nanoparticle-induced site-specific strand cleavage of chemically modified oligonucleotides for long-chain DNA assembly

**DOI:** 10.1093/nar/gkag525

**Published:** 2026-06-11

**Authors:** Masahito Inagaki, Mikiya Kase, Haruka Hiraoka, Natsuhisa Oka, Fumitaka Hashiya, Naoko Abe, Yasuaki Kimura, Hiroshi Abe

**Affiliations:** Department of Chemistry, Graduate School of Science, Nagoya University, Furo-cho, Chikusa-ku, Furo-cho, Chikusa-ku, Nagoya, Aichi 464-8602, Japan; Department of Chemistry, Graduate School of Science, Nagoya University, Furo-cho, Chikusa-ku, Furo-cho, Chikusa-ku, Nagoya, Aichi 464-8602, Japan; Department of Chemistry, Graduate School of Science, Nagoya University, Furo-cho, Chikusa-ku, Furo-cho, Chikusa-ku, Nagoya, Aichi 464-8602, Japan; Department of Chemistry and Biomolecular Science, Faculty of Engineering, Gifu University, 1-1 Yanagido, Gifu 501-1193, Japan; Institute for Glyco-core Research (iGCORE), Gifu University, 1-1 Yanagido, Gifu 501-1193, Japan; Center for One Medicine Innovative Translational Research (COMIT), Institute for Advanced Study, Gifu University, 1-1 Yanagido, Gifu 501-1193, Japan; Research Center for Materials Science, Nagoya University, Furo-cho, Chikusa-ku, Nagoya, Aichi 464-8602, Japan; CREST, Japan Science and Technology Agency, Gobancho, Chiyoda-ku, Tokyo 102-0076, Japan; Department of Chemistry, Graduate School of Science, Nagoya University, Furo-cho, Chikusa-ku, Furo-cho, Chikusa-ku, Nagoya, Aichi 464-8602, Japan; Department of Chemistry, Graduate School of Science, Nagoya University, Furo-cho, Chikusa-ku, Furo-cho, Chikusa-ku, Nagoya, Aichi 464-8602, Japan; Department of Chemistry, Graduate School of Science, Nagoya University, Furo-cho, Chikusa-ku, Furo-cho, Chikusa-ku, Nagoya, Aichi 464-8602, Japan; CREST, Japan Science and Technology Agency, Gobancho, Chiyoda-ku, Tokyo 102-0076, Japan; Institute for Glyco-core Research (iGCORE), Nagoya University, Furo-cho, Chikusa-ku, Nagoya, Aichi 464-8601, Japan

## Abstract

Here, we report a novel site-specific oligonucleotide strand cleavage reaction mediated by silver nanoparticles (AgNPs) that is applicable for synthesizing sticky ends of PCR (polymerase chain reaction)-amplified DNA for DNA ligase-mediated ligation to build up long-chain DNA. For this purpose, we designed and synthesized modified DNA as a PCR primer bearing 3′-phosphorothiolate linkage at strand cleavage sites. The AgNP surface acts as a Lewis acid to activate the 3′-phosphorothiolate linkage of DNA and induces hydrolysis of the 3′-phosphorothiolate linkage to produce 3′-thiol-DNA and 5′-phosphorylated DNA fragments. In this study, we discuss the factors that induce oligonucleotide strand cleavage by AgNPs, such as nanoparticle size, reaction time, reaction temperature, and chemical modification of nanoparticles. Polyethylene glycol modification of AgNPs was found to maximize oligonucleotide strand cleavage activity. We demonstrated the synthesis of 848-bp DNA by DNA ligase-mediated ligation of 298- and 558-bp DNA fragments with sticky ends and eight bases overhang prepared using the AgNP-induced strand cleavage method. In addition, we successfully constructed a GFP-coding DNA and demonstrated the expression of GFP in HeLa cells. This research opens the possibility of developing new applications of metal nanoparticle chemistry for biotechnological tools.

## Graphical Abstract

**Graphical Abstract:**
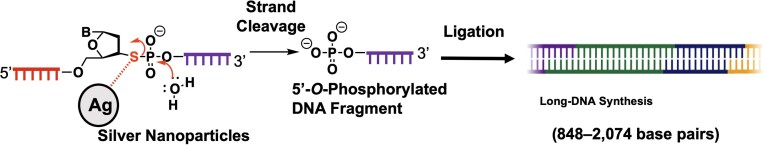

## Introduction

The development of a site-specific oligonucleotide strand cleavage method is urgently needed as a biotechnology research tool [1]. In particular, DNA (deoxyribonucleic acid) editing technology is an attractive topic for controlling and rewriting genetic information for diverse applications such as the generation of animal models for drug discovery, curing genetic diseases, and agricultural breeding [2]. The development of genome-scale long-chain DNA assembly methods, such as Golden Gate assembly [3] and Gibson assembly methods [4], has stimulated genome-editing research [5, 6]. These techniques are based on the enzymatic digestion of DNA, which has precisely controlled sequence specificity; in contrast, ligation efficiencies depend on the activity of enzymes [7, 8]. Several research groups have reported the use of the chemical oligonucleotide strand cleavage method for long-chain DNA assembly. Ikeda *et al*. reported a depyrimidination-induced DNA strand cleavage system by introducing 5-ethynyluridine at strand cleavage sites [9]. This strand cleavage method is site-specific, and they demonstrated to prepare plasmid DNA by applying this strand cleavage method. However, strong basic conditions in aqueous methylamine at 70°C are required for strand cleavage, which is one of the drawbacks preventing its application to long-chain DNA assembly methods. Kellner *et al*. reported oxidative cleavage of the phosphorothioate linkage. This strand cleavage reaction is relatively mild and site-specific; however, desulfurizing side-reactions proceed, and two types of cleavage products with different phosphate attachment strands are generated [10]. As another approach, Hölz *et al*. reported a uracil DNA glycosylase-mediated strand cleavage system [11], which has been applied to prepare pNEB20A plasmid construction and cloning using uracil excision by the Bitinaite group [12]. This strand cleavage method is accurate and can be applied for DNA ligation of cleaved fragments with >70% yield. However, the nucleobase at the cleavage site is restricted to only the A-T(U) base pair.

**Figure 1.**
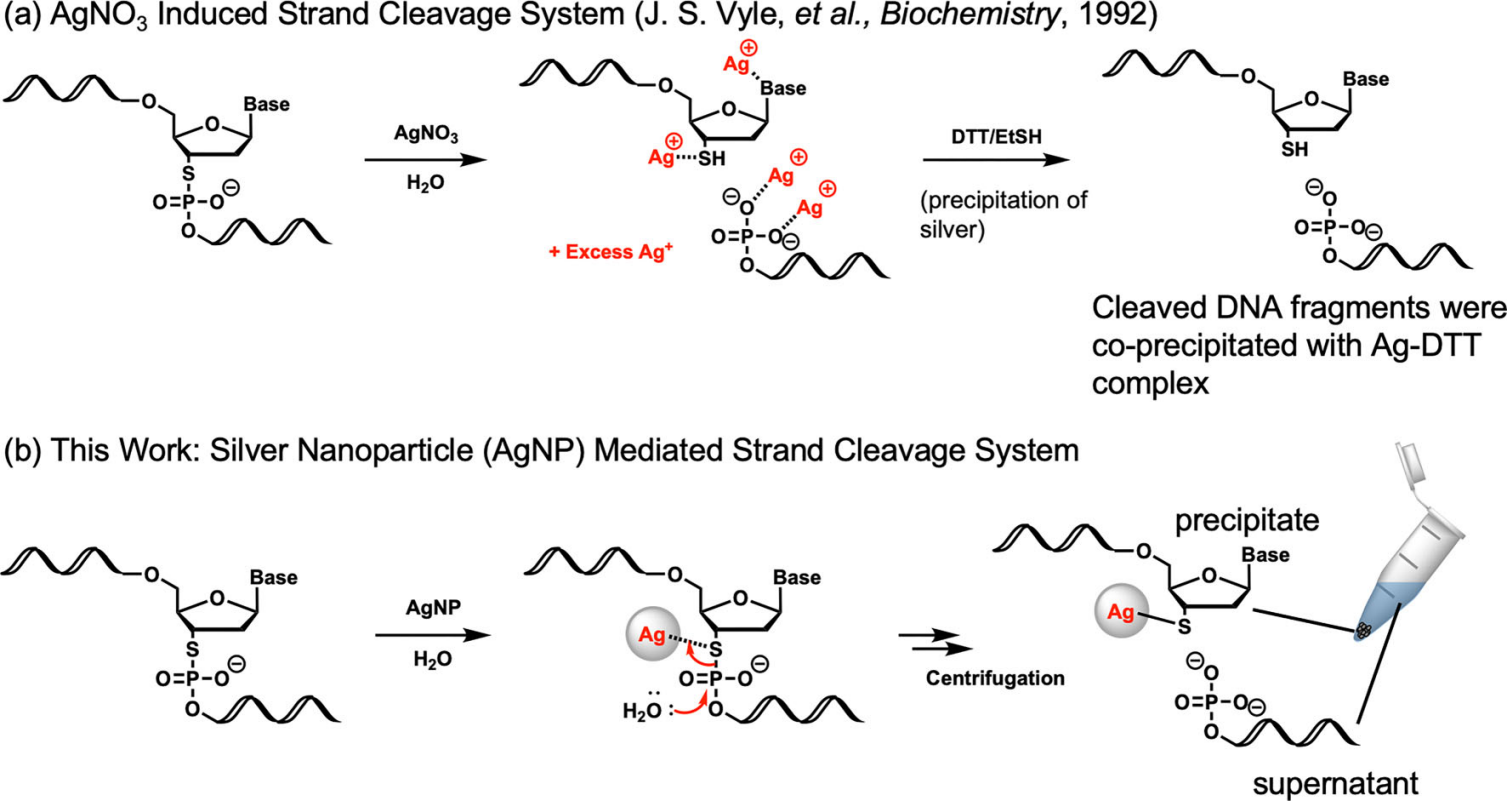
3′-Phosphorothiolate oligonucleotide cleavage reaction. (**a**) State of the art AgNO_3_ induced cleavage [13, 14]. (**b**) AgNP-mediated strand cleavage reaction developed in this work.

In 1990–1992, Cosstick [13] and Vyle *et al*. [14] reported silver nitrate-induced cleavage of 3′-phosphorothiolate linkage DNA. The cleavage reaction is completed within a few minutes at ambient temperature, which is a moderate cleavage condition and is expected to be applicable for long-chain DNA assembly methods (Fig. 1a) [15]. Furthermore, since this chain-cleavage reaction occurs specifically at the 3′-phosphorothiolate linkage, there is no limitation on the types of nucleobases, and it is a great benefit that the cleavage sequence can be freely designed. According to their report, we investigated strand cleavage of DNA with 3′-phosphorothiolate linkage by silver nitrate. As a result, the strand cleavage proceeded almost completely within 5 min, confirming that the desired cleavage product was generated. However, as will be described later, when thiols, such as dithiothreitol (DTT), were added to remove silver ions [16], the DNA co-precipitated with the precipitation of the DTT–Ag complex, and the recovery amount of the cleavage product DNA decreased [17]. The same problem was observed when silver nitrate was used for deprotection of the trityl group on the 5′-thiol function of the oligonucleotides [18, 19]. Therefore, cleavage of the 3′-phosphorothiolate linkage by silver ion treatment is difficult to apply as a practical methodology. We wondered whether this problem could be solved using silver nanoparticles (AgNPs). That is, by treating DNA with 3′-phosphorothiolate linkage with AgNPs of appropriate size, Lewis acidically activates the 3′-phosphorothiolate linkage on the surface of AgNPs, and hydrolysis of the 3′-phosphorothiolate bond is expected to be induced. As a result, the 3′-thiol DNA fragment is trapped on the surface of the AgNPs, while the 5′-phosphorylated DNA remains in the aqueous solution. In addition, AgNPs can be easily removed by centrifugation, and the desired cleavage product DNA can be obtained by collecting the supernatant after centrifugation (Fig. 1b).

In this study, to develop a DNA strand cleavage system utilizing AgNPs, we investigated in detail multiple factors for DNA strand cleavage, such as particle size, reaction temperature, reaction time, and surface modification of nanoparticles. To date, no reports have been published on oligonucleotide strand cleavage reactions using metal nanoparticles, and the results of this study are innovative in terms of discovering new functions of metal nanoparticles and developing new application fields. Using optimized nanoparticle-induced oligonucleotide strand cleavage conditions, we succeeded in preparing the sticky end by strand cleavage of the amplification products by polymerase chain reaction (PCR) and demonstrated the construction of long-chain DNA by enzymatic ligation of chemically produced sticky-end DNA fragments.

## Materials and methods

### Chemical synthesis

Synthesis protocol and characterization can be found in the Supplementary material.

### Strand cleavage by silver nitrate

One microliter of 30 µM 3′*S*-modified DNA 20-mer (3′*S*-DNA1: 5′-TAA**Ts**CACATTAATTGCGTT-FAM-3′) was mixed with 50 mM silver nitrate aqueous solution (29 µl) and incubated at room temperature for 5–40 min. When the specified time was reached, 5 µl of each reaction solution was taken, and the reaction was quenched by adding 60 mM aqueous ethanethiol (EtSH) solution (5 µl). At this time, the formation of a white precipitate derived from the DNA–silver ion complex was observed. 2× loading buffer (10 µl) was added to this mixture (10 µl). After heat treatment at 95°C for 5 min, the samples were analyzed by 15% denaturing polyacrylamide gel electrophoresis (dPAGE) (containing 7.5 M urea, 10 cm × 12 cm, 30 mA, 20 min, and 6 µl loading). A gel electrophoresis image was obtained using a gel image analyzer (BioRad) using FAM-derived fluorescence detection.

### Investigation of the size dependency of nanoparticles in the DNA strand cleavage

Ten microliters of 3 µM 3′*S*-modified DNA (3′*S*-DNA1: 5′-TAAC**Ts**CACATTAATTGCGTT-FAM-3′) was added to 50 µl AgNP dispersion (10 nm, 0.02 mg/ml) and the mixture was incubated at 37°C for 31 h. Twenty microliters of the reaction solution was taken, and 20 µl of 2× loading buffer was added to the solution. After heat treatment at 95°C for 5 min, it was analyzed by 15% dPAGE (containing 7.5 M urea, 10 cm × 12 cm, 30 mA, 20 min, and 6 µl loading). A gel electrophoresis image was obtained by gel image analyzer (BioRad) from FAM-derived fluorescence.

### Investigation of reaction time/temperature dependency for the DNA strand cleavage by silver nanoparticle treatment

Ten microliters of 3 µM 3′*S*-modified DNA (3′*S*-DNA1: 5′-TAAC**Ts**CACATTAATTGCGTT-FAM-3′) was added to 50 µl AgNP dispersion (10 nm, 0.02 mg/ml) and incubated at 70°C or 95°C. Thirty microliters of the reaction solution was taken, and 30 µl of 2× loading buffer was added to the reaction mixture. After heat treatment at 95°C for 5 min, it was analyzed by 15% dPAGE (containing 7.5 M urea, 10 cm × 12 cm, 30 mA, 20 min, and 6 µl loading). A gel electrophoresis image was obtained by gel image analyzer (BioRad) from FAM-derived fluorescence.

### DNA strand cleavage by PEGylated silver nanoparticle

Nine microliters of 3.4 µM 3′-*S*-modified DNA (3′*S*-DNA1: 5′-TAAC**Ts**CACATTAATTGCGTT-FAM-3′) was added to 51 µl of surface PEG-modified AgNP dispersion (10 nm), and the mixture was incubated at the specified time and temperature. The polyethylene glycol (PEG)-modified AgNP dispersion was prepared by adding 1 µl aqueous solution of PEG with an average molecular weight of 5000, having terminal thiols (23.9 g/l), *O*-[2-(3-mercaptopropionylamino)ethyl]-*O*’-methylpolyethylene glycol, to 50 µl of a commercially available AgNP dispersion (Sigma-Aldrich, 10 nm, 0.02 mg/ml). Twenty microliters of the reaction solution was taken and mixed with 20 µl of 2× loading buffer. After heat treatment at 95°C for 5 min, it was analyzed by 15% dPAGE (containing 7.5 M urea, 10 cm × 12 cm, 30 mA, 20 min, and 6 µl loading). A gel electrophoresis image was obtained by gel image analyzer (BioRad) from FAM-derived fluorescence.

### DNA preparation of 3′-overhang sticky end by strand cleavage of double-stranded DNA

Three micromolar each of 3′*S*-modified DNA (3′*S*-DNA1: 5′-TAAC**Ts**CACATTAATTGCGTT-FAM-3′) and complementary DNA (5′-AACGCAATTAATGTGAGTTA-3′) were mixed and treated at 95°C for 3 min. After that, the mixture was placed on an ice bath, and the annealing was performed by cooling for 10 min or more. After annealing, 9 µl of the solution was taken and added to 51 µl of surface PEG-modified AgNP dispersion (10 nm). The mixture was incubated at 50°C for the specified time. Thirty microliters of the reaction solution was collected and mixed with 30 µl of 2× loading buffer. After heat treatment at 95°C for 5 min, it was analyzed by 15% dPAGE (containing 7.5 M urea, 10 cm × 12 cm, 30 mA, 20 min, and 6 µl loading). A gel electrophoresis image was obtained by gel image analyzer (BioRad) from FAM-derived fluorescence.

### Demonstration of precipitation of silver nanoparticles by centrifugation

Seven-hundred microliters of a dispersion of AgNPs with particle sizes of 10, 20, and 100 nm (Sigma-Aldrich, 0.02 mg/ml sodium citrate aqueous solution) was diluted with ultra-deionized water (700 µl), and then absorption spectra were measured using a quartz cell (optical path length: 1 cm, optical path width: 1 cm). After measuring the absorption spectrum, the AgNP dispersion diluted with ultra-deionized water (700 µl) was transferred to an Eppendorf tube, centrifuged at 15 000 rpm for 1 h to precipitate the nanoparticles, and the supernatant was collected and transferred to the quartz cell again. After transferring to the cell (optical path length: 1 cm, optical path width: 1 cm), the absorption spectrum was measured.

### Evaluation of the dispersion property of PEGylated AgNP

The absorption spectra of (i) AgNP dispersion without surface modification, (ii) AgNP dispersion without surface modification in the presence of buffer/salts, (iii) PEGylated AgNP dispersion, and (iv) PEGylated AgNP dispersion in the presence of buffer/salts were measured by using a quartz cell (light path length: 1 cm, light path width: 1 cm). The solution preparation was conducted as follows:

1. AgNP dispersion without surface modification: 350 µl of a dispersion of AgNPs with a particle size of 20 nm (Sigma-Aldrich, 0.02 mg/ml sodium citrate aqueous solution) was diluted with ultra-deionized water (1050 µl).
2. AgNP dispersion without surface modification in the presence of buffer/salts: 350 µl of a dispersion of AgNPs with a particle size of 20 nm (Sigma-Aldrich, 0.02 mg/ml sodium citrate aqueous solution) was diluted with ultra-deionized water (630 µl) and added 100 mM Tris–HCl buffer (pH 8.3; 140 µl), 500 mM potassium chloride aqueous solution (140 µl), and 15 mM magnesium chloride aqueous solution (140 µl).
3. PEGylated AgNP dispersion: 350 µl of a dispersion of AgNPs with a particle size of 20 nm (Sigma-Aldrich, 0.02 mg/ml sodium citrate aqueous solution) and 140 µl of 23.9 g/l aqueous solution of *O*-[2-(3-mercaptopropionylamino)ethyl]-*O*'-methylpolyethylene glycol (average molecular weight 5 000, Sigma-Aldrich) were mixed and diluted with ultra-deionized water (910 µl).
4. PEGylated AgNP dispersion in the presence of buffer/salts: 350 µl of a dispersion of AgNPs with a particle size of 20 nm (Sigma-Aldrich, 0.02 mg/ml sodium citrate aqueous solution) and 140 µl of 23.9 g/l aqueous solution of *O*-[2-(3-mercaptopropionylamino)ethyl]-*O*'-methylpolyethylene glycol (average molecular weight 5 000, Sigma-Aldrich) were mixed and diluted with ultra-deionized water (490 µl). To the mixture, 100 mM Tris–HCl buffer (pH 8.3; 140 µl), 500 mM potassium chloride aqueous solution (140 µl), and 15 mM magnesium chloride aqueous solution (140 µl) were added.

### Comparison of the recovery yield of the DNA strand cleavage reaction between silver nitrate and silver nanoparticle

#### Silver nitrate treatment

Two microliters of 122 µM 3′*S*-modified DNA 20-mer (3′*S*-DNA1: 5′-TAA**Ts**CACATTAATTGCGTT-FAM-3′) was mixed with 50 mM silver nitrate aqueous solution (38 µl) and incubated at room temperature for 1.5 h. When the predetermined time was reached, the reaction was stopped by adding 60 mM DTT aqueous solution (40 µl) to the reaction solution (40 µl). At this time, the formation of a white precipitate derived from DTT–silver ion complex was observed. This mixture (80 µl) was diluted with ultra-deionized water (20 µl), mixed well, and the precipitate was removed by centrifugation at 15 000 rpm for 1 h. The supernatant was collected and concentrated using an ultrafiltration centrifugal filter (Amicon Ultra 3K, manufactured by Merck) according to the manufacturer’s recommended protocol. The recovered amount of the product was calculated by quantifying the cleavage product by measuring the absorbance of the concentrated sample solution at 260 nm derived from the nucleotides using NanoDrop. The concentrated sample solution was diluted with ultra-deionized water to prepare a 0.50 µM aqueous solution. Five microliters of 0.50 µM cleavage product solution was mixed with 2× loading buffer (5 µl). After treatment at 95°C for 5 min, the samples were analyzed by 15% dPAGE (containing 7.5 M urea, 10 cm × 12 cm, 30 mA, 20 min, and 5 µl application). A gel electrophoresis image was obtained by gel image analyzer (BioRad) from FAM-derived fluorescence.

#### AgNP treatment

Two microliters of 122 µM 3′*S*-modified DNA 20-mer (3′*S*-DNA1: 5′-TAA**Ts**CACATTAATTGCGTT-FAM-3′) was added to an AgNP dispersion with a particle size of 100 nm (Sigma-Aldrich, 0.02 mg/ml sodium citrate aqueous solution, 98 µl). The mixture was incubated at 90°C for 25 h. The AgNP was removed as a precipitate by centrifuging the reaction solution at 15 000 rpm for 1 h. The supernatant was collected and concentrated using an ultrafiltration centrifugal filter (Amicon Ultra 3K, manufactured by Merck) according to the manufacturer’s recommended protocol. The recovered amount of the product was calculated by quantifying the cleavage product by measuring the absorbance of the concentrated sample solution at 260 nm derived from the nucleotides using NanoDrop. The concentrated sample solution was diluted with ultra-deionized water to prepare a 0.50 µM aqueous solution. Five microliters of 0.50 µM cleavage product solution was mixed with 2× loading buffer (5 µl). After treatment at 95°C for 5 min, the sample was analyzed by 15% dPAGE (containing 7.5 M urea, 10 cm × 12 cm, 30 mA, 20 min, and 5 µl application). Gel images were obtained by a gel image analyzer (BioRad) from FAM-derived fluorescence.

### Application of modified DNAs for PCR

#### PCR using 3′*S*-DNA as template

One hundred and ten micromolars of 3′*S*-DNA template (3′*S*-DNA2: 0.37 µl) and 15 µM primer DNA: 5′-FAM-AACGCAATTAATGTGAGTTAGC-3′ (1.34 µl) were mixed and added to the mixture of dNTPs, various polymerases (KOD-Plus-Neo, PrimeSTAR HS, Phusion High Fidelity, Q5 High Fidelity, Deep Vent, Taq DNA polymerase), and polymerase buffers to make a total volume of 20 µl. Denaturation (95°C, 1 min), annealing (50°C, 30 s), and chain extension (72°C, 30 min) were performed, and the reaction solution was diluted with 2× loading buffer (20 µl). It was analyzed by 20% dPAGE (containing 7.5 M urea, 20 cm × 22 cm, 20 W, and 2 h). The gel was stained using 1× SYBR Green II solution, and the gel image was obtained using a gel image analyzer (BioRad).

#### PCR using 3′*S*-DNA primers

Twenty micromolars of 3′*S*-Forward primer (1.25 µl), 20 µM 3′*S*-Reverse primer (1.25 µl), 10 ng/µl template DNA (2.5 µl), 2 mM dNTP mixture (5 µl), 25 mM magnesium sulfate (3 µl), 10x PCR Buffer for KOD-Plus-Neo (5 µl), and ultra-deionized water (31 µl), were mixed and added to 1 U/µl KOD-Plus-Neo (1 µl). By using a thermal cycler (BioRad), the sample was heated to 95°C for 2 min. Subsequently, DNA was amplified by performing 30 cycles of denaturation (95°C, 15 s), annealing (55°C, 15 s), and chain extension (68°C, 30 s). The PCR product was purified using a Wizard column (Promega) according to the manufacturer’s recommended protocol to obtain amplified 3′*S*-modified DNA.

### Preparation of sticky ends by DNA strand cleavage reaction and application for DNA assembly

#### Preparation of sticky-end DNA fragments by silver nanoparticles

4.5 µl of 7.5 nM PCR product 3′*S*-modified DNA was added to PEGylated AgNP dispersion, which was prepared by mixing 10 nm AgNPs dispersion (Sigma-Aldrich, 200 µg/ml in sodium citrate buffer, 25 µl) and *O*-[2-(3-mercaptopropionylamino)ethyl]-*O*'-methylpolyethylene glycol (Sigma-Aldrich, average molecular weight 5 000, 23.9 g/l aqueous solution, 0.5 µl). The mixture was treated at 50°C for 2–4 h to cleave 3′-phosphorothiolate bonds and prepare sticky ends.

#### DNA ligase-mediated ligation of DNA fragments

7.5 nM AgNP-treated product DNA_F1 (3.6 µl), 7.5 nM AgNP-treated product DNA_F2 (3.6 µl), 10× T4 DNA Ligase Reaction Buffer (New England BioLabs, 0.90 µl), and ultra-deionized water (0.45 µl) were mixed. To this mixture, T4 DNA ligase (New England BioLabs, 2000 U/µl, 0.45 µl) was added and incubated at 25°C for 3 h. One microliter of 10× loading buffer was added to the reaction solution (9 µl) and analyzed by 1% agarose gel electrophoresis (running buffer: 1× TBE, 100 V, 30 min). The gel was stained using a 10 000× SYBR green I solution by shaking for 30 min, and a gel image was obtained using a gel image analyzer (manufactured by BioRad).

### GFP coding DNA constructure

DNA fragments encoding the CMV promoter or the GFP coding sequence were amplified by PCR using synthesized 3′*S*-modified primers with the pcDNA6.2 emGFP plasmid as template. Primer pairs producing PCR products with 10- or 18-nt overhang sticky ends were listed in Table 3. PCR reaction mixture contains 0.3 µM primers, 1 ng/µl template plasmid, 0.2 mM dNTPs, 1.5 mM MgSO_4_, 1× reaction buffer, and 0.02 U/µl KOD-Plus-Neo polymerase. Product lengths of the CMV-promoter fragment and GFP-coding fragment are 830 and 1 260 bp, respectively.

The obtained PCR products with these primers were purified by Wizard^®^ SV Gel and PCR Clean-Up system (Promega) according to the manufacturer’s protocol and treated with PEGylated AgNPs (10 nm) at 50°C for 4 h. Thereafter, the reaction mixture was diluted 10 times and heated at 90°C for 5 min to promote the dissociation of cleaved fragments from PCR products. After concentration by lyophilization, mixed solutions of CMV promoter and GFP-coding fragments (around 400 fmol each) were heated at 90°C for 3 min and cooled slowly to room temperature for annealing. Then, 50 U/µl of NEB T4 ligase was added to the mixture and incubated at 25°C for 3 h. As a control with short overhang sticky ends, DNA fragments with four bases overhang were prepared by BsaI treatment against roughly purified PCR products prepared as described above (see primer sequence in Table 3). For digestion, the BsaI reaction mixture containing 200–300 ng/µl PCR product, 1× CutSmart buffer, and 0.6 U/µl BsaI-HFv2 (NEB) was incubated at 37°C for 1 h. After incubation, digested fragments were purified by Wizard® SV Gel and PCR Clean-Up system (Promega), followed by ligation; the ligation protocol is the same as AgNP-treated fragments. To see whether AgNPs affect ligation efficiency or not, BsaI-treated DNA fragments were ligated in the presence or absence of AgNPs for comparison.

### Transfection of the ligated sample to HeLa cells

To confirm the function of protein synthesized from ligated products, these ligated solutions were transfected into HeLa cells after inactivation of T4 ligase by heat treatment at 65°C for 10 min. HeLa cells (1.0 × 10^4^) suspended in Dulbecco’s modified Eagle’s medium (DMEM) containing Fetal Bovine Serum (FBS) were seeded on a 96-well plate and incubated at 37°C under 5% CO_2_ atmosphere for 24 h. After the cells became stable, the ligated solutions were transfected into HeLa cells using Lipofectamine 3000 (Thermo Fisher Scientific Inc.) as the manufacturer described. Briefly, 5 µl of heat-inactivated ligated solution was mixed with 0.3 µl Lipo3000, 1 µl P3000, and OPTI-MEM up to 10 µl, followed by incubation at room temperature for 15 min, and then added to HeLa cells. After 6 h incubation at 37°C under 5% CO_2_ atmosphere, DMEM containing lipofectamine solution was replaced with fresh DMEM. When changing solution in all subsequent steps, two- or three-times wash with phosphate-buffered saline (PBS) was performed. After an additional 18 h incubation (24 h from transfection), DMEM was removed and replaced with 4% paraformaldehyde (PFA) in PBS to fix cells. After 25 min incubation at room temperature, PFA was removed and replaced with PBS for microscopic observation by benchtop fluorescence microscopy, BZ-X810 (Keyence).

## Results and discussion

### Synthesis of 3′-thionucleoside phosphoramidites and 3′-phosphorothiolate DNAs

**Scheme 1.**
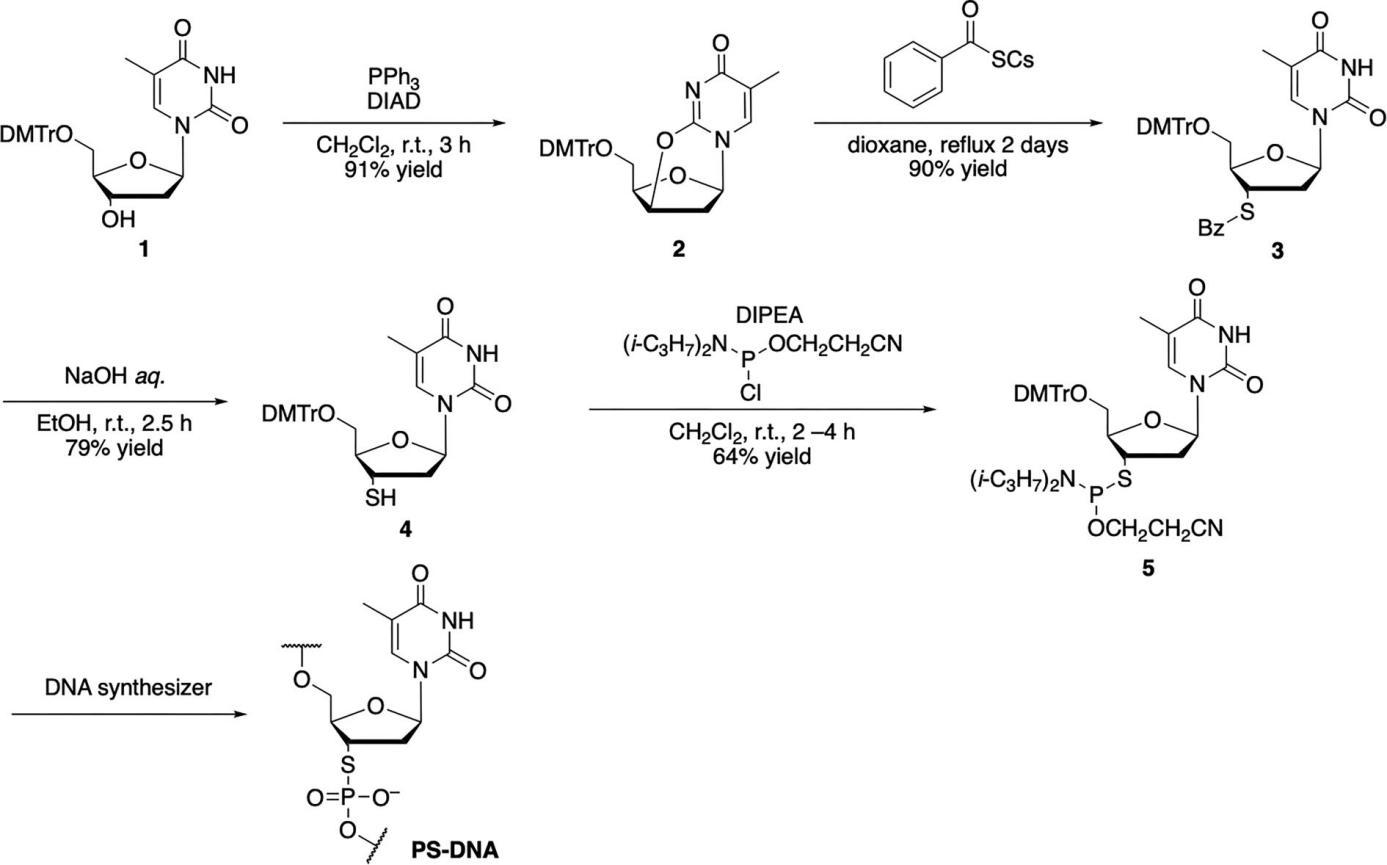
Synthesis of 3′-thiothymidine phosphoramidite (**5**) and 3′-phosphorothiolate oligodeoxyribonucleotides (3′S-DNA)^a^. *^a^* Reaction conditions and yields, as indicated. DMTrCl, 4,4′-dimethoxytrityl chloride; DMAP, 4-Dimethylaminopyridine; DIAD, Diisopropyl azodicarboxylate; Bz, Benzoyl; Et, ethyl; DIPEA, *N, N*-diisopropylethylamine.

Synthesis of 3′-thionucleoside phosphoramidites with all four bases has been reported in 2020 [20]. We started the synthesis according to the literature. The synthesis of thymidine derivative has been reported in several papers [13–15, 20, 21], and we conducted the synthesis according to Scheme 1. Cyclization of commercially available 5′-O-DMTr-protected thymidine (Scheme 1-**1**) between the 2-carbonyl oxygen and 3′-carbon by treatment with triphenyl phosphine and diisopropyl azodicarboxylate (DIAD) in dichloromethane gave *O*-2,3′-cyclothymidine derivative (Scheme 1-**2**) in 91% yield. The thiocarbonyl group at the 3′-position of compound *O*-2,3′-cyclothymidine derivative (Scheme 1-**2)** was introduced by treatment with cesium thiobenzoate in dioxane under refluxing conditions to afford 3′-thiobenzoyl thymidine derivative (Scheme 1-**3**) in 90% yield. In this reaction, we first tested reflux with cesium thiobenzoate in DMF, following the conditions described in the literature [13–15, 20, 21]. However, we found that thiobenzoic acid and thiobenzoate were desulfurized by reaction with DMF under high-temperature conditions and produced 3′-oxybenzoyl substituted side-product (Supplementary Figs S1, S2, and S5, and Supplementary Scheme S5). The side-product was difficult to remove using normal-phase silica gel column chromatography (Supplementary Figs S3 and S4). Therefore, we screened several solvent systems, such as acetonitrile and dioxane, for the 3′-thiobenzoylation reaction to prevent the side-reaction and to obtain good target compound yields (Supplementary Fig. S1). As a result, the reaction in dioxane showed an excellent yield of the target 3′-thiobenzoylated thymidine analog (Scheme 1-**3**) without any side-product formation. 3′-Thiobenzyl-thymidine (Scheme 1-**3**) was then treated with degassed aqueous sodium hydroxide in ethanol to give the 3′-thiothymidine derivative (Scheme 1-**4**) in 79% yield. The phosphitylation of compound 3′-thiothymidine derivative (Scheme 1-**4**) was performed by the reaction with 2-cyanoethyl diisopropylchlorophosphoramidite in the presence of *N,N*-diisopropylethylamine in dichloromethane, and 3′-thiothymidine phosphoramidite (Scheme 1-**5**) was obtained in 64% yield. Using 3′-thiothymidine phosphoramidite (Scheme 1-**5**), we synthesized DNAs bearing multiple 3′-phosphorothiolate linkages using an automated DNA synthesizer based on standard phosphoramidite chemistry [20]. The synthesized DNAs were purified by reverse-phase high-performance liquid chromatography and characterized by Matrix-Assisted Laser Desorption/Ionization Time-of-Flight Mass Spectrometry (MALDI-TOF-MS) and dPAGE (Supplementary Figs S6–S14 and  Supplementary Table S1). The 3′-thionucleoside phosphoramidites with guanine, adenine, and cytosine analogs were also synthesized (Schemes S1–S4). In the synthesis of pyrimidine derivatives (compounds 5 and 10 of Supplementary Schemes S1 and S2), phosphitylation was carried out using 1.1–1.3 equivalents of 2-cyanoethyl diisopropylchlorophosphoramidite. Minimizing the amount of phosphitylating reagent effectively suppressed the detritylation side-reaction caused by decomposition of the phosphitylation reagent. In contrast, for the synthesis of purine derivatives (compounds 16 and 23 of Supplementary Schemes S3 and S4), phosphitylation was performed with 1.5 equivalents of 2-cyanoethyl *N,N,N*',*N*'-tetraisopropylphosphordiamidite, affording the corresponding phosphoramidite derivatives in good yields [20].

### 3′-Phosphorothiolate DNA strand cleavage by silver nitrate treatment

Cosstick [13] and Vyle *et al*. and coworkers [14] have reported that DNA with 3′-phosphorothiolate is site-specifically cleaved by silver nitrate treatment to produce a DNA fragment with 5′-phosphate. We first proposed the cleavage of double-stranded DNA with a 3′-phosphorothiolate bond and prepared a sticky end using silver nitrate. To demonstrate the strand cleavage system, a 20-nt 3′-FAM-labeled 3′-phosphorothiolate DNA (3′*S*-DNA1: 5′-TAAC**Ts**CACATTAATTGCGTT-FAM-3′, **Ts** denotes 3′-phosphorothiolate) was treated with silver nitrate, and the progress of the cleavage reaction was analyzed by dPAGE (Supplementary Fig. S15). It was confirmed that the 3′-phosphorothiolate bond was rapidly cleaved by treatment with silver nitrate at ambient temperature for 5 min. Subsequently, the cleavage product was identified using MALDI-TOF-MS analysis of the cleavage product treated with silver nitrate. According to the analysis, the presence of the desired cleavage products, DNA with 5′-phosphate and DNA with 3′-end thiol group, was confirmed. However, MALDI-TOF-MS peaks were observed as clustered multiple silver ion adducts with a molecular weight distribution (Supplementary Fig. S16). Silver ions are strongly bound to chloride ions, Cl^−^, contained in the buffer used for physiological conditions and biochemical experiments, and poorly water-soluble AgCl starts to precipitate [22]. Therefore, silver ions must be removed from the reaction solution for strand cleavage before moving toward DNA fragment ligation. Thiol compounds were added after the cleavage reaction was complete to remove silver ions from the reaction solution. Therefore, the strand cleavage reaction was quenched by adding DTT or EtSH, and the silver–thiol precipitate produced was removed by centrifugation. The supernatant was collected and analyzed using dPAGE to confirm the progress of the cleavage reaction (Supplementary Fig. S17). Furthermore, the amount of DNA contained in the supernatant after removal of the silver ion as a precipitate by adding thiol compounds was quantified by measuring the absorbance at 260 nm derived from the DNA. The total amount of DNA per reaction solution was 220 pmol, but the amount of DNA recovered from the cleavage product was 92 pmol for the DTT-added system and 88 pmol for the EtSH-added system. This result indicates that the precipitation-based silver ion removal method using thiol compounds causes a loss of ~60% of the cleaved DNA product by co-precipitation with silver–thiol complexes. According to the experimental results, it is difficult to apply a silver ion cleavage system for the practical sticky-end preparation method required for long-chain DNA assembly, in which silver ions remain in the solution even if the DNA cleavage reaction occurs with high efficiency.

### Silver nanoparticle-mediated strand cleavage

**Figure 2.**
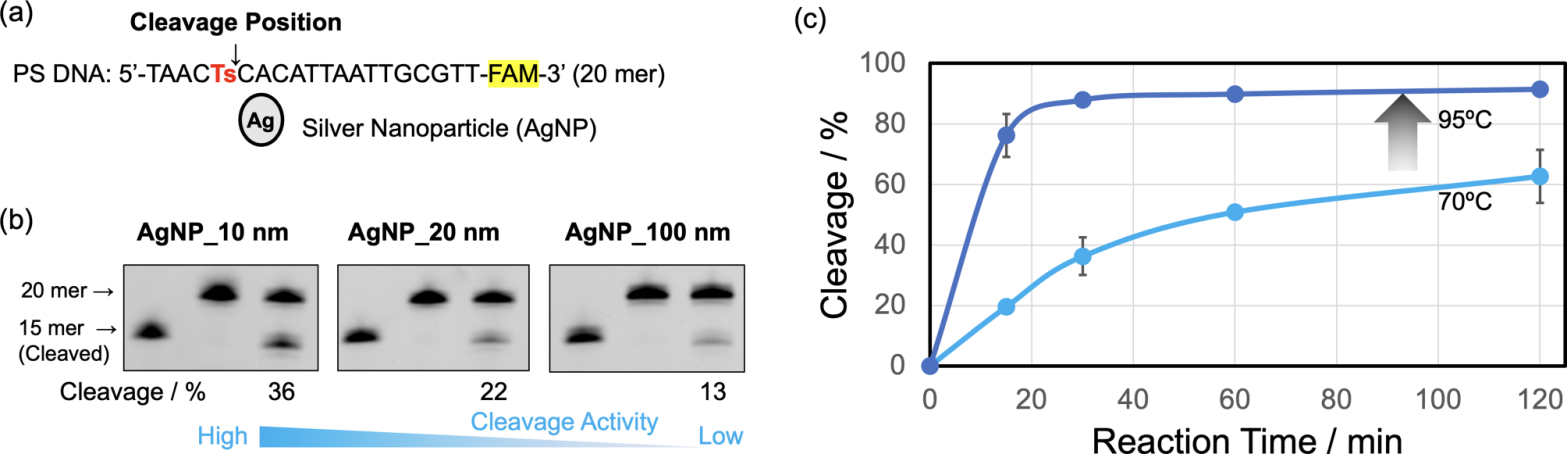
AgNPs induce DNA strand cleavage reactions and their nanoparticle size/reaction time dependencies. (**a**) Sequence and cleavage positions of AgNPs. The cleavage position is denoted as allowed. 3′-phosphorothiolate-linked thymidine position in the sequence is denoted as Ts. DNA has a 3′-FAM modification as a fluorescent dye to visualize DNA on gel analysis. (**b**) dPAGE analysis indicating AgNP size dependency on DNA strand cleavage. AgNPs with diameters of 10, 20, and 100 nm were tested for the reaction. Reaction condition: 0.50 µM of 3′S-DNA and AgNPs (0.017 mg/ml) in H_2_O at 37°C for 31 h. (**c**) Time course of the strand cleavage induced by AgNPs. Reaction conditions: 0.50 µM of 3′S-DNA and AgNPs (0.017 mg/ml; 10 nm) in H_2_O at 70°C or 95°C for 0–2 h. Data are presented as means (*n* = 3). Error bars indicate variability among three independent experiments.

In this study, we developed a more practical 3′-phosphorothiolate linkage cleavage method using AgNPs. AgNPs are commercially available and have a wide range of particle sizes. Considering the surface area of ​​the particles to which the cleavage substrate is bound, nanoparticles with a smaller particle diameter exhibit higher cleavage activity, and nanoparticles with a larger particle diameter exhibit lower cleavage activity [23]. Therefore, we investigated the cleavage reaction of 3′*S*-DNA1 and the nanoparticle size dependency for the strand cleavage reaction using AgNPs with particle sizes of 10, 20, and 100 nm (Fig. 2a and b). The reaction was performed at 37°C for 31 h, the reaction solution was analyzed by dPAGE, and the strand cleavage efficiency was calculated from the band intensity of the dPAGE analysis by FAM-derived fluorescence detection. The desired strand cleavage products were observed under all conditions, and the cleavage efficiencies when 10, 20, and 100 nm AgNPs were used were 35.9%, 21.9%, and 13.1%, respectively. As expected, the smaller AgNPs showed higher strand cleavage activity (Fig. 2b). Next, the reaction time and temperature dependence of DNA strand cleavage by AgNPs were investigated. The strand cleavage reaction of 3′*S*-DNA1 was performed at 70°C or 95°C using AgNPs with a diameter of 10 nm. The reaction was carried out for 15, 30, 60, and 120 min, and each reaction solution was analyzed by dPAGE. The strand cleavage efficiency at each temperature and time was calculated from the band intensity of the gel using FAM-derived fluorescence detection. The reaction time versus strand cleavage efficiency plot is shown in Fig. 2c. According to the results, a reaction time-dependent enhancement of strand cleavage efficiency was observed, and ~50% of strand cleavage was observed at 70°C after 2 h of treatment. By increasing the reaction temperature from 70°C to 95°C, the cleavage efficiency was dramatically improved, and the DNA was almost quantitatively cleaved after 2 h of treatment at 95°C. This result suggests that commercially available nanoparticles require high-temperature conditions to induce efficient DNA strand cleavage (Fig. 2c and Supplementary Fig. S19).

**Figure 3.**
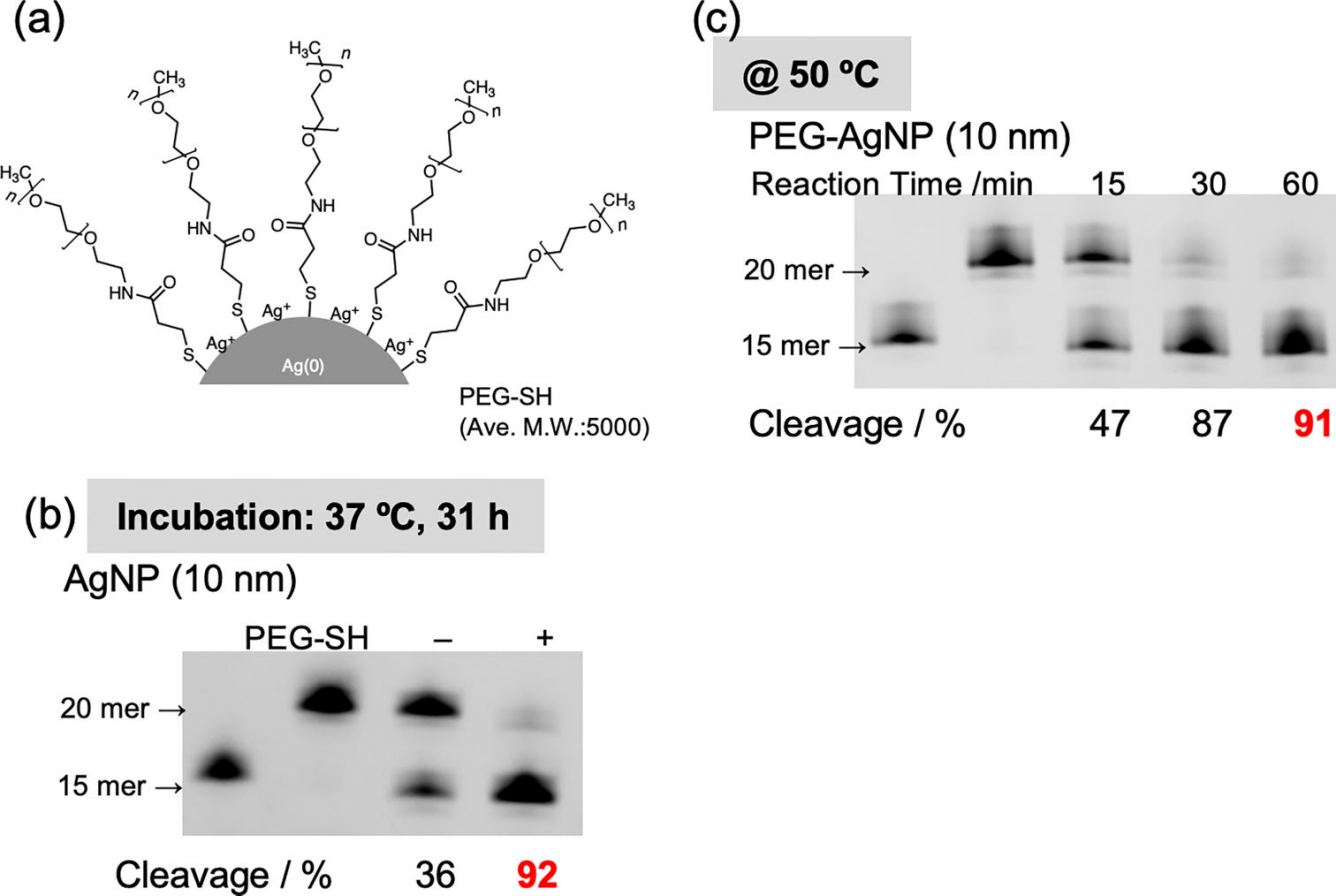
PEGylated AgNPs enhance DNA strand cleavage activity. (**a**) Illustration of PEGylated AgNPs. Commercially available thiol-terminated PEG, *O*-[2-(3-mercaptopropionylamino)ethyl]-*O*’-methylpolyethylene glycol (PEG-SH, average molecular weight 5 000), was used for nanoparticle surface modification. (**b**) dPAGE analysis indicating the effect of PEGylation of AgNPs of 10-nm particle size on the DNA strand cleavage reaction. The reaction was performed with 0.51 µM of 3′S-DNA in a 0.017-mg/ml AgNP dispersion with or without nanoparticle surface PEGylation at 37°C for 31 h. The PEGylated AgNPs were prepared by mixing the commercially available 0.02 mg/ml AgNP dispersion (50 µl) and 23.9 g/l PEG-SH aqueous solution (1.0 µl). (**c**) dPAGE analysis indicating reaction time dependency of 3′S-DNA strand cleavage using PEGylated AgNPs of 10-nm particle size. The reaction was performed with 0.51 µM of 3′S-DNA in a 0.017-mg/ml PEGylated AgNP dispersion at 50°C for 0–60 min. The PEGylated AgNPs were prepared by mixing the commercially available 0.02 mg/ml AgNP dispersion (50 µl) and 23.9 g/l PEG-SH aqueous solution (1.0 µl).

Considering that the DNA strand cleavage method is applied to long-chain DNA synthesis tools, there is concern about non-specific degradation under high-temperature conditions, such as 90°C [24]. Therefore, it is necessary to optimize the reaction conditions to effectively cleave DNA strands at a temperature as low as possible. To satisfy this requirement, we focused on polymer modification of the surface of the nanoparticles. Šimáková *et al*. reported the stabilization of AgNPs against surface oxidation and aggregation by a PEG derivative [25]. We expected that PEGylation of AgNPs would enhance their DNA strand cleavage activity owing to their oxidation resistance and improved dispersion properties in aqueous media. Based on this consideration, we prepared PEGylated AgNPs by adding PEG with an average molecular weight of 5000 modified with terminal thiols, *O*-[2-(3-mercaptopropionylamino)ethyl]-*O*’-methylpolyethylene glycol, to bind with the AgNP surface and subjected to the DNA strand cleavage reaction (Fig. 3a). Using AgNPs with a diameter of 10 nm, the DNA strand cleavage activity with and without surface PEGylation was compared (Fig. 3b). Reactions were performed at 37°C for 31 h. The reaction solutions were analyzed by dPAGE, and strand cleavage efficiency was calculated from the band intensity of the gel by FAM-derived fluorescence detection. The strand cleavage efficiency of the AgNPs was 35.9% without PEGylation, whereas the cleavage activity of PEGylated AgNPs was 91.8%. Based on these results, it can be concluded that PEGylation of AgNPs significantly enhances DNA strand cleavage activity. This effect of improving the cleavage activity is explained by the prevention of surface oxidation of AgNPs by PEGylation and the improvement of dispersibility in an aqueous solution (Supplementary Figs S21 and S22, proof of improvement in AgNP dispersity by UV spectral analysis). Next, we investigated the reaction time dependency of DNA strand cleavage using PEGylated AgNPs with a diameter of 10 nm (Fig. 3c). The reaction was performed at 50°C for 15, 30, and 60 min, and each reaction solution was analyzed by dPAGE. The strand cleavage efficiency at each reaction time was calculated from the band intensity of the gel by detecting the fluorescence derived from FAM. As a result, enhancement of strand cleavage activity was observed with increasing reaction time, and >90% cleavage was observed within 60 min of the treatment. From this result, PEGylation of commercially available AgNPs can express highly efficient DNA strand cleavage activity under relatively milder temperature conditions.

The amount of recovered DNA after strand cleavage was quantified, and the recovery yields were compared for AgNPs and silver nitrate treatments. An almost quantitative strand cleavage was observed by treating 3′S-DNA at 90°C for 25 h with AgNPs with a particle size of 100 nm (Supplementary Fig. S18). The AgNPs in the reaction solution were completely removed as a precipitate by centrifuging at 15 000 rpm for 1 h (Supplementary Fig. S20). The supernatant was collected, the absorption spectrum was measured, and the amount of cleaved DNA fragment was calculated from the absorbance at 260 nm. On the other hand, in the case of silver nitrate treatment, silver ions were removed as a precipitate by adding DTT to the reaction solution after the strand cleavage, the supernatant was collected, and the cleaved DNA fragment was quantified. The total amount of DNA per reaction solution was 244 pmol, whereas in the case of silver nitrate treatment, the amount of recovered DNA fragment was 35.1 pmol, and the recovery yield was only 14.4%, indicating that most of the cleavage products were co-precipitated with the silver–DTT complex to decrease the yield. In contrast, in the case of AgNP treatment, the amount of recovered DNA fragment was 240 pmol, which means that the cleavage product DNA was recovered almost quantitatively (Table 1 and Supplementary Table S2). From this result, it is difficult to practically use silver nitrate treatment for the 3′-phosphorothiolate DNA strand cleavage method because the recovery yield of DNA fragments is significantly decreased. In contrast, strand cleavage using AgNPs is a promising method to recover cleaved DNA fragments with high efficiency.

**Table 1.** Output of DNA fragment after 3′-phosphorothiolate bond cleavage (244 pmol scale reactions)

Additives	Cleaved DNA	Recovery yield
AgNO_3_	35.1 pmol	14.4%
AgNP (100 nm)	240 pmol	98.4%

### Characterization of AgNPs for DNA strand cleavage

We have optimized the conditions for DNA cleavage by AgNPs. Notably, PEGylation on the AgNP surface significantly enhanced their DNA cleavage activity. Therefore, we decided to analyze the surface properties of the AgNPs used in the DNA cleavage reaction. Furthermore, by examining the state of the AgNP surface before and after the DNA cleavage reaction, we can demonstrate that the DNA was cleaved as intended and that the resulting fragments bearing terminal thiol groups were captured on the AgNP surface. Physical parameters such as particle size, size distribution, and ζ-potential were obtained using dynamic light scattering (DLS) with a Zeta-Sizer, enabling us to evaluate the characteristics of AgNPs and discuss the mechanism of the DNA cleavage reaction.

First, we evaluated the characteristics of AgNPs before and after PEGylation. The particle sizes of commercially available AgNPs (10 and 20 nm) were measured, yielding consistent values of 11.42 and 19.23 nm, respectively. Upon addition of *O*-[2-(3-mercaptopropionylamino)ethyl]-*O*'-methylpolyethylene glycol, the particle size of 10-nm AgNPs increased from 11.42 to 22.22 nm, and that of 20-nm AgNPs increased from 19.23 to 34.33 nm (Table 2 and Supplementary Fig. S23). Furthermore, ζ-potential measurements revealed a substantial positive shift upon PEGylation for both 10- and 20-nm AgNPs (10 nm: −31.72 to −6.83 mV; 20 nm: −28.25 to −2.63 mV). Typically, AgNPs carry a positive charge; however, citrate bound to their surface introduces negative charges, which are observed as ζ-potential. When AgNPs are PEGylated, the particle size increases and the charge-shielding effect of the neutral PEG molecules shifts the ζ-potential toward near-neutral values. These results demonstrate that the addition of *O*-[2-(3-mercaptopropionylamino)ethyl]-*O*'-methylpolyethylene glycol alone is sufficient to confirm PEGylation of the AgNP surface.

**Table 2.** DLS analysis results for AgNP characterization

Sample	Zeta potential (mV)	Particle size (nm)	PDI
AgNP (10 nm)	−31.72	11.42	0.3906
AgNP (10 nm)–DNA Cleavage	−51.05	12.13	0.4860
PEGylated AgNP (10 nm)	–6.83	22.22	0.3242
PEGylated AgNP (10 nm)–DNA Cleavage	−18.68	28.04	0.2863
AgNP (20 nm)	−28.25	19.23	0.2504
AgNP (20 nm)–DNA Cleavage	−50.67	24.23	0.4075
PEGylated AgNP (20 nm)	−2.63	34.33	0.2110
PEGylated AgNP (20 nm)–DNA Cleavage	−19.96	31.69	0.2490

The term “–DNA Cleavage” denotes that the DLS measurement was conducted following the DNA strand cleavage reaction, implying that the cleaved DNA fragments became associated with the AgNP surface.

**Figure 4.**
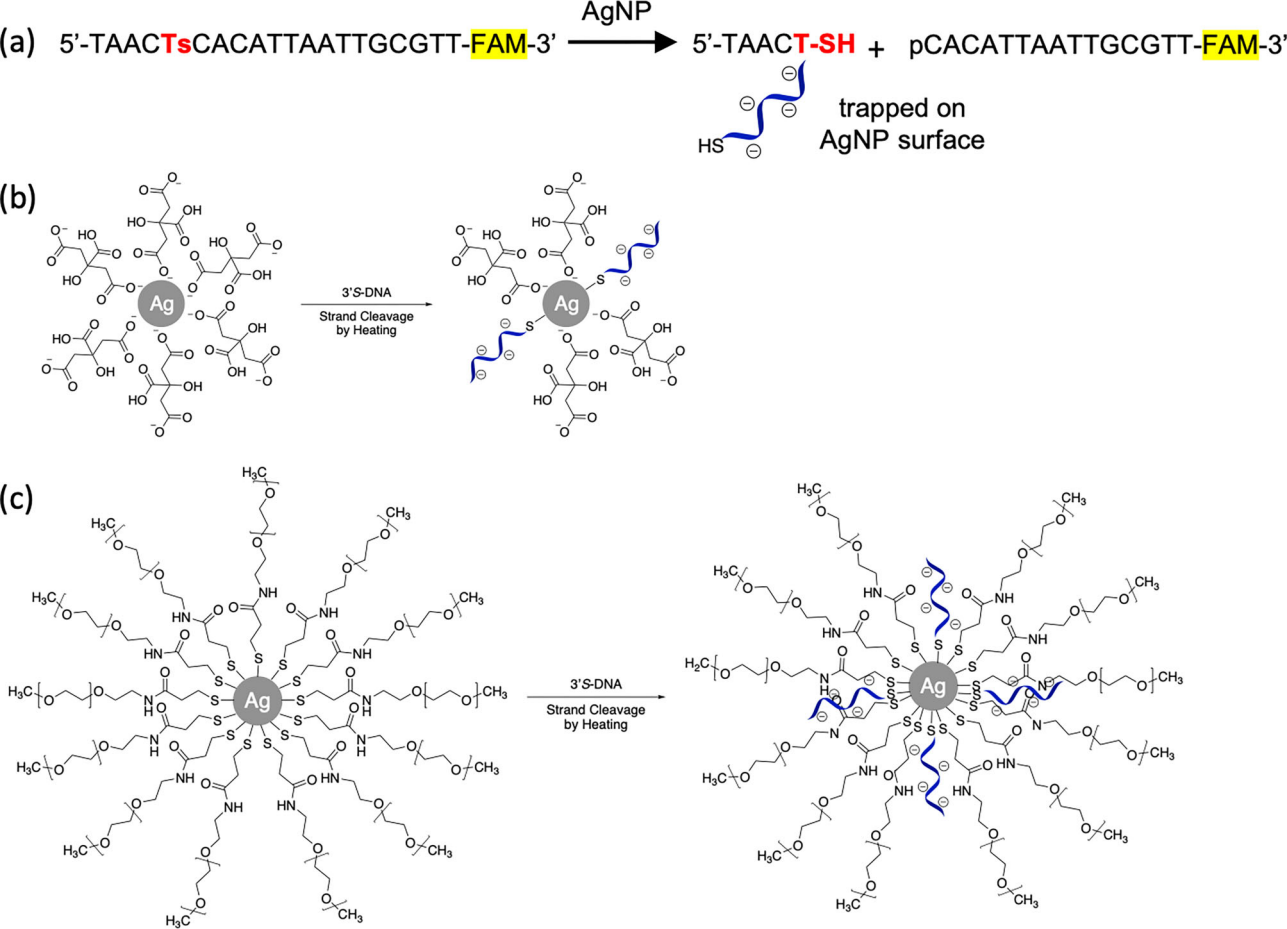
Schematic illustration of DNA strand cleavage for DLS analysis of AgNP presented in Table 2. (**a**) Sequences of the target DNA and the resulting cleaved fragments. (b) Surface structure of AgNPs before and after DNA strand cleavage. (**c**) Surface structure of PEGylated AgNPs before and after DNA strand cleavage. Following cleavage, the electronegative DNA fragments become adsorbed onto the AgNP surface, altering its properties.

Next, we evaluated the characteristics of AgNPs before and after DNA cleavage (Fig. 4 and Supplementary Fig. S24). A 20-nt 3′-FAM-labeled 3′-phosphorothiolate DNA (3′S-DNA1: 5′-TAAC**Ts**CACATTAATTGCGTT-FAM-3′, where **Ts** denotes 3′-phosphorothiolate) was added to an AgNP dispersion and subjected to cleavage by heating at 95°C for 1–2 h (AgNP) or 50°C for 1–2 h (PEGylated AgNP). After the reaction, DLS measurements were performed (Supplementary Fig. S23). In all cases, a marked increase in particle size and a substantial negative shift in ζ-potential were observed after DNA cleavage compared to before (Fig. 4 and Table 2). These results can be explained by the capture of cleaved DNA fragments (5′-TAAC**Ts**-SH) on the AgNP surface, which increased particle size and introduced negative charges from the phosphate diester backbone, thereby shifting the ζ-potential toward more negative values. Thus, it was confirmed that, as expected, DNA fragments were captured on AgNPs following cleavage. The polydispersity index (PDI) remained within the range of 0.3–0.5 for all AgNPs, regardless of PEGylation or DNA cleavage, indicating a polydispersed state. A slight increase in PDI was observed after DNA cleavage, suggesting that the capture of DNA fragments on AgNPs broadened the size distribution.

To discuss the DNA cleavage mechanism in more detail, we measured the residual silver ion concentration in the AgNP dispersion. The AgNPs used in this study were synthesized by the citrate reduction method [26]; however, the manufacturer did not specify the residual silver ion concentration. Using an assay based on the oxidation of 3,3′,5,5′-tetramethylbenzidine (TMB) [27], the concentration was estimated to be ~12 ppm (Supplementary Figs S25 and S26). It should be noted that the TMB-based quantification method cannot distinguish between residual silver ions and silver ions present on the nanoparticle surface, as both contribute to TMB oxidation [27]. Meanwhile, ζ-potential and particle size measurements before and after DNA cleavage clearly indicate that the cleaved DNA fragments are captured on the AgNP surface (Table 2). Furthermore, the data in Fig. 2b show that cleavage efficiency varies depending on AgNP size, suggesting that DNA cleavage is driven primarily by AgNP activity rather than residual silver ions. Taken together, these findings indicate that DNA strand cleavage may involve both the nanoparticle surface and residual silver ions but predominantly occurs at the AgNP surface. Regardless of the cleavage pathway, the resulting DNA fragments are trapped by AgNPs, and only fragments with cohesive ends are released, making them effectively available for ligation.

### Production of sticky ends and ligation to build up long-chain DNA

For AgNP-mediated DNA strand cleavage technology to apply to long-chain DNA synthesis, the synthesized 3′-phosphorothiolate-linked DNA must act as a PCR substrate and function effectively as primers for amplification. In addition, the PCR-amplified double-stranded DNA containing the 3′-phosphorothiolate linkage needs to be position-specifically cleaved by AgNP treatment to form a sticky end with several base overhangs.

First, to confirm that the 3′-phosphorothiolate linkage DNA does not inhibit recognition by the polymerases used for PCR, primer extension experiments were performed using template DNA containing a 3′-phosphorothiolate linkage. A 22-mer 5′-FAM DNA primer was annealed to the template DNA containing a 3′-phosphorothiolate linkage. Then, various polymerases, KOD-Plus-Neo, PrimeSTAR HS, Phusion High Fidelity, Q5 High Fidelity, and Deep Vent Taq DNA polymerase, were added to start the primer extension reactions. If the extension stops at the 3′-phosphorothiolate modification site, a product with 4 or 5 extended bases is produced from the 22-nt primer. On the other hand, when the 3′-phosphorothiolate modification site is extended without any problems, a full-length 41-nt DNA is produced. After the primer extension reaction, the reaction solution was analyzed by dPAGE, and the band was visualized by a gel image analyzer using fluorescence detection derived from 5′-FAM. As a result, the bands derived from the extended product DNA with a full length of 41-nt were observed for all polymerases, and no elongation arrest product was observed at the 3′-phosphorothiolate modification site (Supplementary Fig. S28). Based on this result, it was confirmed that the 3′-phosphorothiolate linkage DNA can be accepted and applied as PCR primers.

Next, we evaluated whether the double-stranded DNA could be position-specifically cleaved by AgNP treatment in the same manner as single-stranded 3′S-DNA cleavage to form overhang structures. Cleavage of 20-nt double-stranded DNA with a 3′-phosphorothiolate linkage was performed using PEGylated AgNPs with a particle size of 10 nm. The 3′-phosphorothiolate linkage is located between the fifth and sixth bases from the 5′-end and is designed so that when cleavage occurs, a sticky end with 5 bases overhang can be prepared. The reactions were performed at 50°C for 15, 30, and 60 min. Each reaction solution was analyzed by dPAGE, and the strand cleavage efficiencies at each reaction time point were calculated from the band intensity of the gel by detecting the fluorescence derived from FAM. The cleavage efficiencies with and without complementary strands are compared, and the cleavage efficiencies at each reaction time are plotted (Supplementary Fig. S27). In the presence of the complementary strand, the cleavage activity was slightly reduced compared to that in the absence of the complementary strand; however, the cleavage activity was almost the same after 2 h of treatment, and >90% of the DNA was cleaved. According to this result, it was clarified that both single-stranded and double-stranded DNA can be effectively cleaved by PEGylated AgNPs and can be applied to the preparation of DNA fragments with sticky ends.

**Figure 5.**
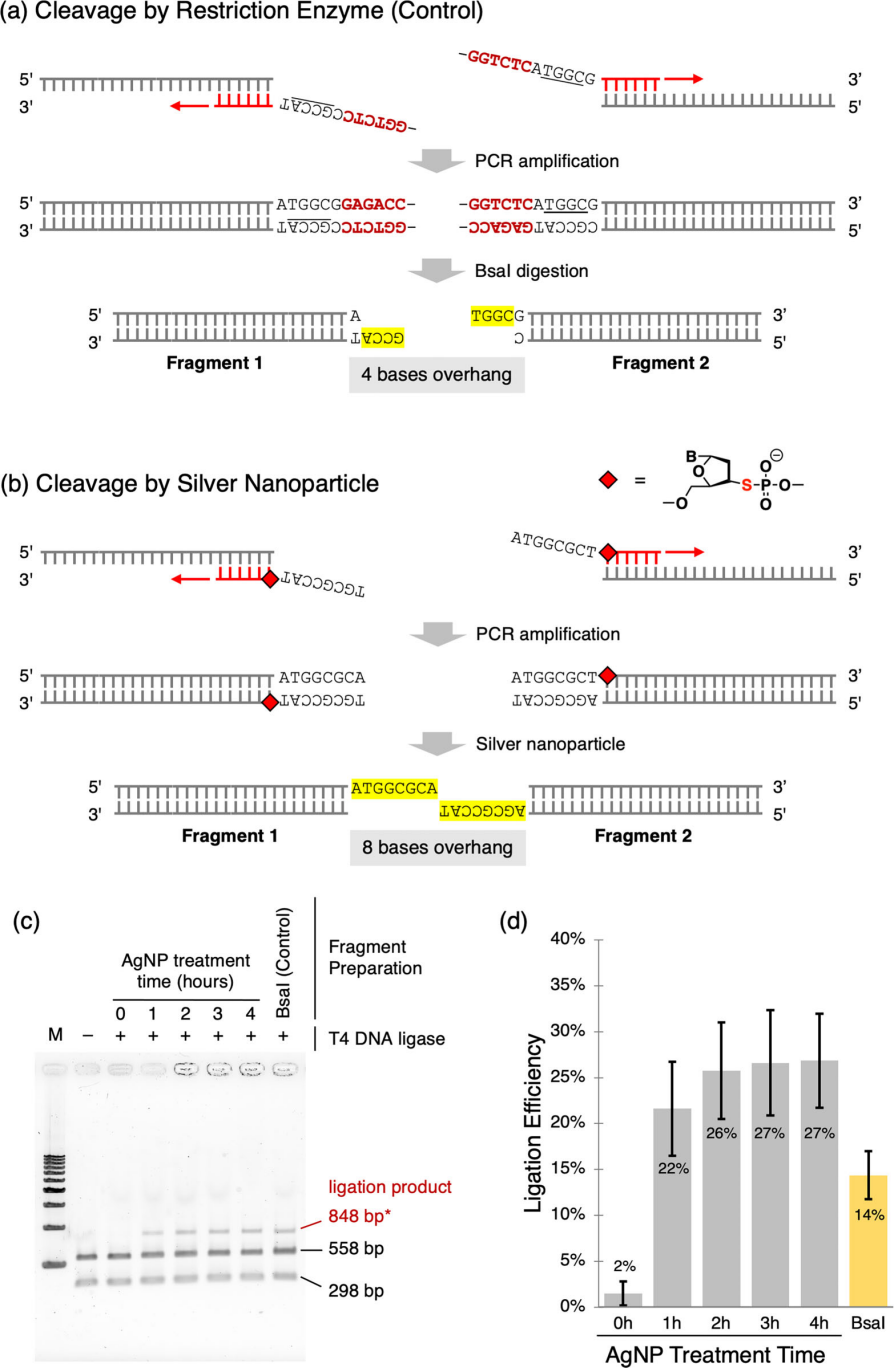
Experimental design of DNA fragment production by (**a**) cleavage using restriction enzymes and (**b**) cleavage using AgNPs for long-chain DNA assembly. In the case of restriction enzyme cleavage, sticky ends with four-bases overhang are produced. In contrast, sticky ends with eight-bases overhang can be produced using AgNP-induced strand cleavage technology. The digestion site of BsaI is underlined, and the recognition site of BsaI is indicated in bold red capital. Yellow markers indicate overhang regions. Ligation of DNA fragments with sticky ends prepared by AgNP treatment. (**c**) Agarose gel analysis of the ligation reaction of DNA fragments by T4 DNA ligase. DNA fragments with sticky ends were prepared by PEGylated AgNP treatment for 0, 1, 2, 3, and 4 h or BsaI treatment. PCR by using 3′S-DNA primers was conducted with 0.50 µM of 3′S-DNA forward primer, 0.50 µM of 3′S-DNA reverse primer, 0.50 ng/µl template DNA, 0.20 mM dNTP mixture, 1.5 mM MgSO_4_, 1× PCR buffer, and KOD-Plus-Neo (1 U). After PCR, the amplified 7.5 nM of 3′S-DNA was cleaved by 0.017 mg/ml PEGylated AgNPs with 10-nm particle size at 50°C for 1– 4 h to prepare sticky ends. Ligation of DNA fragments with sticky ends was carried out with 3.0 nM DNA fragment1, 3.0 nM DNA fragment2, 1× Reaction buffer, and T4 DNA ligase (900 U) at 25°C for 3 h. (**d**) Graph indicating ligation efficiencies of sticky-end DNA fragments prepared by AgNP or BsaI treatments. Data are presented as means (*n* = 4). Error bars indicate variability among four independent experiments.

Based on these experiments, a primer DNA with a 3′-phosphorothiolate linkage at the cleavage site was used for PCR amplification of DNA, followed by AgNP treatment to prepare the sticky end and DNA fragment ligation by T4 DNA ligase. We designed and synthesized PCR primers with a 3′-phosphorothiolate linkage at the cleavage site to produce eight-base overhang sticky ends after PCR amplification, followed by AgNP treatment. PEGylated AgNPs with a particle size of 10 nm were used. A system was designed in which the PCR product DNA, when a natural DNA primer was used as a control, was cleaved by the restriction enzyme, BsaI, to generate a four-base overhang sticky end (Supplementary Table S3 and Fig. 5a).

By using 3′S-DNA3 (Fw for F1) and 3′S-DNA4 (Rev for F2) with 3′-phosphorothiolate linkage, PCR amplification with KOD-Plus-Neo was performed in the presence of template DNA, and two types of PCR products, 298 base pairs and 558 base pairs, were obtained (Fig. 5b and Supplementary Fig. S29). The PCR products were purified using a Wizard column (PROMEGA). PEGylated AgNPs were then added to the PCR products, and the mixture was treated at 50°C for 1–4 h to cleave the 3′-phosphorothiolate linkage to prepare the sticky end with eight bases overhang. T4 DNA ligase was added to the DNA fragments with two sticky ends, and the DNA fragments were ligated by treatment at 25°C for 3 h. The reaction solution was analyzed by 1% agarose gel electrophoresis (electrophoresis buffer: 1× TBE, 100 V, 30 min), and the ligation efficiencies of T4 DNA ligase were evaluated. Gel analysis confirmed that 848 bp of the ligated product was successfully synthesized under all the ligation reaction conditions (Fig. 5c). Figure 5d shows the yields of the product obtained by ligation of DNA fragments with eight-base overhang sticky ends, which were prepared by PEGylated AgNP treatment of PCR products for 1, 2, 3, and 4 h. As a control, the ligation yield of DNA fragments with four-base overhang sticky ends, which were prepared by BsaI treatment, is also indicated in Fig. 5d (Supplementary Fig. S30). According to this result, as the AgNP treatment time increased, the ligation yields also increased. When the DNA fragments prepared by AgNP treatment for 240 min were ligated, the ligation efficiency was higher than that of the DNA fragments prepared by BsaI treatment. This result indicates that sticky ends with longer overhangs can give higher ligation efficiency. In the case of restriction enzyme treatment, only four bases of overhang can be prepared; in contrast, we can prepare sticky ends of any length of overhang by applying AgNP-mediated strand cleavage technology.

### Construction of functional proteins

**Figure 6.**
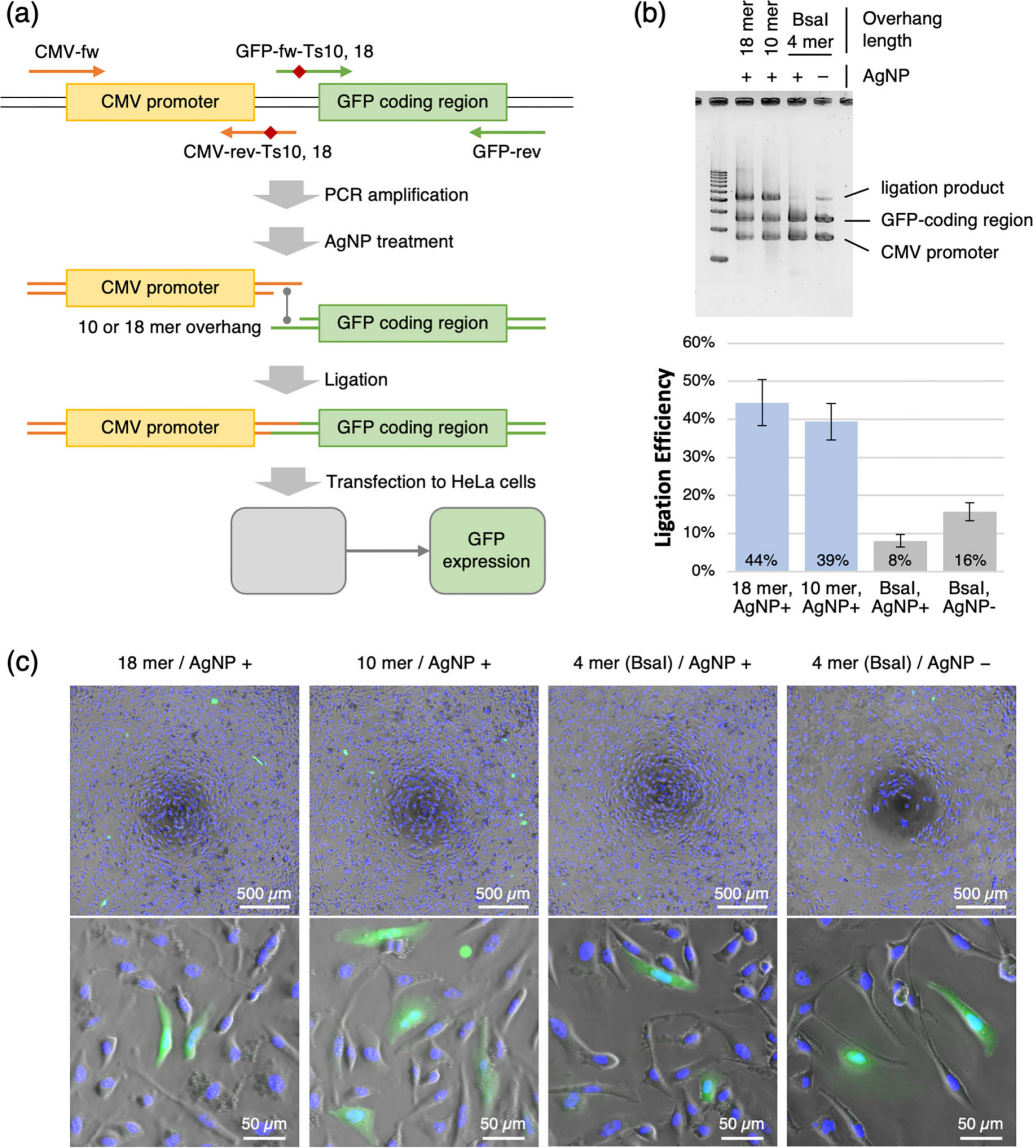
Transfection of ligated products into HeLa cells. (**a**) Experimental design and procedure. (**b**) Agarose gel analysis of the ligation reaction of the DNA fragments with 18, 10, and 4 (BsaI-treated) bases overhanging sticky ends. The ligation efficiency of the BsaI-treated fragments was compared in the presence and absence of AgNPs. The graph below shows the ligation efficiencies calculated from the ratio of the band intensities. Data are presented as means (*n* = 6 or 7). Error bars indicate variability among six or seven independent experiments. (**c**) Microscopic images of HeLa cells transfected with ligation reaction mixtures are shown in panel (b). Images in the top and bottom panels were captured using BZ-X800 (Keyence) with 4× and 20× objective lenses, respectively.

We confirmed the applicability of this strand cleavage technology in the construction of an expression system for functional proteins. Here, two fragments carrying the CMV promoter or GFP coding sequence were amplified by PCR with PCR primers containing 3′-phosphorothiolate to produce sticky ends after PCR amplification and following AgNP treatment (Fig. 6a and Supplementary Figs S31 and S32). GFP expression in HeLa cells transfected with the ligated product of the cleaved fragments will prove accurate construction using our technology.

In this experiment, it is necessary to process 828- or 832-bp dsDNAs encoding the CMV promoter and 1 264- or 1 252-bp dsDNAs encoding GFP with AgNPs to generate sticky ends. Therefore, we examined whether the interaction between such long dsDNA molecules and AgNPs could be confirmed by DLS. When the 1 264-bp dsDNA encoding GFP was added to an AgNP dispersion and analyzed by DLS, an increase in particle size and a negative shift in ζ-potential were observed (Supplementary Fig. S35). In particular, the increase in total counts in Supplementary Fig. S35c indicates that the long dsDNA interacted with AgNPs, increasing in particle size. These results indicate that even long dsDNA molecules, such as those with 1 264 bp, can interact with AgNPs and effectively induce strand cleavage without any issues.

Two primer sets to produce 18 or 10 bases overhang sticky ends were synthesized to validate the effect of overhang length on ligation efficiency (Table 3). In primers making 18 bases overhang, 3′-phosphorothiolate was inserted at two sites to shorten the cleaved fragments to promote their dissociation from the overhang region. These CMV-rev-18, CMV-rev-10, GFP-fw-18, and GFP-fw-10 primers were synthesized by using 3′-thiothymidine phosphoramidite (Scheme 1-5) (Supplementary Figs S10–S13). The PCR products obtained using these primers were purified by the Wizard SV Gel/PCR Clean-Up system and treated with PEGylated AgNPs at 50°C for 4 h to cleave the 3′-phosphorothiolate linkages to prepare the sticky ends with 10 or 18 bases overhangs. Approximately 830 and 1 260 base pairs of products with the sticky ends were obtained as the CMV promoter and GFP coding sequence, respectively (Table 3). Thereafter, the reaction mixture was 10-times-diluted and heated at 90°C for 5 min to promote the dissociation of cleaved fragments from the PCR products. After lyophilization followed by annealing, T4 DNA ligase was added to the mixture of the CMV promoter and GFP fragments, and the DNA fragments were ligated by treatment at 25°C for 3 h. As a control, DNA fragments with four bases overhang sticky ends, which were prepared by BsaI treatment (Table 3), were also treated with AgNPs using the same procedure as above. To test the effect of AgNPs on ligation efficiency, the BsaI-treated DNA fragments were ligated in the absence of AgNPs. As a result, the ligation efficiency of BsaI-treated DNA fragments decreased in the presence of AgNPs (Fig. 6b), probably because AgNPs bind to DNA fragments and mask the overhang sticky ends. Nevertheless, DNA fragments with 18 or 10 bases overhang sticky ends prepared by our cleavage technology showed much higher ligation efficiency than BsaI-treated DNA fragments (Fig. 6b and Supplementary Figs S33 and  S34), like the results shown in Fig. 5. Long sticky ends promote ligation by overcoming the disadvantages of the presence of AgNPs. This result suggests that rough purification provides a sufficiently high ligation efficiency, but that additional purification to remove AgNPs can further improve the ligation efficiency. 18 and 10 bases overhang sticky ends showed almost the same ligation efficiency, suggesting that the difficulty in the complete formation of long sticky ends, such as 18 bases, may cancel the benefit of improving ligation efficiency. Furthermore, we confirmed that the ligated products could produce the functional protein to emit GFP fluorescence (Fig. 6c), which was observed 24 h after transfection of HeLa cells with the ligation reaction mixture using Lipofectamine 3000. GFP was observed under all conditions; however, the number of GFP-positive cells seemed to reflect the ligation efficiency. These results suggest that, although there should be room for further improvement, our cleavage technology achieved an improvement in ligation efficiency by the formation of arbitrary-length sticky ends and the resulting construction of functional proteins.

**Table 3.** Primer sequences used in GFP construction

	Sequence (5′ → 3′)	Length	T_m_	GC%	Product length
CMV-fw	ACAATCTGCTCTGATGCCG	19-mer	56°C	53%	
CMV-rev-Ts18	AGTCGTATTsAATTTCGATsAAGCC	23-mer	52°C	35%	828 bp
CMV-rev-Ts10	AGTGAGTCGTsATTAATTTCGATAAG	25-mer	52°C	32%	832 bp
CMV-rev-BsaI	GGCTAC**GGTCTC**GTCGTATTAATTTCGATAAGCCAG				826 bp
GFP-fw-Ts18	ATCGAAATTsAATACGACTsCAC	21-mer	49°C	33%	1264 bp
GFP-fw-Ts10	ACGACTCACTsATAGGGAGAC	20-mer	53°C	50%	1252 bp
GFP-fw-BsaI	GGCTAC**GGTCTC**TACGACTCACTATAGGGAGAC				1252 bp
GFP-rev	GTGGGGATACCCCCTAGAG	19-mer	56°C	63%	

“Ts” represents 3′-phosphorothiolate. The sequence “GGTCTC” in the bold and underlined region represents the recognition site and digestion site of BsaI, respectively.

## Conclusion

We developed a novel oligonucleotide strand cleavage reaction induced by AgNP treatment. In addition, we found that PEGylation of AgNPs enhances their strand cleavage activity due to both improved oxidative stability and the dispersion property of AgNPs in aqueous media. This finding would be a promising example of AgNPs for biological tools and expands the range available for a new application of nanoparticle chemistry. By applying the AgNP-mediated DNA strand cleavage method, we successfully prepared DNA fragments with sticky ends, and T4 DNA ligase-mediated DNA fragment ligation was demonstrated to synthesize 848-bp and 2 074-bp DNAs. Our method was successfully applied to the construction of GFP-coding DNA and confirmed GFP expression in HeLa cells. We plan to apply the AgNP-induced strand cleavage method to the synthesis of genome-scale DNA construction with an over-kilobase pair length. In addition, this methodology is useful for the preparation of a randomly sequenced DNA library for screening highly potent DNA-based drugs and nanomaterials.

## Supplementary Material

gkag525_Supplemental_File

## Data Availability

The data underlying this article are available in the article and in its online supplementary material.
