# Supplementary material for: Silver nanoparticle-induced site-specific strand cleavage of chemically modified oligonucleotides for long-chain DNA assembly
Source: Nucleic Acids Res. 2026 Jun 11;54(11):gkag525. doi: 10.1093/nar/gkag525 (PMC13253348; doi:10.1093/nar/gkag525)
Supplement: gkag525_Supplemental_File [file gkag525_supplemental_file.pdf]

Silver Nanoparticle Induced Site-Specific Strand Cleavage Reaction of Chemically Modified Oligonucleotides for Long-Chain DNA Assembly

Table of Contents

1. **General Information and Instrument**

2. **Experimental Procedures**
   - 2-1. Synthesis of 3'-Thionucleoside Phosphoramidites
   - 2-2. Synthesis of Oligodeoxynucleotides
   - 2-3. DNA Strand Cleavage Experiment by Silver Nitrate
   - 2-4. DNA Strand Cleavage Reaction by Silver Nanoparticles
   - 2-5. Characterization of Properties of Silver Nanoparticles by UV Spectral Analysis
   - 2-6. Comparison of Recovery Yield of DNA Strand Cleavage Reaction Between Silver Nitrate and Silver Nanoparticles
   - 2-7. Characterization of AgNP by DLS Analysis
   - 2-8. Quantification of Silver Ion in AgNP Dispersions
   - 2-9. Application of Modified DNAs for PCR
   - 2-10. Preparation of Sticky End by DNA Strand Cleavage Reaction and Application for Long Chain DNA Construction
   - 2-11. GFP Coding DNA Construction
   - 2-12. Transfection of Ligated Sample to HeLa Cells

3. **Compounds Spectral Data**
4. **Optimization for Synthesis of 3'-Thiobenzoyl Compound**
5. **Synthesized DNA Characterization by HPLC, MALDI-TOF-MS, and dPAGE**
6. **Oligonucleotide Strand Cleavage Reaction by Silver Nitrate**
7. **Comparison of Silver Nanoparticle and Silver Nitrate for Oligonucleotide Strand Cleavage**
8. **Absorption Spectra of Silver Nanoparticles**
9. **Characterization of Silver Nanoparticles**
10. **Silver Nanoparticle Mediated Double Strand DNA Cleavage to Produce Overhang Structure**
11. **Application of 3'*S*-DNA for PCR**
12. **Design and Synthesis of Long-Chain DNA by Ligation**
13. **References**

1. General Information and Instrument

Standard abbreviations for the protecting groups are followed by the IUPAC-IUB Commission on Biochemical Nomenclature. All starting materials, reagents, and solvents of guaranteed grade, were purchased from FUJIFILM Wako Chemicals, Tokyo Chemicals, Sigma-Aldrich, or Kanto Chemicals and used without further purification. All reactions involving moisture sensitive reagents were performed under an argon atmosphere using oven dried glassware. Column chromatography was performed on silica gel (63–210 mesh) purchased from Kanto Chemicals. All solid-phase oligonucleotide synthesis reagents were purchased from Chem Genes or Glen Research. All solvent compositions are reported in volume % unless specified otherwise. Syntheses of oligonucleotides were performed on a DNA/RNA synthesizer NR-2A_7MX or NRs-4A_10R7NP (Nihon Techno Service). NMR spectra were taken on JOEL NMR-ECS 400 (400 MHz for ^{1}H NMR, 101 MHz for ^{13}C NMR, and 163 MHz for ^{31}P NMR), and JOEL NMR-ECS 600 (600 MHz for ^{1}H NMR, 151 MHz for ^{13}C NMR, and 243 MHz for ^{31}P NMR) instruments. The ^{1}H and ^{13}C NMR chemical shifts (δ) are reported in parts per million (ppm) relative to residual solvents: $CDCl_3$ (7.26 ppm for ^{1}H NMR, 77.16 ppm for ^{13}C NMR), DMSO-d_6 (2.50 ppm for ^{1}H NMR, 39.52 ppm for ^{13}C NMR), and CD_3CN (1.94 ppm for ^{1}H NMR, 1.32 and 118.26 ppm for ^{13}C NMR) (1). ESI-TOF mass spectra were obtained on a micro TOF-QII (Bruker Daltonics) instrument. MALDI-TOF mass spectra were obtained on an UltrafleXtreme (Bruker Daltonics) with 3-hydroxypicolinic acid as a matrix to detect the peaks of the synthesized DNAs. LCMS analyses of the synthesized oligonucleotides were performed by using Agilent 1290 Infinity II - 6530 LC/Q-TOF system and Waters ACQUITY H-Class PLUS_LBNW - Xevo G2-XS Qtof System_NQTW. UV spectra were taken on JASCO V-650 spectrometer. The measurement condition was as follows: response: medium, bandwidth: 1.0 nm, scan rate: 100 nm/min, data acquisition interval: 0.5 nm.

2. Experimental Procedures

2-1. Synthesis of 3'-*S*-Phosphorothioamidites

2-1-1. Synthesis of Thymidine Derivative (5)

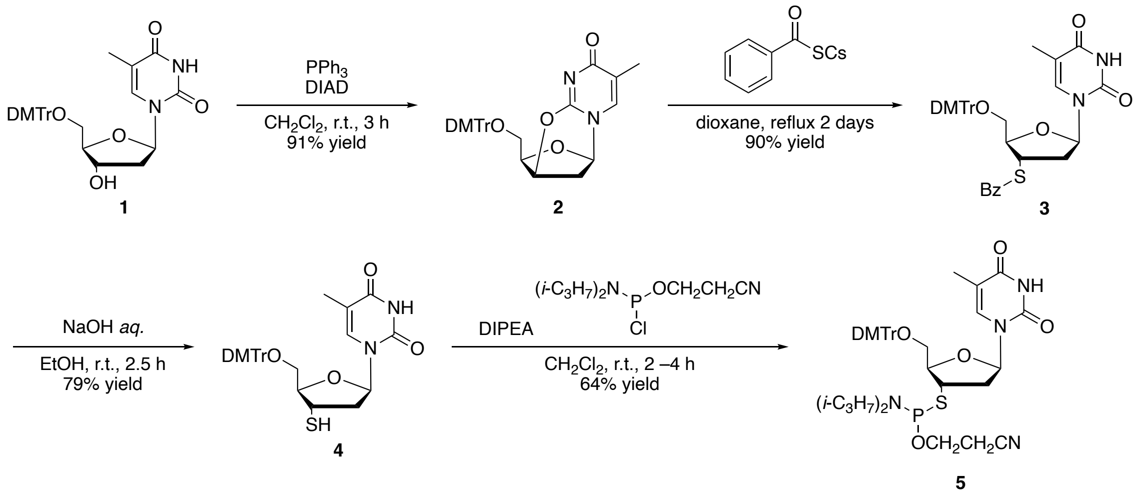

Scheme S1. Synthesis of 3'-Deoxy-3'-thiothymidine phosphoramidite (**5**).

compound 2: Commercially available 5'-*O*-DMTr-thymidine (**1**) (1.0 g, 1.8 mmol) and triphenyl phosphine (0.72 g, 2.8 mmol) were dissolved in dichloromethane (6.0 mL). Diisopropyl azodicarboxylate (0.54 g, 2.8 mmol) was added dropwise to the solution in an ice-bath. After

being stirred at room temperature for 3 hours, the reaction mixture was concentrated by using rotary evaporator. The residue was subjected to silica gel column chromatography eluted by 0/1 to 1/4 mixtures of methanol/ethyl acetate containing 1% triethylamine, to afford O2,3'-cyclothymidine (**2**) (0.89 g, 1.7 mmol, 94% yield) as white solid. ^1H NMR (400 MHz, DMSO-d_6) δ 7.59 (d, *J* = 1.3 Hz, 1H), 7.37–7.30 (m, 2H), 7.30–7.11 (m, 7H), 6.85–6.76 (m, 4H), 5.85 (d, *J* = 3.7 Hz, 1H), 5.27 (q, *J* = 2.2 Hz, 1H), 4.37 (ddd, *J* = 7.8, 4.8, 2.5 Hz, 1H), 3.68 (d, *J* = 2.5 Hz, 6H), 3.13–2.98 (m, 2H), 2.54 (dd, *J* = 12.8, 1.5 Hz, 1H), 2.44–2.37 (m, 1H), 1.73 (d, *J* = 1.1 Hz, 3H) ppm. All the spectral data of the product was consistent with literature (2),(3).

Compound 3: O2,3'-Cyclothymidine (**2**) (1.0 g, 1.9 mmol) and cesium thiobenzoate (1.8 g, 7.0 mmol) were suspended in 1,4-dioxane (40 mL). The mixture was stirred at 120 ºC for 1 day. Cesium thiobenzoate (1.8 g, 7.0 mmol) was added to the mixture and further stirred at 130 ºC for 1 day. The reaction mixture was cooled to room temperature and diluted with ethyl acetate. The mixture was washed 2 times with saturated aqueous $NaHCO_3$ solution. The organic layer was dried over Na_2SO_4 and concentrated by using rotary evaporator. The residue was subjected to silica gel column chromatography eluted by 0/1 to 1/60 mixtures of methanol/dichloromethane containing 1% triethylamine, to afford the 3'-deoxy-3'-thiobenzoyl thymidine derivative (**3**) (1.1 g, 1.7 mmol, 90% yield) as brown solid. ^1H NMR (600 MHz, $CDCl_3$) δ 7.93–7.86 (m, 2H), 7.71 (d, *J* = 1.4 Hz, 1H), 7.61–7.56 (m, 1H), 7.47–7.43 (m, 4H), 7.34–7.31 (m, 4H), 7.26 (dd, *J* = 8.4, 7.0 Hz, 2H), 7.22–7.17 (m, 1H), 6.82–6.77 (m, 4H), 6.30 (dd, *J* = 6.6, 5.2 Hz, 1H), 4.49 (q, *J* = 7.4 Hz, 1H), 4.14 (dt, *J* = 7.2, 2.7 Hz, 1H), 3.73 (d, *J* = 4.4 Hz, 6H), 3.52 (dd, *J* = 10.8, 2.4 Hz, 1H), 3.45 (dd, *J* = 10.8, 3.1 Hz, 1H), 2.75 (ddd, *J* = 13.8, 8.3, 5.2 Hz, 1H), 2.53–2.48 (m, 1H), 1.48 (d, *J* = 1.3 Hz, 3H) ppm. All the spectral data of the product was consistent with literature (2),(3).

Cesium thiobenzoate: $CO_3Cs_2 + 2PhCOSH \rightarrow 2PhCOSCs + CO_2 + H_2O$
Thiobenzoic acid was distilled at 80–90 ºC under vacuum following a literature procedure (4). The main distillate contained pure thiobenzoic acid, while the needle crystal residue consisted of benzoic acid. The distilled thiobenzoic acid (10.0 g, 72.4 mmol) was dissolved in dry acetonitrile (36.0 mL) and dry methanol (4.00 mL). Cesium carbonate (11.8 g, 36.2 mmol) was added to the solution in portions. After stirring at room temperature for 11 minutes, the mixture was concentrated and azeotroped three times with toluene. The residue was dried under reduced pressure to yield cesium thiobenzoate as a yellow powder (19.6 g, quant.). ^1H NMR (600 MHz, DMSO-d_6) δ 8.08 – 8.03 (m, 2H), 7.30 – 7.20 (m, 3H) ppm. ^{13}C NMR (151 MHz, DMSO-d_6) δ 207.25, 145.43, 128.74, 127.76, 126.68 ppm.

Compound 4: 3'-deoxy-3'-Thiobenzoyl thymidine derivative (**3**) (840 mg, 1.26 mmol) was dissolved in degassed ethanol (30.0 mL) and 10 M NaOH *aq.* (4.00 mL). The mixture was stirred at room temperature for 2 hours under argon bubbling. The reaction mixture was cooled in an ice-bath and then quenched by the addition of 1 M hydrochloric acid (40.0 mL). The resulting precipitate was collected by filtration and washed 3 times with water. The obtained solid was dissolved in dichloromethane, dried over Na_2SO_4, and concentrated by using rotary evaporator. The residue was subjected to silica gel column chromatography eluted by 1/1 to 1/3 mixtures of hexane/ethyl acetate containing 1% triethylamine, to afford the 3'-deoxy-3'-thiothymidine derivative (**4**) (560 mg, 0.995 mmol, 79% yield) as brown solid. ^1H NMR (600 MHz, $CDCl_3$) δ 7.68 (d, *J* = 1.4 Hz, 1H), 7.41 (dd, *J* = 7.5, 1.7 Hz, 2H), 7.34–7.20 (m, 7H), 6.83 (dd, *J* = 8.8, 2.0 Hz, 4H),

6.14 (dd, *J* = 7.1, 3.0 Hz, 1H), 4.10 (q, *J* = 7.1 Hz, 1H), 3.85 (dt, *J* = 8.8, 2.7 Hz, 1H), 3.77 (s, 6H), 3.63 – 3.56 (m, 2H), 3.39 (dd, *J* = 11.0, 2.9 Hz, 1H), 2.59 (ddd, *J* = 13.9, 7.6, 2.9 Hz, 1H), 2.35 (ddd, *J* = 13.9, 10.2, 7.1 Hz, 1H), 1.55 (d, *J* = 6.9 Hz, 1H), 1.48 (d, *J* = 1.2 Hz, 3H) ppm. All the spectral data of the product was consistent with literature (2),(3).

Compound 5: 3'-Deoxy-3'-Thiothymidine derivative (**4**) (360 mg, 0.640 mmol) was dissolved in dichloromethane (5.00 mL) and added *N*,*N*-diisopropylethylamine (360 mg, 0.640 mmol). The mixture was added 2-cyanoethyl diisopropylchlorophosphoramidite (185 μL, 0.830 mmol) and stirred at room temperature for 2 hours. The reaction mixture was diluted with dichloromethane and washed with saturated aqueous $NaHCO_3$ solution. The organic layer was dried over Na_2SO_4 and concentrated by using rotary evaporator. The residue was subjected to silica gel column chromatography eluted by 2/1 to 1/1.5 mixtures of hexane/ethyl acetate containing 1% triethylamine, to afford the 3'-phosphorothioamidite thymidine derivative (**5**) (312 mg, 0.410 mmol, 64% yield) as white solid. ^{1}H NMR (400 MHz, CD_3CN) δ 9.41 (s, 1H), 7.55–7.40 (m, 3H), 7.38–7.24 (m, 6H), 7.24–7.14 (m, 1H), 6.83 (dd, *J* = 8.7, 5.5 Hz, 4H), 6.18–5.99 (m, 1H), 4.10–3.93 (m, 2H), 3.86–3.75 (m, 1H), 3.73 (s, 6H), 3.71–3.44 (m, 4H), 3.44–3.28 (m, 1H), 2.66–2.53 (m, 2H), 2.53–2.39 (m, 1H), 1.95 (s, 1H), 1.55–1.42 (m, 3H), 1.31–1.05 (m, 9H), 1.03 (s, 1H), 1.01 (s, 1H) ppm. ^{31}P NMR (162 MHz, CD_3CN) δ 161.96, 158.44 ppm. All the spectral data of the product was consistent with literature (2),(3).

2-1-2. Synthesis of 2'-Deoxycytidine Derivative (10)

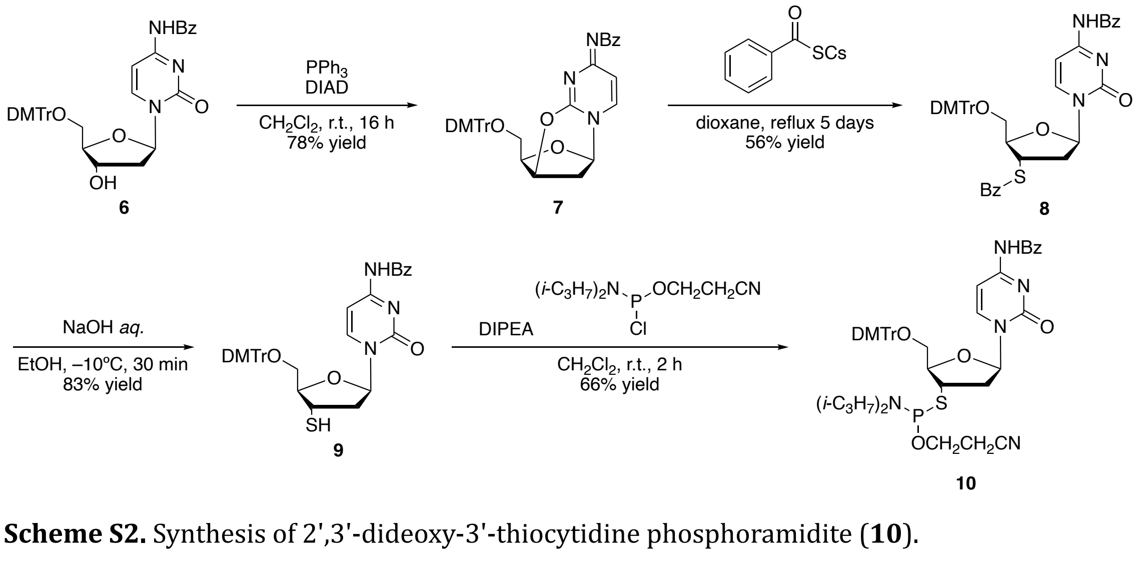

Scheme S2. Synthesis of 2',3'-dideoxy-3'-thiocytidine phosphoramidite (**10**).

Compound 7: *N*^{4}-Benzoyl-2'-deoxy 5'-*O*-DMTr-cytidine (**6**) (1.00 g, 1.57 mmol) was azeotroped 3 times with toluene (20.0 mL). The residue was added triphenyl phosphine (456 mg, 1.74 mmol) and dissolved in dichloromethane (6.00 mL). The mixture was added dropwise diisopropyl azodicarboxylate (456 mL, 1.74 mmol) in an ice-bath. After being stirred at room temperature for 16 hours, the reaction mixture was concentrated by using rotary evaporator. The residue was subjected to silica gel column chromatography eluted by 0/1 to 1/4 mixtures of methanol/ethyl acetate containing 1% triethylamine, to afford 2'-deoxy-*O*2,3'-cyclocytidine derivative (**7**) (780 mg, 1.26 mmol, 78% yield) as white solid. ^{1}H-NMR (600 MHz, DMSO-d_6) δ

7.81 (d, *J* = 7.1 Hz, 2H), 7.69 (d, *J* = 7.5 Hz, 1H), 7.51 (t, *J* = 7.3 Hz, 1H), 7.18–7.39 (m, 12H), 6.84 (dd, *J* = 9.0, 3.6 Hz, 4H), 6.42 (d, *J* = 7.5 Hz, 1H), 5.97 (d, *J* = 4.1 Hz, 1H), 5.33 (s, 1H), 4.40 (s, 1H), 3.70 (d, *J* = 2.4 Hz, 7H), 3.04-3.13 (m, 2H), 2.63 (d, *J* = 11.9 Hz, 1H) ppm. All the spectral data of the product was consistent with literature (2),(3).

Compound 8: 2'-deoxy-O2,3'-Cyclocytidine derivative (**7**) (2.8 g, 4.8 mmol) and cesium thiobenzoate (6.0 g, 24 mmol) were dissolved in 1,4-dioxane (60 mL). The mixture was stirred at room temperature for 5 hours. The reaction mixture was diluted with ethyl acetate (100 mL) and washed 2 times with saturated aqueous $NaHCO_3$ solution (100 mL), followed by brine (100 mL). The organic layer was dried over Na_2SO_4 and concentrated by using rotary evaporator. The residue was subjected to silica gel column chromatography eluted by 1/1 to 7/1 mixtures of ethyl acetate/hexane containing 1% triethylamine, to afford the 2',3'-dideoxy-3'-thiobenzoyl cytidine derivative (**8**) (2.0 g, 2.7 mmol, 56% yield) as brown solid. ^{1}H-NMR (600 MHz, DMSO-d_6) δ 11.29 (s, 1H), 8.55 (d, *J* = 7.5 Hz, 1H), 8.00 (d, *J* = 7.5 Hz, 2H), 7.88 (d, *J* = 7.1 Hz, 2H), 7.50–7.73 (m, 4H), 7.38 (d, *J* = 7.5 Hz, 2H), 7.14–7.27 (m, 9H), 6.82 (q, *J* = 4.5 Hz, 5H), 6.12 (d, *J* = 9.2 Hz, 1H), 4.40 (t, *J* = 9.0 Hz, 1H), 4.23 (s, 1H), 3.69 (d, *J* = 5.4 Hz, 1H), 3.66 (s, 6H), 2.65 (s, 2H), 2.36 (s, 1H) ppm. All the spectral data of the product was consistent with literature (2),(3).

Compound 10: 2',3'-dideoxy-3'-Thiobenzoyl cytidine derivative (**8**) (1.64 g, 2.20 mmol) was dissolved in degassed methanol (15 mL), THF (21 mL), and 0.5 M NaOH *aq.* (22 mL, NaOH: 11 mmol). The mixture was stirred at −10 ºC for 30 minutes under argon bubbling. The reaction mixture was quenched by the addition of 1 M KH_2PO_4 *aq.* (46.6 mL, 46.6 mmol). The mixture was diluted with ethyl acetate (100 mL) and washed 2 times with water (100 mL). The organic layer was dried over Na_2SO_4 and concentrated by using rotary evaporator. The residue was subjected to silica gel column chromatography eluted by 1/50 to 1/10 mixtures of methanol/dichloromethane containing 1% triethylamine, to afford the 2',3'-dideoxy-3'-thiocytidine derivative (**9**) (1.18 g, 1.82 mmol, 83% yield) as white solid. The obtained compound **9** (940 mg, 1.43 mmol) was dissolved in dichloromethane (15.2 mL). The mixture was added *N*,*N*-diisopropylethylamine (0.35 mL, 1.57 mmol) and 2-cyanoethyl diisopropylchlorophosphoramidite (0.350 mL, 1.57 mmol). After being stirred at room temperature for 2 hours, the reaction mixture was diluted with dichloromethane (20.0 mL) and washed with saturated aqueous $NaHCO_3$ solution (20.0 mL). The organic layer was dried over Na_2SO_4 and concentrated by using rotary evaporator. The residue was subjected to silica gel column chromatography eluted by 3/7 mixture of acetonitrile/dichloromethane containing 1% triethylamine, to afford the 3'-phosphorothioamidite 2'-deoxy-cytidine derivative (**10**) (1.00 g, 1.20 mmol, 66% yield) as white solid. ^{1}H-NMR (400 MHz, $CDCl_3$) δ 9.19 (s, 1H), 8.78 (s, 1H), 8.30 (d, *J* = 19.5 Hz, 1H), 8.02–8.05 (m, 2H), 7.51–7.63 (m, 3H), 7.37–7.42 (m, 2H), 7.27–7.31 (m, 5H), 7.18–7.25 (m, 3H), 6.77–6.81 (m, 4H), 6.42 (dd, *J* = 7.0, 2.5 Hz, 1H), 4.21–4.25 (m, 1H), 3.90 (t, *J* = 6.2 Hz, 1H), 3.79–3.85 (m, 1H), 3.77 (d, *J* = 4.3 Hz, 6H), 3.57–3.67 (m, 5H), 3.41–3.45 (m, 1H), 3.07–3.14 (m, 1H), 2.60 (t, *J* = 6.2 Hz, 1H), 2.44 (t, *J* = 6.3 Hz, 2H), 1.18–1.22 (m, 6H), 1.13–1.17 (m, 6H) ppm. ^{31}P-NMR (162 MHz, $CDCl_3$) δ 165.57, 161.68 ppm. All the spectral data of the product was consistent with literature (2),(3).

2-1-3. Synthesis of 2'-Deoxyadenosine Derivative (16)

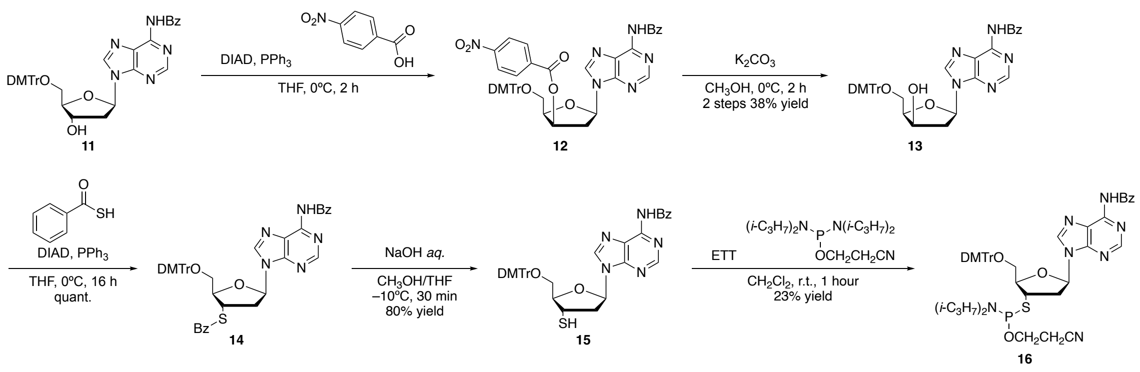

Scheme S3. Synthesis of 2',3'-dideoxy-3'-thioadenosine phosphoramidite (**16**).

Compound 13: *N*6-benzoyl-2'-deoxy-5'-*O*-DMTr-adenosine (**11**) (10.0 g, 15.2 mmol), 4-nitrobenzoic acid (5.08 g, 30.4 mmol), and triphenylphosphine (16.0 g, 61.0 mmol), were dissolved in THF (800 mL). Diisopropyl azodicarboxylate (11.9 mL, 61.0 mmol) was added dropwise to the solution in an ice-bath. After being stirred at 0 ºC for 2 hours, the reaction mixture was concentrated by using rotary evaporator. The residue was subjected to silica gel column chromatography eluted by 7/3 mixture of ethyl acetate/hexane, to afford the compound (**12**) (18.5 g) as white solid. The obtained compound **12** (10.0 g, 12.6 mmol) was added potassium carbonate (1.71 g, 49.6 mmol) and dissolved in methanol (125 mL). After being stirred for 2 hours in an ice-bath, the reaction mixture was concentrated by using rotary evaporator. The residue was dissolved in ethyl acetate (75 mL) and washed 2 times with water (75 mL). The organic layer was dried over Na_2SO_4 and concentrated by using rotary evaporator. The residue was subjected to silica gel column chromatography eluted by 1/1 mixture of ethyl acetate/hexane, to afford the 2',3'-dideoxy-3'-*β*-hydroxy-adenosine derivative (**13**) (1.40 g, 2.10 mmol, 2 steps 38% yield) as white solid. ^{1}H-NMR (400 MHz, DMSO-d_6) δ 11.18 (s, 1H), 8.75 (s, 1H), 8.47 (s, 1H), 8.03 (d, *J* = 7.6 Hz, 2H), 7.53–7.66 (m, 3H), 7.17–7.39 (m, 9H), 6.75–6.83 (m, 4H), 6.50 (d, *J* = 7.4 Hz, 1H), 5.48 (d, *J* = 4.3 Hz, 1H), 4.31 (d, *J* = 44.2 Hz, 2H), 3.66–3.74 (m, 6H), 3.37 (d, *J* = 8.1 Hz, 1H), 3.20 (d, *J* = 10.3 Hz, 1H), 2.78 (s, 1H), 2.38 (d, *J* = 14.6 Hz, 1H) ppm. All the spectral data of the product was consistent with literature (5).

Compound 16: To a solution of triphenylphosphine (2.4 g, 9.0 mmol) in THF (53 mL) was added diisopropyl azodicarboxylate (1.75 mL, 9.0 mmol) in an ice-bath. After being stirred for 30 minutes in an ice-bath, thiobenzoic acid (1.1 mL, 9.0 mmol) was added and further stirred at same temperature for 30 minutes. The mixture was added the compound **13** (2.0 g, 3.0 mmol) and stirred for 16 hours in an ice-bath. The reaction mixture was concentrated by using rotary evaporator. The residue was subjected to silica gel column chromatography eluted by 1/1 to 2/1 mixtures of ethyl acetate/hexane, to afford the compound **14** (2.3 g, 3.0 mmol, quant.) as white solid. The obtained compound **14** (2.3 g, 3.0 mmol) was dissolved in degassed methanol (42 mL), THF (28 mL), and 0.5 M NaOH *aq.* (18 mL, NaOH: 9.0 mmol). The mixture was stirred at –10 ºC for 30 minutes under argon bubbling. The reaction mixture was quenched by the addition of 1 M KH_2PO_4 *aq.* (38 mL, 19 mmol). The resulting precipitate was collected by filtration and washed 3 times with water (200 mL). The solid was dissolved in dichloromethane and purified by silica gel column chromatography eluted by 1/50 to 1/20 mixtures of methanol/dichloromethane

containing 1% triethylamine, to afford the compound (**15**) (1.62 g, 2.40 mmol, 80% yield) as white solid. The obtained compound **15** (1.5 g, 2.3 mmol) was added 5-ethylthio-1*H*-tetrazole (0.30 g, 2.3 mmol) and dissolved in dichloromethane (10 mL). Then, the mixture was added 2-cyanoethyl *N*,*N*,*N*',*N*'-tetraisopropylphosphordiamidite (1.1 mL, 3.5 mmol) and stirred at room temperature for 1 hour. The reaction mixture was diluted with dichloromethane (50 mL) and washed with saturated aqueous $NaHCO_3$ solution (50 mL). The organic layer was dried over Na_2SO_4 and concentrated by using rotary evaporator. The residue was subjected to silica gel column chromatography eluted by 2/1 to 8/1 mixtures of ethyl acetate/hexane, to afford the 2',3'-dideoxy-3'-thioadenosine phosphoramidite (**16**) (0.46 g, 0.54 mmol, 23% yield) as white solid. ^{1}H-NMR (400 MHz, $CDCl_3$) δ 9.19 (s, 1H), 8.78 (s, 1H), 8.30 (d, J = 19.5 Hz, 1H), 8.02–8.05 (m, 2H), 7.51–7.63 (m, 3H), 7.37–7.42 (m, 2H), 7.27-7.31 (m, 5H), 7.18–7.25 (m, 3H), 6.77–6.81 (m, 4H), 6.42 (dd, J = 7.0, 2.5 Hz, 1H), 4.21–4.25 (m, 1H), 3.90 (t, J = 6.2 Hz, 1H), 3.79–3.85 (m, 1H), 3.76–3.77 (m, 7H), 3.57–3.67 (m, 5H), 3.41–3.45 (m, 1H), 3.07–3.14 (m, 1H), 2.60 (t, J = 6.2 Hz, 1H), 1.18–1.22 (m, 6H), 1.13–1.17 (m, 6H) ppm. ^{31}P-NMR (162 MHz, $CDCl_3$) δ 165.22 ppm. All the spectral data of the product was consistent with literature (5).

2-1-4. Synthesis of 2'-Deoxyguanosine Derivative (23)

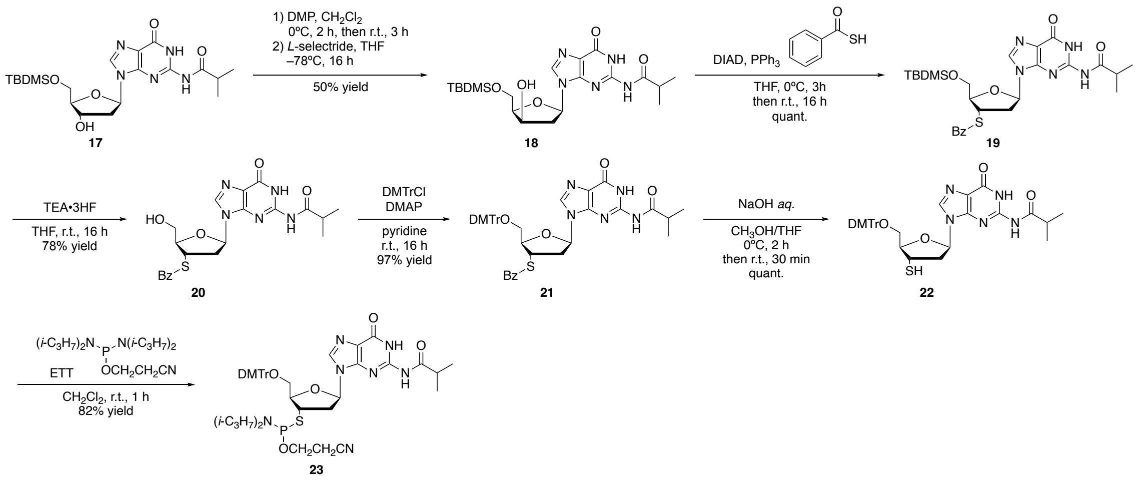

Scheme S4. Synthesis of 2',3'-dideoxy-3'-thioguanosine phosphoramidite (**23**).

Compound 18: N^2-Isobutyryl-5'-*O*-TBDMS-2'-deoxyguanosine (**17**) (7.3 g, 16 mmol) was dissolved in dichloromethane (370 mL). The solution was added Dess-Martin periodinane (34 g, 80 mmol) and sodium bicarbonate (21 g). The mixture was stirred for 2 hours in an ice-bath and then stirred at room temperature for 3 hours. After finished the reaction, the reaction mixture was added saturated aqueous $NaHCO_3$ solution (370 mL) and saturated aqueous $Na_2S_2O_3$ solution (370 mL). The mixture was extracted 2 times with dichloromethane (370 mL). The combined organic layer was dried over Na_2SO_4 and concentrated by using rotary evaporator. The residue containing 2'-keto derivative was dissolved in THF (260 mL) and stirred at –78 ºC for 30 minutes. Then, the mixture was added 1 M *L*-selectride/THF (40 mL, 40 mmol) and stirred at –78ºC for 16 hours. The reaction mixture was quenched by the addition of saturated aqueous NH_4Cl solution (50 mL). The mixture was extracted 3 times with ethyl acetate. The combined organic layer was dried over Na_2SO_4 and concentrated by using rotary evaporator. The residue was subjected to silica gel column chromatography eluted by 1/15 mixture of

methanol/dichloromethane, to afford the 2',3'-dideoxy-3'-β-hydroxy-guanosine (**18**) (3.6 g, 8.0 mmol, 50% yield) as white solid. ^{1}H-NMR (400 MHz, DMSO-d_6) δ 12.06 (s, 1H), 11.70 (s, 1H), 8.12–8.19 (m, 1H), 6.12 (dd, J = 8.2, 1.9 Hz, 1H), 5.35–5.39 (m, 1H), 4.29–4.37 (m, 1H), 3.92–4.11 (m, 2H), 3.73–3.79 (m, 1H), 2.65–2.81 (m, 2H), 2.21–2.32 (m, 1H), 1.08–1.15 (m, 6H), 0.83–0.86 (m, 9H), 0.16～–0.14 (m, 6H) ppm. All the spectral data of the product was consistent with literature (5).

Compound 20: Triphenylphosphine (11 g, 40 mmol) was dissolved in THF (140 mL) and added dropwise diisopropyl azodicarboxylate (7.8 mL, 40 mmol) in an ice-bath. After being stirred for 30 minutes in an ice-bath, thiobenzoic acid (4.7 mL, 40 mmol) was added and further stirred for 30 minutes at same temperature. Then, the compound **18** (3.0 g, 6.7 mmol) was added and stirred for 3 hours in an ice-bath. After being stirred at room temperature for 16 hours, the reaction mixture was concentrated by using rotary evaporator. The residue was purified by silica gel column chromatography eluted by 1/1 mixture of ethyl acetate/hexane, to afford the 2'-deoxy-3'-thiobenzoyl guanosine derivative (**19**) (25 g, 6.7 mmol, quant.) as white solid. The obtained compound **19** (25 g, 6.7 m,mol) was dissolved in THF (100 mL) and added triethylamine trihydrofluoride (16 mL, 100 mmol). After being stirred at room temperature for 16 hours, the reaction mixture was concentrated by using rotary evaporator. The residue was purified by silica gel column chromatography eluted by 1/15 mixture of methanol/dichloromethane. The product was recrystallized from dichloromethane to afford the compound **20** (2.4 g, 5.2 mmol, 78% yield) as white solid. ^{1}H-NMR (600 MHz, DMSO-d_6) δ 12.09 (s, 1H), 11.69 (s, 1H), 8.34 (d, J = 15.6 Hz, 1H), 7.93 (dd, J = 8.3, 0.8 Hz, 2H), 7.67–7.74 (m, 1H), 7.54–7.63 (m, 2H), 6.24 (t, J = 5.9 Hz, 1H), 5.20 (t, J = 5.4 Hz, 1H), 4.34 (q, J = 7.1 Hz, 1H), 4.09–4.14 (m, 1H), 3.60–3.74 (m, 2H), 3.01–3.05 (m, 1H), 2.72–2.79 (m, 1H), 2.58–2.63 (m, 1H), 1.11 (dd, J = 7.0, 2.2 Hz, 6H) ppm. All the spectral data of the product was consistent with literature (5).

Compound 21: The compound **20** (1.5 g, 3.3 mmol) and 4,4'-dimethoxytrityl chloride (2.2 g, 6.6 mmol) was dissolved in pyridine (30 mL). After being stirred at room temperature for 16 hours, the reaction mixture was concentrated by using rotary evaporator. The residue was dissolved in dichloromethane (50 mL) and washed with saturated aqueous $NaHCO_3$ solution (50 mL). The organic layer was dried over Na_2SO_4 and concentrated by using rotary evaporator. The residue was subjected to silica gel column chromatography eluted by 1/20 mixture of methanol/dichloromethane, to afford the compound **21** (2.4 g, 3.2 mmol, 97% yield) as white solid. ^{1}H-NMR (600 MHz, DMSO-d_6) δ 12.11 (s, 1H), 11.70 (d, J = 17.3 Hz, 1H), 8.17–8.26 (m, 1H), 7.89 (dd, J = 8.3, 1.2 Hz, 2H), 7.52–7.79 (m, 3H), 7.14–7.40 (m, 9H), 6.73–6.82 (m, 4H), 6.30 (q, J = 3.5 Hz, 1H), 4.45–4.57 (m, 1H), 4.20–4.23 (m, 1H), 3.61–3.79 (m, 6H), 3.22–3.31 (m, 2H), 3.17 (dq, J = 13.7, 3.9 Hz, 1H), 2.61–2.82 (m, 2H), 1.13 (dd, J = 6.8, 1.4 Hz, 6H) ppm. All the spectral data of the product was consistent with literature (5).

Compound 23: The compound **21** (2.0 g, 2.6 mmol) was dissolved in the mixed solvent of methanol/THF (3/2, v/v, 90 mL) and stirred for 30 minutes in an ice-bath. The mixture was added 0.5 M NaOH *aq.* (18 mL, 9.0 mmol) and stirred at 0 ºC for 2 hours then at room temperature for 30 minutes under argon bubbling. The reaction mixture was quenched by the addition of 1 M KH_2PO_4 *aq.* (33 mL, 33 mmol). Then, the mixture was diluted with ethyl acetate (300 mL) and washed with saturated aqueous $NaHCO_3$ solution (300 mL) and brine (300 mL).

The organic layer was dried over Na_2SO_4 and concentrated by using rotary evaporator to give the 2',3'-dideoxy-3'-thioguanosine derivative **22** (1.7 g) as white solid. The obtained compound **22** (1.7 g, 2.6 mmol, quant.) was added 5-ethylthio-1*H*-tetrazole (0.34 g, 2.6 mmol) and dissolved in dichloromethane (40 mL). Then, the mixture was added 2-cyanoethyl *N*,*N*,*N'*,*N'*-tetraisopropylphosphordiamidite (1.3 mL, 4.0 mmol) and stirred at room temperature for 1 hour. The reaction mixture was diluted with dichloromethane (300 mL) and washed 2 times with saturated aqueous $NaHCO_3$ solution (300 mL). The organic layer was dried over Na_2SO_4 and concentrated by using rotary evaporator. The residue was subjected to silica gel column chromatography eluted by 1/4 mixture of acetonitrile/dichloromethane, to afford the 2',3'-dideoxy-3'-thioguanosine phosphoramidite **23** (1.78 g, 2.08 mmol, 82% yield) as white solid. ^{1}H-NMR (600 MHz, $CDCl_3$) δ 12.00 (d, J = 10.2 Hz, 1H), 8.45 (d, J = 34.0 Hz, 1H), 7.83–7.87 (m, 1H), 7.41–7.43 (m, 2H), 7.29–7.31 (m, 4H), 7.17–7.24 (m, 3H), 6.73–6.82 (m, 4H), 6.14–6.17 (m, 1H), 4.19–4.25 (m, 1H), 3.87–3.55 (11H), 3.54–3.27 (2H), 3.00–3.12 (m, 1H), 2.32–2.68 (m, 4H), 1.09–1.21 (m, 18H) ppm. ^{31}P-NMR (243 MHz, $CDCl_3$) δ 161.11, 160.26 ppm. All the spectral data of the product was consistent with literature (5).

2-2. Synthesis of Oligodeoxynucleotides

2-2-1. Synthetic Procedure

DNAs with 3'-phosphorothiolate linkage were synthesized on a DNA/RNA synthesizer NR-2A_7MX or NRs-4A_10R7NP (Nihon Techno Service), using standard DNA phosphoramidites, CPG (Chemgenes; deoxy Guanosine (n-ibu) 3'-lcaa CPG 1000Å, Deoxy Cytidine (n-acetyl) 3'-lcaa CPG 1000Å, Thymidine 3'-lcaa CPG 100Å, or 6-FAM-glycerol support 500Å) and 3'-thionucleoside phosphoramidites (**5**). 70 mM DNA phosphoramidites and 150 mM 3'-thionucleoside phosphoramidites solutions in acetonitrile were prepared to use these for the DNA synthesis. Reagents for the synthesizer were used as follows: 3 w/v % trichloroacetic acid in dichloromethane for deblocking; 0.25 M 5-benzylthio-1*H*-tetrazole in acetonitrile (Wako) for coupling; a mixture of acetic anhydride/ tetrahydrofuran/ pyridine (1: 8: 1, Wako) and 10 (v/v)% 1-methylimidazole in tetrahydrofuran (Wako) for capping; 0.01 M iodine in 64% acetonitrile, 6% pyridine, 30% water for oxidation (Honeywell). The DNA synthesis was performed in the final DMT-ON mode. After the synthesis, 5'-DMTr-ON DNAs were cleaved from the support and deprotected using a 1: 1 mixture of 40% aqueous methylamine-28% ammonium hydroxide at 65 °C for 15 min. After cleavage and deprotection, the CPG was removed by filtration, and the filtrate was concentrated. The crude DNAs were purified by reversed-phase HPLC. The purification conditions were as follows: column, YMC Hydrosphere C18, 250 × 10 mm I.D., S-5 μm, 12 nm; Solution A, 50 mM triethylammonium acetate (pH 7.0) containing 5% acetonitrile; Solution B, acetonitrile; typical gradient, 0 to 60% Solution B over 20 min; column temperature, 50 °C; flow rate, 3 mL/min; detection wavelength, 260 nm. After the purification, the DMTr-ON-DNA was treated with 10% AcOH/water at room temperature for 1 hour to remove 5'-DMTr protecting group. The 5'-OH free DNA was further purified by reverse-phase HPLC. The DNA concentration was determined using the extinction coefficient calculated by the nearest neighbor method using the Oligoanalyzer software from Integrated DNA Technologies, based on the absorbance at 260 nm measured on a NanoDrop2000 spectrometer (Thermo Fisher Scientific).

2-2-2. List of Synthesized Oligodeoxynucleotides

Table S1. List of Synthesized 3'*S*-DNAs

name	Sequence (5'→3')	Mass (Da) Calcd.	Mass (Da) found	note
**3'*S*-DNA1**	TAAC**Ts**CACATTAATT GCGTT-FAM	6,649.579	6,649.353	3'-FAM-Modified Model Sequence to analyze the strand cleavage reaction
**3'*S*-DNA2**	AGGGGTGCCTAATG**Ts** GTGAGCTAACTCACA TTAATTGCGTT	12,686.362	12,686.542	Model Sequence to investigate the strand cleavage reaction and PCR
**3'*S*-DNA3**	AGCGCCA**Ts**TCGCCAT TCAGG	6,094.069	6,093.735	PCR primer (Rev for F1) in Figure 5
**3'*S*-DNA4**	ATGGCGC**Ts**TTGCCTG GTTTC	6,122.067	6,121.721	PCR primer (Fw for F2) in Figure 5
**3'*S*-DNA5**	AGTCGTAT**Ts**AATTTC GA**Ts**AAGCC	7,069.783	7,069.454	PCR primer (CMV-rev-Ts18) to generate 18 bases overhang in Figure 6
**3'*S*-DNA6**	AGTGAGTCG**Ts**ATTA ATTTCGATAAG	7,751.179	7,750.746	PCR primer (CMV-rev-Ts10) to generate 10 bases overhang in Figure 6
**3'*S*-DNA7**	ATCGAAAT**Ts**AATAC GAC**Ts**CAC	6,414.377	6,414.242	PCR primer (GFP-fw-Ts18) to generate 18 bases overhang in Figure 6
**3'*S*-DNA8**	ACGACTCAC**Ts**ATAGG GAGAC	6,151.132	6,151.115	PCR primer (GFP-fw-Ts10) to generate 10 bases overhang in Figure 6

Ts denotes 3'-phosphorothiolate

2-3. DNA Strand Cleavage Experiment by Silver Nitrate

Strand Cleavage by Silver Nitrate and dPAGE Analysis (Figure S15): 1 µL of 30 µM 3'*S*-modified DNA 20-mer (**3'*S*-DNA1**: 5'-TAA**Ts**CACATTAATTGCGTT-FAM-3') was mixed with 50 mM silver nitrate aqueous solution (29 µL) and incubated at room temperature for 5 to 40 minutes. When the specified time was reached, 5 µL of each reaction solution was taken and the reaction was quenched by adding 60 mM aqueous ethanethiol solution (5 µL). At this time, the formation of a white precipitate derived from DNA-silver ion complex was observed. 2x loading buffer (10 µL) was added to this mixture (10 µL). After heat treatment at 95ºC for 5 minutes, the samples were analyzed by 15% denaturing acrylamide gel electrophoresis (containing 7.5 M urea, 10 x 12 cm, 30 mA, 20 minutes, 6 µL Loading). A gel electrophoresis image was obtained using a gel image analyzer (BioRad) using FAM-derived fluorescence detection.

Strand Cleavage by Silver Nitrate and MALDI-TOF-MS Analysis of the Products (Figure S16): 3 µL of 110 µM 3'*S*-modified DNA 41-mer (**3'*S*-DNA2**: 5'-AGGGGTGCCTAATG**Ts**GTGAGCTAACTCACATTAATTGCGTT-3') was mixed with 50 mM silver nitrate aqueous solution (67 µL) and incubated at room temperature for 22 hours. Silver nitrate was removed from the reaction solution using an ultrafiltration centrifugal filter (Amicon Ultra 3K, manufactured by Merck), and molecules with a molecular weight smaller than 3K were cut

off according to the manufacturer's recommended protocol. The solution remaining after ultrafiltration was collected and subjected to MALDI-TOF-MS analysis. MALDI-TOF-MS analysis was performed using 3-hydroxypicolinic acid as a matrix in the linear negative mode of UltraFleXtreme (Bruker).

Quantification of the Amount of Cleavage Products Recovered After DNA Strand Cleavage by Silver Nitrate Treatment (Figure S17): 10 μL (1.10 nmol) of 110 μM 3'*S*-modified DNA 41-mer (**3'*S*-DNA2**: 5'-AGGGGTGCCTAATG**Ts**GTGAGCTAACTCACATTAATTGCGTT-3') was mixed with 50 mM silver nitrate aqueous solution (90 μL) and incubated at room temperature for 1 day. 20 μL (220 pmol) of each reaction solution was taken and the reaction was quenched by adding 60 mM dithiothreitol (DTT) aqueous solution (20 μL) or 60 mM ethanethiol (EtSH) aqueous solution (20 μL). At this time, the formation of a white precipitate derived from DNA-silver ion complex was observed. This mixture was diluted with ultra-deionized water (160 μL) and incubated for 20 min at room temperature. Subsequently, the precipitate derived from DNA-silver ion complex was removed by centrifugation (15,000 rpm, 15 minutes). The supernatant was collected and concentrated using an ultrafiltration centrifugal filter (Amicon Ultra 3K, manufactured by Merck) according to the manufacturer's recommended protocol. The recovered amount of the product was calculated by quantifying the cleavage product by measuring the absorbance of the concentrated sample solution at 260 nm derived from the nucleotides using NanoDrop. In calculating the amount of cleavage product, the sum of the molar extinction coefficients at 260 nm of both cleavage products, ε_{260} = 396,900 L•mol^{-1}•cm^{-1}, or the 3' side cleavage product (5'-pGTGAGCTAACTCACATTAATTGCGTT-3'), ε_{260} = 250,000 L•mol^{-1}•cm^{-1}, were used based on Lambert-Beer law. 5 μL of the concentrated sample solutions were mixed with 2x loading buffer (5 μL). After treatment at 95 ºC for 10 minutes, the samples were analyzed by 15% denaturing acrylamide gel electrophoresis (containing 7.5 M urea, 10 x 12 cm, 30 mA, 20 minutes, 6 μL loading). The gel after electrophoresis was immersed in 1x SYBR Green II aqueous solution and shaken for 20 minutes to stain the DNA band present in the gel. By detecting fluorescence derived from SYBR Green II bound to DNA, a gel electrophoresis image was obtained using a gel image analyzer (BioRad).

2-4. DNA Strand Cleavage Reaction by Silver Nanoparticles

Nanoparticle Size Dependency for the DNA Strand Cleavage: 10 μL of 3 μM 3'*S*-modified DNA (**3'*S*-DNA1**: 5'-TAAC**Ts**CACATTAATTGCGTT-FAM-3') was added to 50 μL of silver nanoparticle dispersion (10, 20 or 100 nm, 0.02 mg/mL), and the mixture was incubated at 37 ºC for 31 hours. 20 μL of the reaction solution was taken and added 20 μL of 2x loading buffer to the solution. After heat treatment at 95 ºC for 5 minutes, it was analyzed by 15% denaturing acrylamide electrophoresis (containing 7.5 M urea, 10 x 12 cm, 30 mA, 20 minutes, 6 μL loading). A gel electrophoresis image was obtained by gel image analyzer (BioRad) from FAM-derived fluorescence.

Reaction Time/Temperature Dependency for the DNA Strand Cleavage by Silver Nanoparticle Treatment: 10 μL of 3 μM 3'*S*-modified DNA (**3'*S*-DNA1**: 5'-TAAC**Ts**CACATTAATTGCGTT-FAM-3') was added to 50 μL of silver nanoparticle dispersion (10 nm, 0.02 mg/mL) and incubated at 70 ºC or 95 ºC. 30 μL of the reaction solution was taken and added 30 μL of 2x loading buffer to the reaction mixture. After heat treatment at 95 ºC for 5 minutes, it was analyzed by 15% denaturing acrylamide electrophoresis (containing 7.5 M urea, 10 x 12 cm, 30 mA, 20 minutes, 6 μL loading). A gel electrophoresis image was obtained by gel image analyzer (BioRad) from FAM-derived fluorescence.

DNA Strand Cleavage by PEGylated Silver Nanoparticle: 9 μL of 3.4 μM 3'*S*-modified DNA (**3'*S*-DNA1**: 5'-TAAC**Ts**CACATTAATTGCGTT-FAM-3') was added to 51 μL of surface PEG-modified silver nanoparticle dispersion (10 nm), and the mixture was incubated at the specified time and temperature. The surface PEG-modified silver nanoparticle dispersion was prepared by adding 1 μL aqueous solution of polyethylene glycol with an average molecular weight of 5,000 modified having terminal thiols, *O*-[2-(3-mercaptopropionylamino)ethyl]-*O'*-methylpolyethylene glycol, (23.9 g/L) to 50 μL of a commercially available silver nanoparticle dispersion (Sigma-Aldrich, 10 nm, 0.02 mg/mL). 20 μL of the reaction solution was taken and mixed with 20 μL of 2x loading buffer. After heat treatment at 95 ºC for 5 minutes, it was analyzed by 15% denaturing acrylamide electrophoresis (containing 7.5 M urea, 10 x 12 cm, 30 mA, 20 minutes, 6 μL loading). A gel electrophoresis image was obtained by gel image analyzer (BioRad) from FAM-derived fluorescence.

Preparation of 3'-Overhang Sticky End by Strand Cleavage of Double Strand DNA: 3'*S*-modified DNA (**3'*S*-DNA1**: 5'-TAAC**Ts**CACATTAATTGCGTT-FAM-3') and complementary DNA (5'-AACGCAATTAATGTGAGTTA-3') were mixed to 3 μM each and treated at 95 ºC for 3 minutes. After that, the mixture was placed on an ice-bath, and the annealing was performed by cooling for 10 minutes or more. After annealing, 9 μL of the solution was taken and added to 51 μL of surface PEG-modified silver nanoparticle dispersion (10 nm). The mixture was incubated at 50 ºC for the specified time. 30 μL of the reaction solution was collected and mixed with 30 μL of 2x loading buffer. After heat treatment at 95 ºC for 5 minutes, it was analyzed by 15% denaturing acrylamide electrophoresis (containing 7.5 M urea, 10 x 12 cm, 30 mA, 20 minutes, 6 μL loading). A gel electrophoresis image was obtained by gel image analyzer (BioRad) from FAM-derived fluorescence.

2-5. Characterization of Properties of Silver Nanoparticles by UV Spectral Analysis

Demonstration of Precipitation of Silver Nanoparticles by Centrifugation (Figure S20): 700 μL of a dispersion of silver nanoparticles with particle sizes of 10, 20, and 100 nm (Sigma-Aldrich, 0.02 mg/mL sodium citrate aqueous solution) was diluted with ultra-deionized water (700 μL), and then absorption spectra were measured using a quartz cell (optical path length: 1 cm, optical path width: 1 cm). After measuring the absorption spectrum, the silver nanoparticle dispersion diluted with ultra-deionized water (700 μL) was transferred to an Eppendorf tube, centrifuged at 15,000 rpm for 1 hour to precipitate the nanoparticles, and the supernatant was collected and transferred to the quartz cell again. After transferring to the cell (optical path length: 1 cm, optical path width: 1 cm), the absorption spectrum was measured.

Evaluation of Dispersion Property of PEGylated Silver Nanoparticle (Figure S21): The absorption spectra of 1) silver nanoparticle dispersion without surface modification, 2) Silver nanoparticle dispersion without surface modification in the presence of buffer/salts, 3) PEGylated silver nanoparticle dispersion, 4) PEGylated silver nanoparticle dispersion in the presence of buffer/salts, were measured by using a quartz cell (light path length: 1 cm, light path width: 1 cm). The solution preparation was conducted as follow.

1) Silver nanoparticle dispersion without surface modification: 350 μL of a dispersion of silver nanoparticles with a particle size of 20 nm (Sigma-Aldrich, 0.02 mg/mL sodium citrate aqueous solution) was diluted with ultra-deionized water (1050 μL).
2) Silver nanoparticle dispersion without surface modification in the presence of buffer/salts: 350 μL of a dispersion of silver nanoparticles with a particle size of 20 nm (Sigma-Aldrich, 0.02 mg/mL sodium citrate aqueous solution) was diluted with ultra-deionized water (630

μL), and added 100 mM Tris-HCl buffer (pH 8.3) (140 μL), 500 mM potassium chloride aqueous solution (140 μL), and 15 mM magnesium chloride aqueous solution (140 μL).

3) PEGylated silver nanoparticle dispersion: 350 μL of a dispersion of silver nanoparticles with a particle size of 20 nm (Sigma-Aldrich, 0.02 mg/mL sodium citrate aqueous solution) and 140 μL of 23.9 g/L aqueous solution of *O*-[2-(3-mercaptopropionylamino)ethyl]-*O*'-methylpolyethylene glycol (average molecular weight 5,000, Sigma-Aldrich) were mixed and diluted with ultra-deionized water (910 μL).

4) PEGylated silver nanoparticle dispersion in the presence of buffer/salts: 350 μL of a dispersion of silver nanoparticles with a particle size of 20 nm (Sigma-Aldrich, 0.02 mg/mL sodium citrate aqueous solution) and 140 μL of 23.9 g/L aqueous solution of *O*-[2-(3-mercaptopropionylamino)ethyl]-*O*'-methylpolyethylene glycol (average molecular weight 5,000, Sigma-Aldrich) were mixed and diluted with ultra-deionized water (490 μL). To the mixture, 100 mM Tris-HCl buffer (pH 8.3) (140 μL), 500 mM potassium chloride aqueous solution (140 μL), 15 mM magnesium chloride aqueous solution (140 μL) were added.

Evaluation of Dispersion Property of Silver Nanoparticle In the presence of PEG4000 (Figure S22): The absorption spectra of 1) silver nanoparticle dispersion with PEG4000, 2) silver nanoparticle dispersion with PEG4000 in the presence of buffer/salts, were measured by using a quartz cell (light path length: 1 cm, light path width: 1 cm). The solution preparation was conducted as follow.

1) Silver nanoparticle dispersion with PEG4000: 350 μL of a dispersion of silver nanoparticles with a particle size of 20 nm (Sigma-Aldrich, 0.02 mg/mL sodium citrate aqueous solution) was diluted with ultra-deionized water (910 μL), and added 140 μL of 15.8 g/L aqueous solution of PEG4000 (polyethylene glycol 4,000, average molecular weight 2700-3300, Fujifilm-Wako Pure Chemical Industries, Ltd.).

2) Silver nanoparticle dispersion with PEG4000 in the presence of buffer/salts: 350 μL of a dispersion of silver nanoparticles with a particle size of 20 nm (Sigma-Aldrich, 0.02 mg/mL sodium citrate aqueous solution) was diluted with ultra-deionized water (490 μL), and added 140 μL of 15.8 g/L aqueous solution of PEG4000 (polyethylene glycol 4,000, average molecular weight 2700-3300, Fujifilm-Wako Pure Chemical Industries, Ltd.), 100 mM Tris-HCl buffer (pH 8.3) (140 μL), 500 mM potassium chloride aqueous solution (140 μL), and 15 mM magnesium chloride aqueous solution (140 μL).

2-6. Comparison of Recovery Yield of DNA Strand Cleavage Reaction Between Silver Nitrate and Silver Nanoparticles

Silver Nitrate Treatment: 2 μL of 122 μM 3'*S*-modified DNA 20-mer (**3'*S*-DNA1**: 5'-TAA**Ts**CACATTAATTGCGTT-FAM-3') was mixed with 50 mM silver nitrate aqueous solution (38 μL) and incubated at room temperature for 1.5 hours. When the predetermined time was reached, the reaction was stopped by adding 60 mM DTT aqueous solution (40 μL) to the reaction solution (40 μL). At this time, the formation of a white precipitate derived from DTT-silver ion complex was observed. This mixture (80 μL) was diluted with ultra-deionized water (20 μL), mixed well, and the precipitate was removed by centrifugation at 15,000 rpm for 1 hour. The supernatant was collected and concentrated using an ultrafiltration centrifugal filter (Amicon Ultra 3K, manufactured by Merck) according to the manufacturer's recommended protocol. The recovered amount of the product was calculated by quantifying the cleavage product by measuring the absorbance of the concentrated sample solution at 260 nm derived from the nucleotides using NanoDrop. The concentrated sample solution was diluted with ultra-

deionized water to prepare a 0.50 μM aqueous solution. 5 μL of 0.50 μM cleavage product solution was mixed with 2x loading buffer (5 μL). After treatment at 95 ºC for 5 minutes, the samples were analyzed by 15% denaturing acrylamide gel electrophoresis (containing 7.5 M urea, 10 x 12 cm, 30 mA, 20 minutes, 5 μL application). A gel electrophoresis image was obtained by gel image analyzer (BioRad) from FAM-derived fluorescence.

Silver Nanoparticle Treatment: 2 μL of 122 μM 3'*S*-modified DNA 20-mer (**3'*S*-DNA1**: 5'-TAA**Ts**CACATTAATTGCGTT-FAM-3') was added to a silver nanoparticle dispersion with a particle size of 100 nm (Sigma-Aldrich, 0.02 mg/mL sodium citrate aqueous solution, 98 μL). The mixture was incubated at 90 ºC for 25 hours. The silver nanoparticle was removed as a precipitate by centrifuging the reaction solution at 15,000 rpm for 1 hour. The supernatant was collected and concentrated using an ultrafiltration centrifugal filter (Amicon Ultra 3K, manufactured by Merck) according to the manufacturer's recommended protocol. The recovered amount of the product was calculated by quantifying the cleavage product by measuring the absorbance of the concentrated sample solution at 260 nm derived from the nucleotides using NanoDrop. The concentrated sample solution was diluted with ultra-deionized water to prepare a 0.50 μM aqueous solution. 5 μL of 0.50 μM cleavage product solution was mixed with 2x loading buffer (5 μL). After treatment at 95 ºC for 5 minutes, the sample was analyzed by 15% denaturing acrylamide gel electrophoresis (containing 7.5 M urea, 10 x 12 cm, 30 mA, 20 minutes, 5 μL application). Gel images were obtained by gel image analyzer (BioRad) from FAM-derived fluorescence.

2-7. Characterization of AgNP by DLS Analysis

Dynamic light scattering (DLS) measurements were performed using a Zetasizer Pro ZSU3200 (Malvern Panalytical). A ZEN0040 cell was used for particle size and polydispersity index (PDI) measurements, and a DTS1070 cell was used for ζ-potential measurements. All measurements were conducted in colloidal silver mode at 25 °C with water as the dispersant. For ζ-potential measurements, 100 μL of the sample for particle size and PDI analysis was diluted with 600 μL of Milli-Q water. The compositions and preparation procedures for each sample are described below:

1) **AgNP (0.017 mg/mL):** 100 μL of AgNP dispersion (0.02 mg/mL in aqueous buffer containing sodium citrate) was diluted with 20 μL of water.
2) **PEGylated AgNP (0.017 mg/mL):** 100 μL of AgNP dispersion (0.02 mg/mL in aqueous buffer containing sodium citrate) was mixed with 2 μL of O-[2-(3-mercaptopropionylamino)ethyl]-O′-methylpolyethylene glycol (average molecular weight 5,000; 23.9 g/L aqueous solution) and diluted with 18 μL of water.
3) **AgNP–DNA cleavage (0.017 mg/mL):** 100 μL of AgNP dispersion (0.02 mg/mL in aqueous buffer containing sodium citrate) was diluted with 19 μL of water, followed by addition of 1 μL of 122 μM 3′S-DNA. The sample was heated at 95 °C for 1 h (10 nm AgNP) or 2 h (20 nm AgNP).
4) **PEGylated AgNP–DNA cleavage (0.017 mg/mL):** 100 μL of AgNP dispersion (0.02 mg/mL in aqueous buffer containing sodium citrate) was mixed with 2 μL of O-[2-(3-mercaptopropionylamino)ethyl]-O′-methylpolyethylene glycol (average molecular weight 5,000; 23.9 g/L aqueous solution) and diluted with 17 μL of water, followed by addition of 1 μL of 122 μM 3′S-DNA. The sample was heated at 50 °C for 1 h (10 nm AgNP) or 2 h (20 nm AgNP).

2-8. Quantification of Silver Ion in AgNP Dispersions

Calibration standards containing 0, 9.33, 28.0, 46.7, 93.3, 280, and 467 μM silver nitrate (corresponding to 0, 1, 3, 5, 10, 30, and 50 ppm Ag^+) and 667 μM 3,3',5,5'-tetramethylbenzidine (TMB) in 133 mM NaOAc–AcOH buffer (pH 4) were prepared to construct a calibration curve for colorimetric quantification of silver ions. Samples were incubated at room temperature for 15 min and analyzed using a UV-1900i UV–vis spectrophotometer (Shimadzu). Each experiment was performed independently in triplicate. The absorbance of oxidized TMB (oxTMB) at 655 nm was plotted against silver ion concentration, and a calibration curve was obtained by linear fitting (6).

A mixture of 10 mM TMB in ethanol (100 μL), 1 M NaOAc–AcOH buffer (pH 4, 200 μL), AgNP dispersion (10 or 20 nm, 750 μL), and Milli-Q water (450 μL) was prepared and analyzed by UV–vis spectroscopy. Silver ion concentrations were calculated from the absorbance at 655 nm (oxTMB) using the calibration equation: $y = 0.0091474x - 0.016989$.

2-9. Application of Modified DNAs for PCR

PCR Using 3'*S*-DNA as Template (Figure S28): 110 μM 3'*S*-DNA template (**3'*S*-DNA2**: 0.37 μL) and 15 μM primer DNA: 5'-FAM-AACGCAATTAATGTGAGTTAGC-3' (1.34 μL) were mixed and added to the mixtures of dNTP, various polymerases (KOD-Plus-Neo, PrimeSTAR HS, Phusion High Fidelity, Q5 High Fidelity, Deep Vent, Taq DNA polymerase) and polymerase buffers, to make a total volume of 20 μL. Denaturation (95 ºC, 1 minute), annealing (50 ºC, 30 seconds), and chain extension (72 ºC, 30 minutes) were performed, and the reaction solution was diluted with 2x loading buffer (20 μL). It was analyzed by 20% denaturing gel electrophoresis (containing 7.5 M urea, 20 x 22 cm, 20 W, 2 h). The gel was stained using 1x SYBR Green II solution by shaking for 30 minutes, and the gel image was obtained using a gel image analyzer (BioRad).

PCR Using 3'*S*-DNA Primers: 20 μM PS-Forward primer (1.25 μL), 20 μM 3'*S*-Reverse primer (1.25 μL), 10 ng/μL template DNA (2.5 μL), 2 mM dNTP mixture (5 μL), 25 mM magnesium sulfate (3 μL), 10x PCR Buffer for KOD-Plus-Neo (5 μL), and ultra-deionized water (31 μL), were mixed and added to 1 U/μL KOD-Plus-Neo (1 μL). By using a thermal cycler (BioRad), the sample was heated to 95 ºC for 2 minutes. Subsequently, DNA was amplified by performing 30 cycles of denaturation (95 ºC, 15 seconds), annealing (55 ºC, 15 seconds), and chain extension (68 ºC, 30 seconds). The PCR product was purified using a wizard column (Promega) according to the manufacturer's recommended protocol to obtain amplified 3'*S*-modified DNA.

2-10. Preparation of Sticky End by DNA Strand Cleavage Reaction and Application for Long Chain DNA Construction

Preparation of Sticky End DNA Fragments by Silver Nanoparticles: 4.5 μL of 7.5 nM PCR product 3'*S*-modified DNA was added to PEGylated silver nanoparticle dispersion which was prepared by mixing 10 nm silver nanoparticles dispersion (Sigma-Aldrich, 200 μg/mL in sodium citrate buffer, 25 μL) and *O*-[2-(3-mercaptopropionylamino)ethyl]-*O*'-methylpolyethylene glycol (Sigma-Aldrich, average molecular weight 5,000, 23.9 g/L aqueous solution, 0.5 μL). The mixture was treated at 50 ºC for 2 to 4 hours to cleave 3'-phosphorothiolate bonds and prepare sticky ends.

DNA Ligase-Mediated Ligation of DNA Fragments: 7.5 nM silver nanoparticle treated product DNA_F1 (3.6 μL), 7.5 nM silver nanoparticle treated product DNA_F2 (3.6 μL), 10x T4 DNA Ligase Reaction Buffer (New England BioLabs, 0.90 μL), and ultra-deionized water (0.45 μL) were mixed. To this mixture, T4 DNA ligase (New England BioLabs, 2000 U/μL, 0.45 μL) was added

and incubated at 25 ºC for 3 hours. 1 μL of 10x loading buffer was added to the reaction solution (9 μL) and analyzed by 1% agarose gel electrophoresis (running buffer: 1x TBE, 100 V, 30 minutes). The gel was stained using a 10,000x SYBR green I solution by shaking for 30 minutes, and a gel image was obtained using a gel image analyzer (manufactured by BioRad).

2-11. GFP Coding DNA Construction

DNA fragments encoding CMV promoter or GFP coding sequence were amplified by PCR using synthesized 3'*S*-modified primers with pcDNA6.2 emGFP plasmid as template. Used primers pairs producing PCR products with 10 or 18 nt overhang sticky ends were listed in Table 3. PCR reaction mixture contains 0.3 μM primers, 1 ng/μL template plasmid, 0.2 mM dNTPs, 1.5 mM $MgSO_4$, 1× reaction buffer and 0.02 U/μL KOD-Plus-Neo polymerase. Product length of CMV-promoter fragment and GFP-coding fragment are 830 and 1,260 bp, respectively.

The obtained PCR products with these primers were roughly purified by Wizard® SV Gel and PCR Clean-Up system (Promega) according to the manufacturers protocol and treated with PEGylated silver nanoparticles (10 nm) at 50 ºC for 4 hours. Thereafter, reaction mixture was 10-times-diluted and heated at 90 ºC for 5 minutes to promote the dissociation of cleaved fragments from PCR products. After concentration by lyophilization, mixed solutions of CMV promoter and GFP-coding fragments (around 400 fmol each) were heated with 90 °C for 3 minutes and cooled slowly to room temperature for promoting annealing. Then, 50 U/μL of NEB T4 ligase was added to the mixture and incubated them at 25 ºC for 3 hours. As a control with short overhang sticky ends, DNA fragments with 4 bases overhang were prepared by BsaI treatment against rough purified PCR products prepared as described above (see primer sequence in Table 3). PCR condition and purification step were same as described above. For digestion, BsaI reaction mixture containing 200-300 ng/μL PCR product, 1× CutSmart buffer and 0.6 U/μL BsaI-HFv2 (NEB) was incubated at 37ºC for 1 hour. After incubation, digested fragments purified by Wizard® SV Gel and PCR Clean-Up system (Promega) followed by ligation; ligation protocol is same as AgNP-treated fragments. To see whether silver nanoparticles affect to ligation efficiency or not, BsaI-treated DNA fragments were ligated in the presence and absence of silver nanoparticles for comparison.

2-12. Transfection of Ligated Sample to HeLa Cells

To confirm the function of protein synthesized from ligated products, these ligated solutions were transfected to HeLa cells after inactivation of T4 ligase by heat treatment at 65 ºC for 10 minutes. HeLa cells (1.0×10^4) suspended in D-MEM medium containing FBS were seeded on 96-well plate and incubated at 37°C under 5% CO_2 atmosphere for 24 hours. After cells became stable, ligated solution were transfected to HeLa cells using lipofectamine 3000 (Thermo Fisher Scientific Inc.) as manufacturer described. Briefly, 5 μL of heat-inactivated ligated solution was mixed with 0.3 μL Lipo3000, 1 μL P3000 and OPTI-MEM up to 10 μL followed by incubation at room temperature for 15 minutes, then added to HeLa cells. After 6 hours incubation at 37°C under 5% CO_2 atmosphere, D-MEM containing lipofectamine solution was replaced with fresh D-MEM. When changing solution in all subsequent steps, two- or three- times wash with PBS was performed. After additional 18 hours incubation (24 hours from transfection), D-MEM was removed and replaced with 4% PFA in PBS to fix cells. After 25 minutes incubation at room temperature, PFA was removed and replaced with PBS for microscopic observation by benchtop fluorescence microscopy, BZ-X810 (Keyence).

3. Compounds Spectral Data

^{1}H NMR Spectrum of Compound **2** (400 MHz, DMSO-d_6)

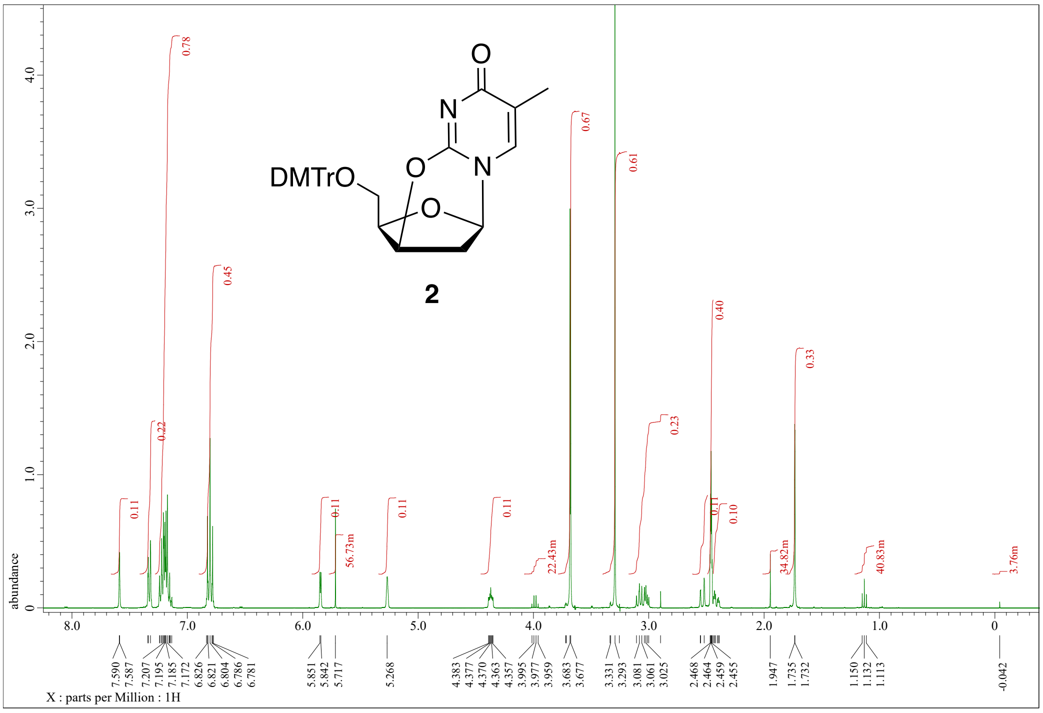

^{1}H NMR Spectrum of Compound **3** (600 MHz, $CDCl_3$)

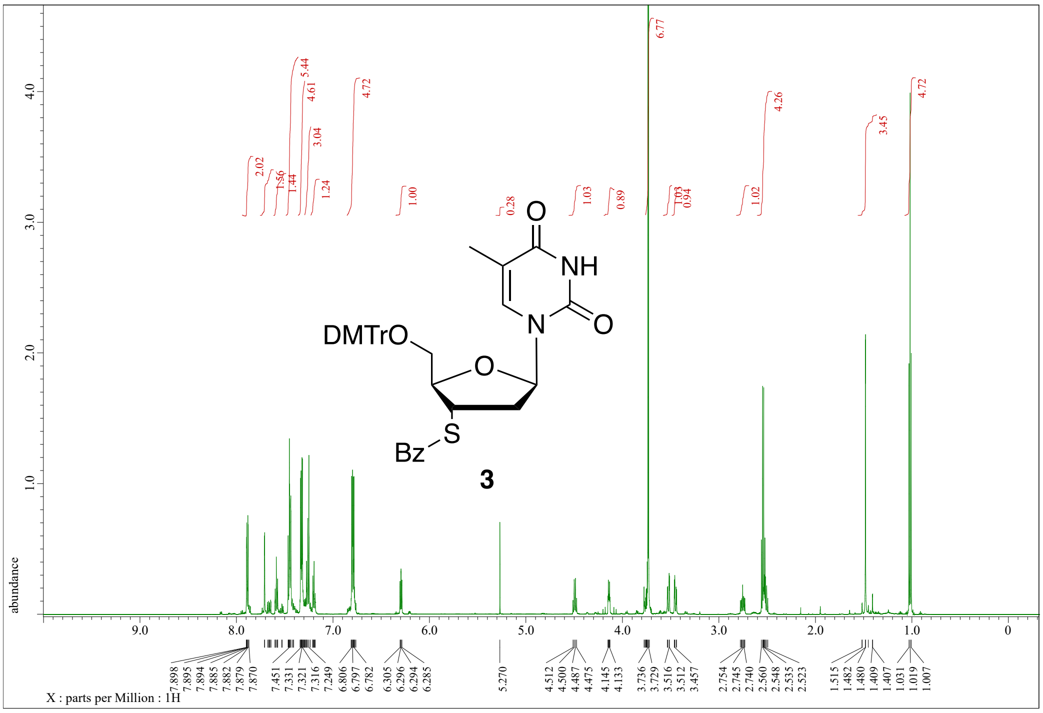

^{1}H NMR Spectrum of Cesium Thiobenzoate (600 MHz, DMSO-d_6)

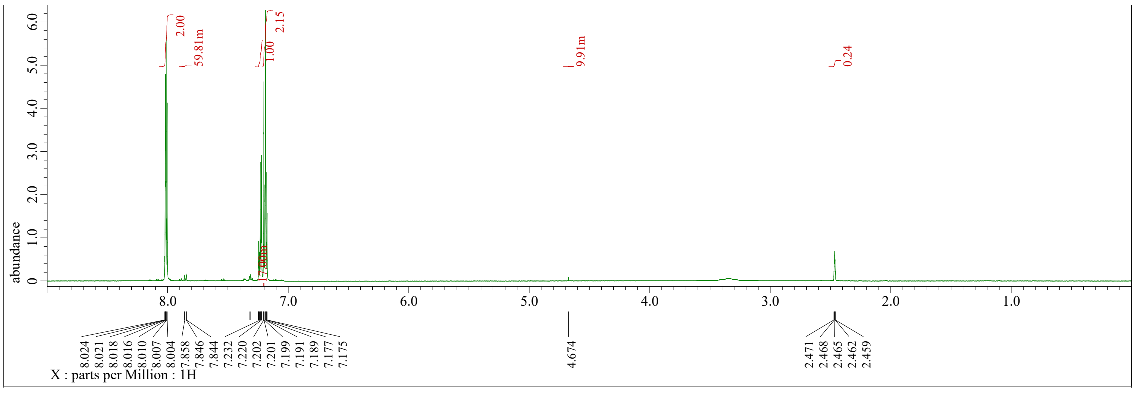

^{13}C NMR Spectrum of Cesium Thiobenzoate (151 MHz, DMSO-d_6)

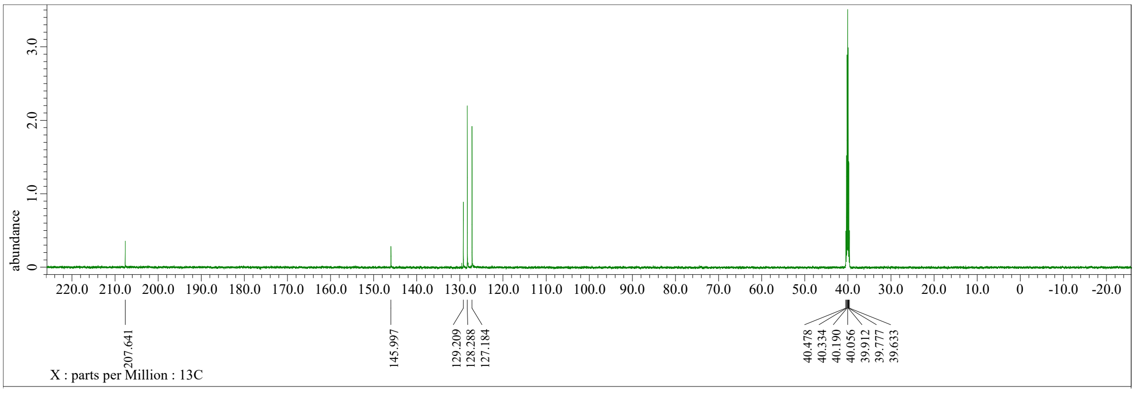

^{1}H NMR Spectrum of Compound **4** (600 MHz, $CDCl_3$)

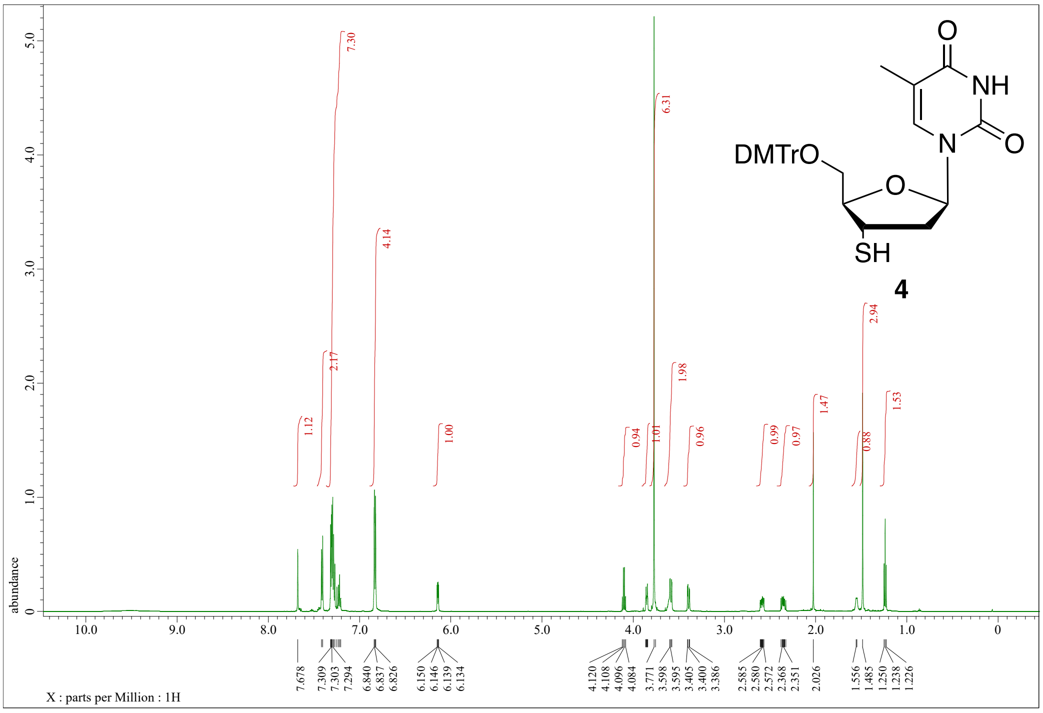

^{1}H NMR Spectrum of Compound **5** (400 MHz, CD_3CN)

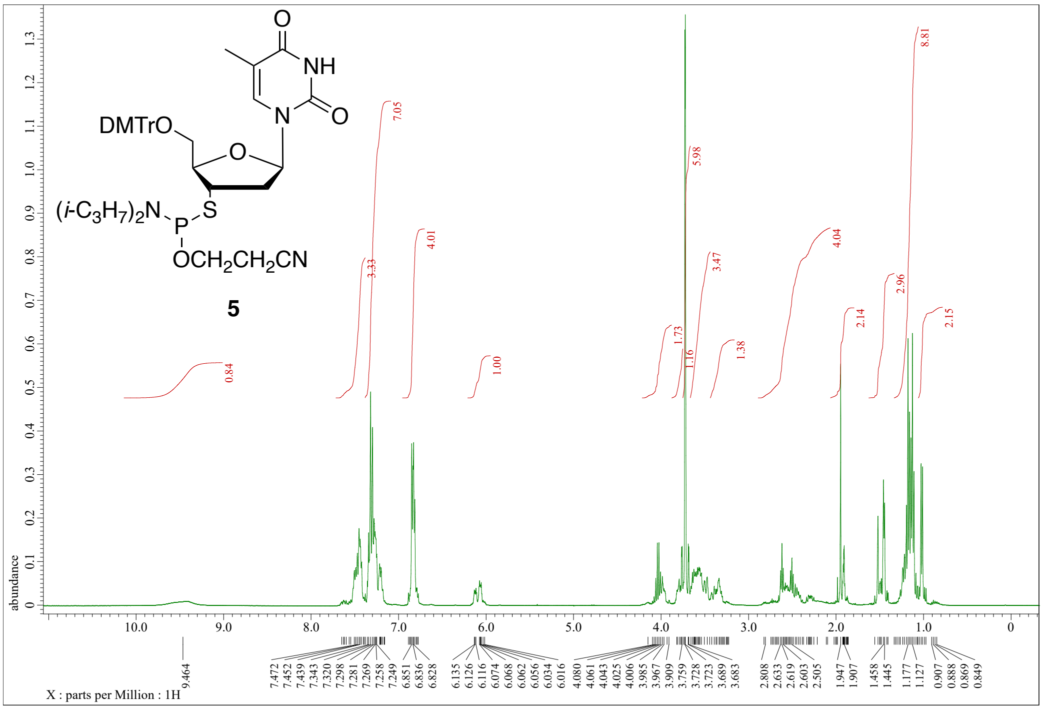

^{31}P NMR Spectrum of Compound **5** (162 MHz, CD_3CN)

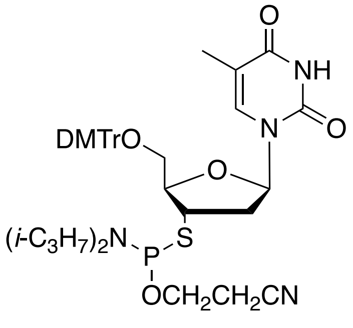

5

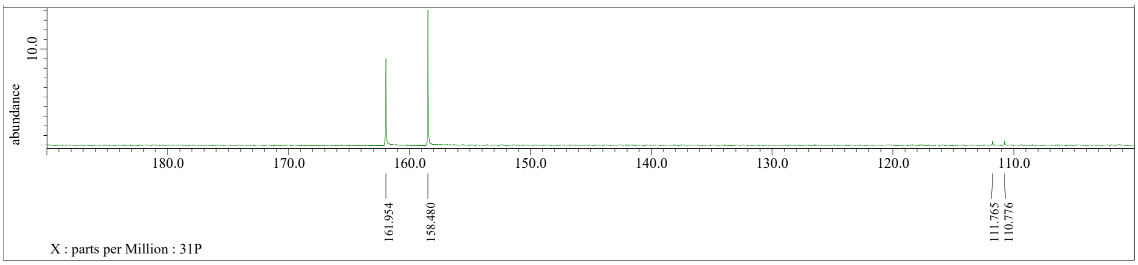

^{1}H NMR Spectrum of Compound **7** (600 MHz, DMSO-d_6)

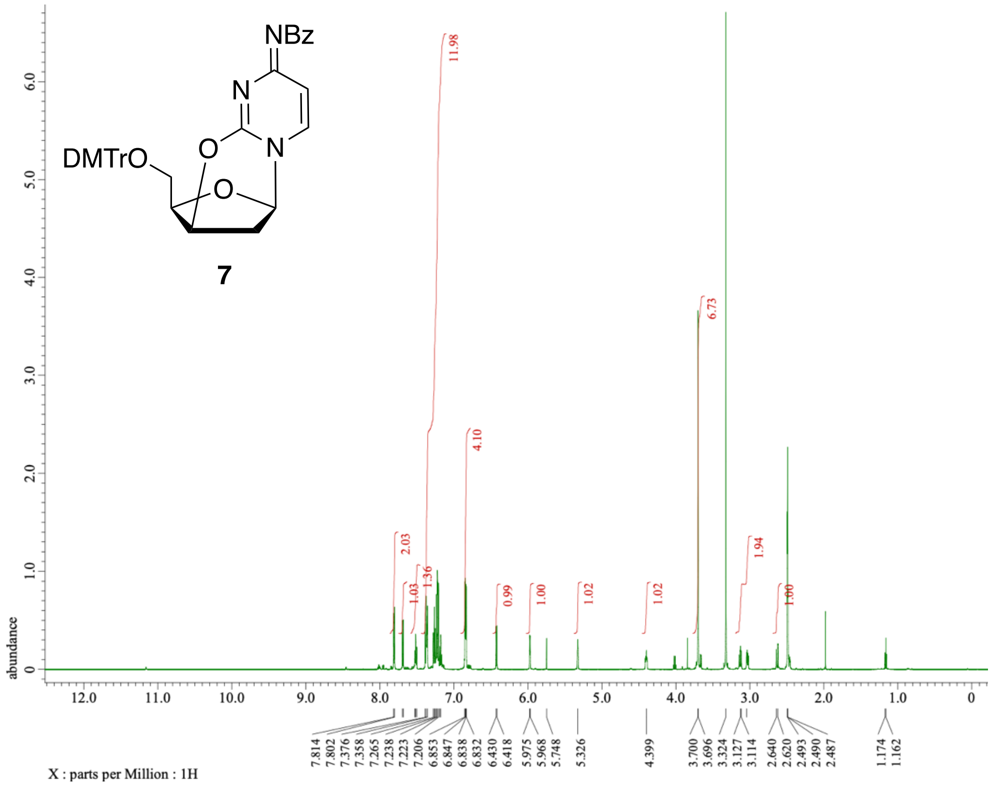

^1H NMR Spectrum of Compound **8** (600 MHz, DMSO-d_6)

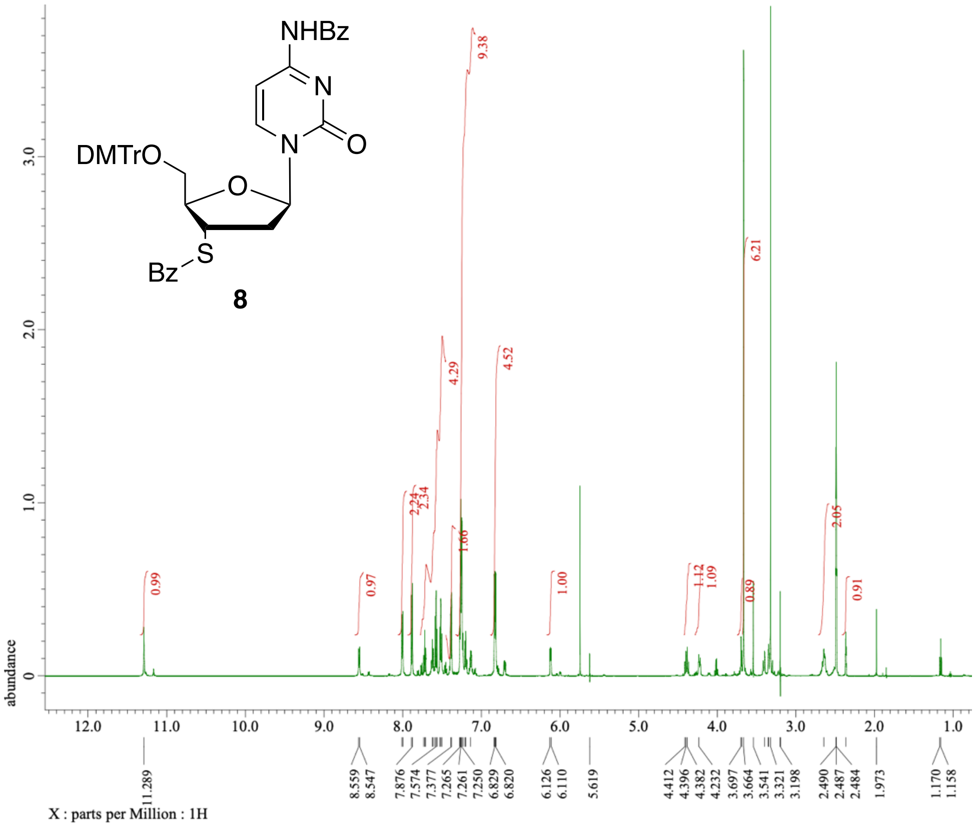

^1H NMR Spectrum of Compound **10** (400 MHz, $CDCl_3$)

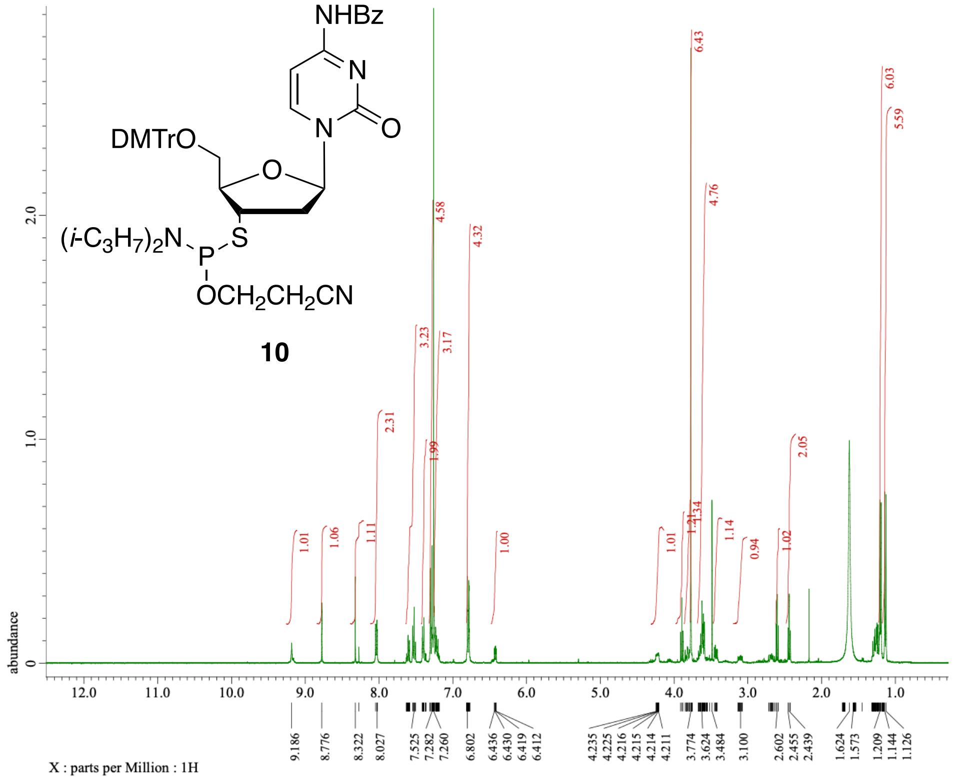

^{31}P NMR Spectrum of Compound **10** (162 MHz, $CDCl_3$)

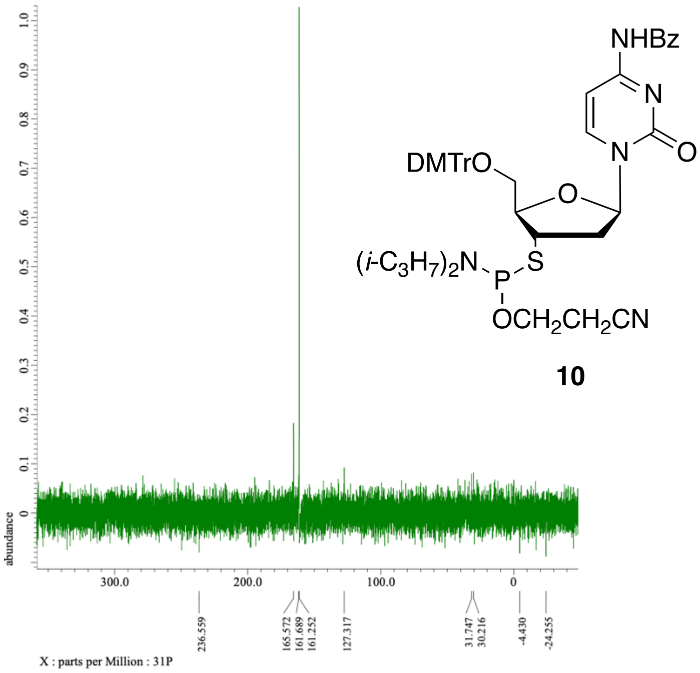

^1H NMR Spectrum of Compound **13** (400 MHz, DMSO-d_6)

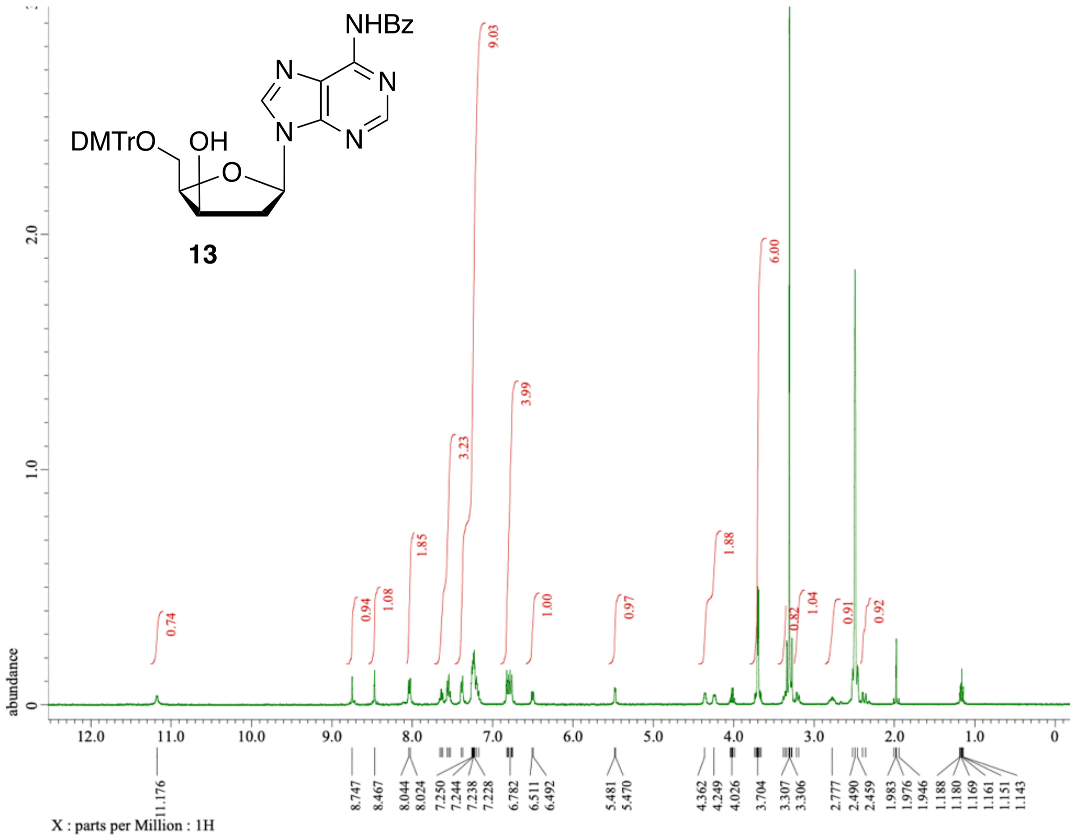

^{1}H NMR Spectrum of Compound **16** (400 MHz, $CDCl_3$)

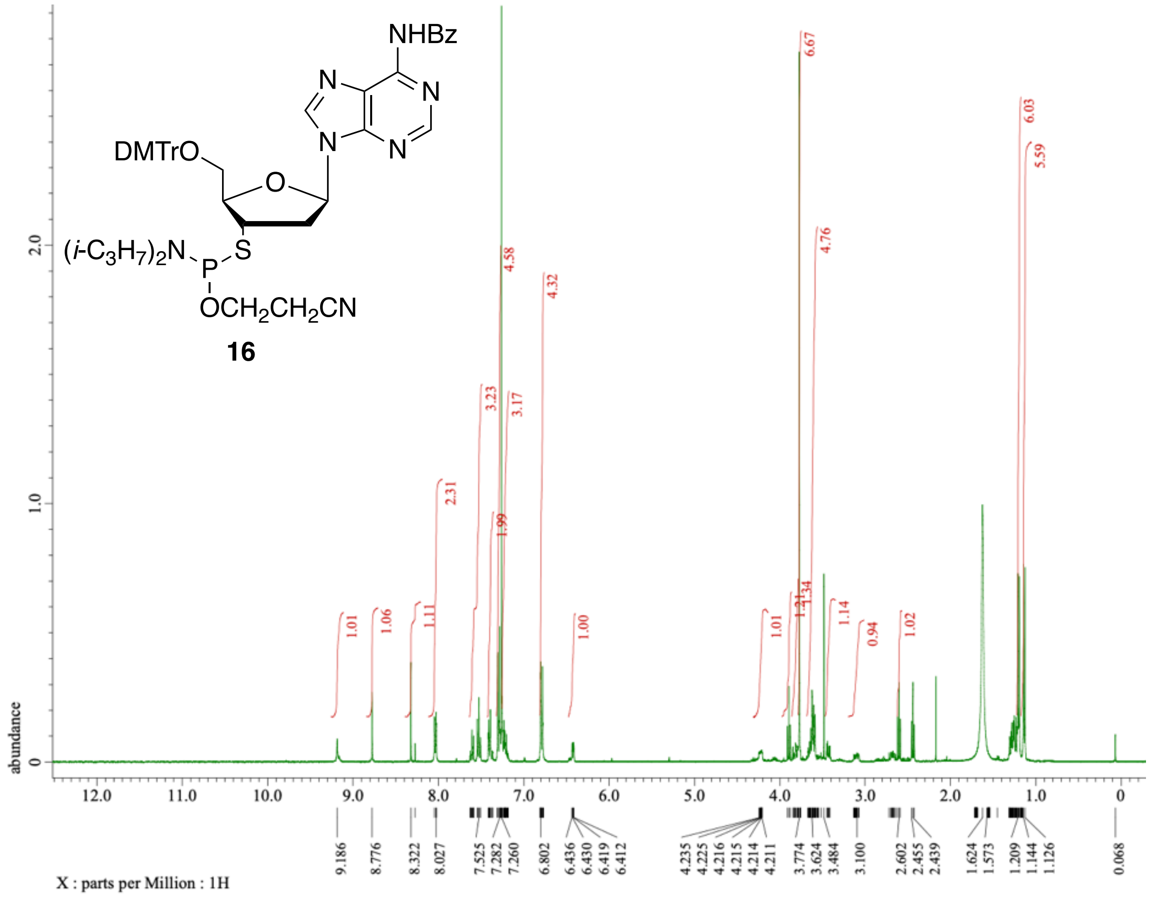

^{31}P NMR Spectrum of Compound **16** (162 MHz, $CDCl_3$)

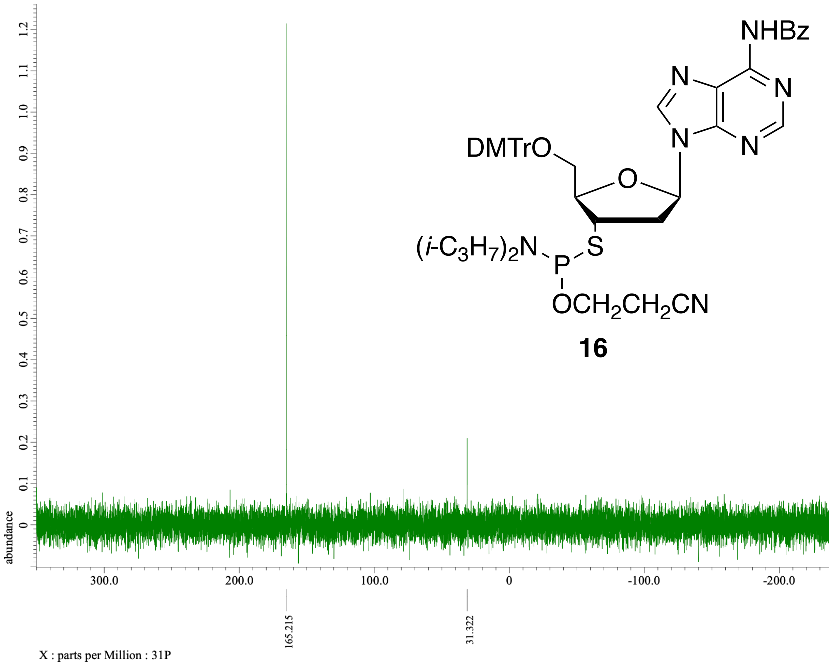

^{1}H NMR Spectrum of Compound **18** (400 MHz, DMSO-d_6)

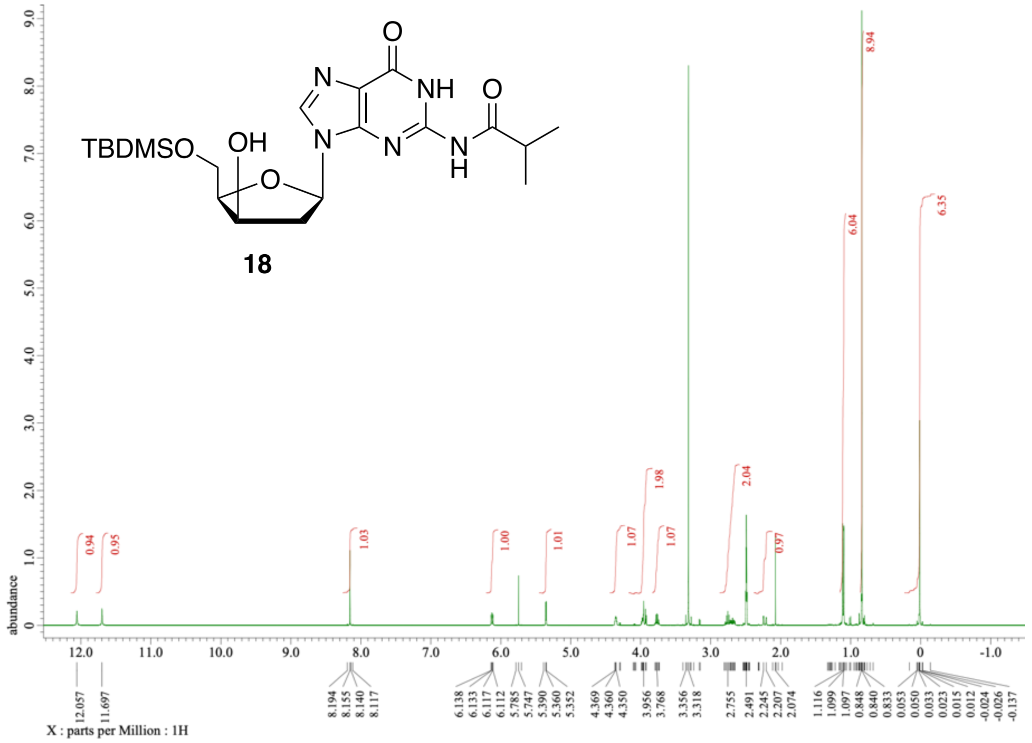

^{1}H NMR Spectrum of Compound **20** (600 MHz, DMSO-d_6)

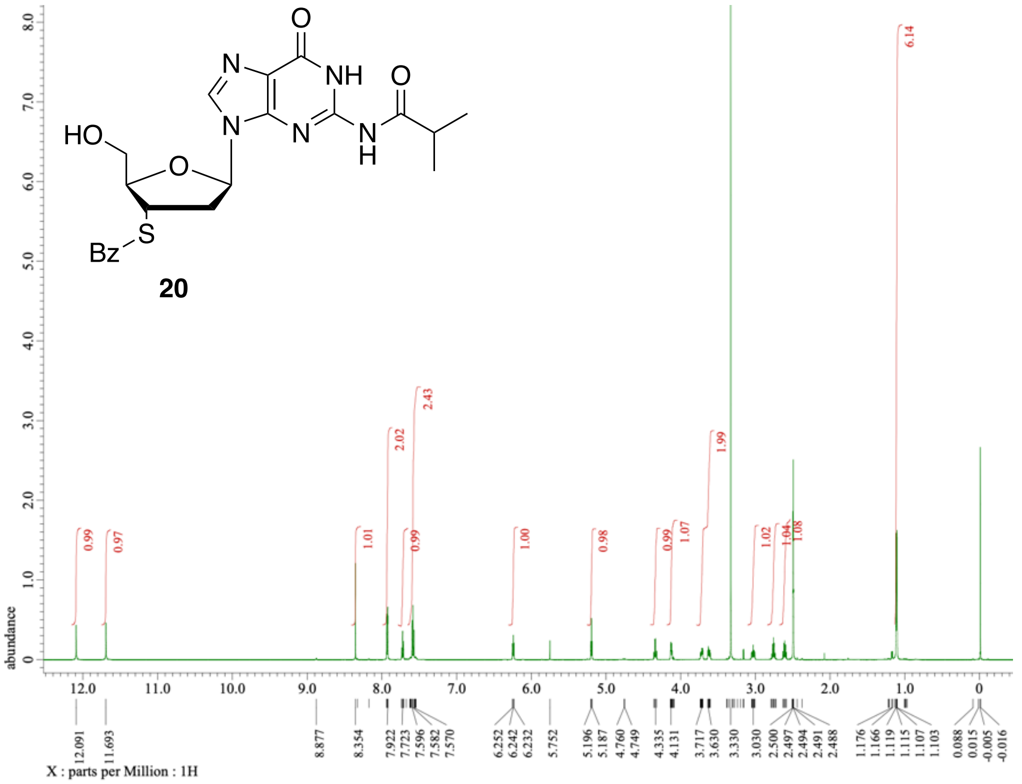

^1H NMR Spectrum of Compound **21** (600 MHz, DMSO-d_6)

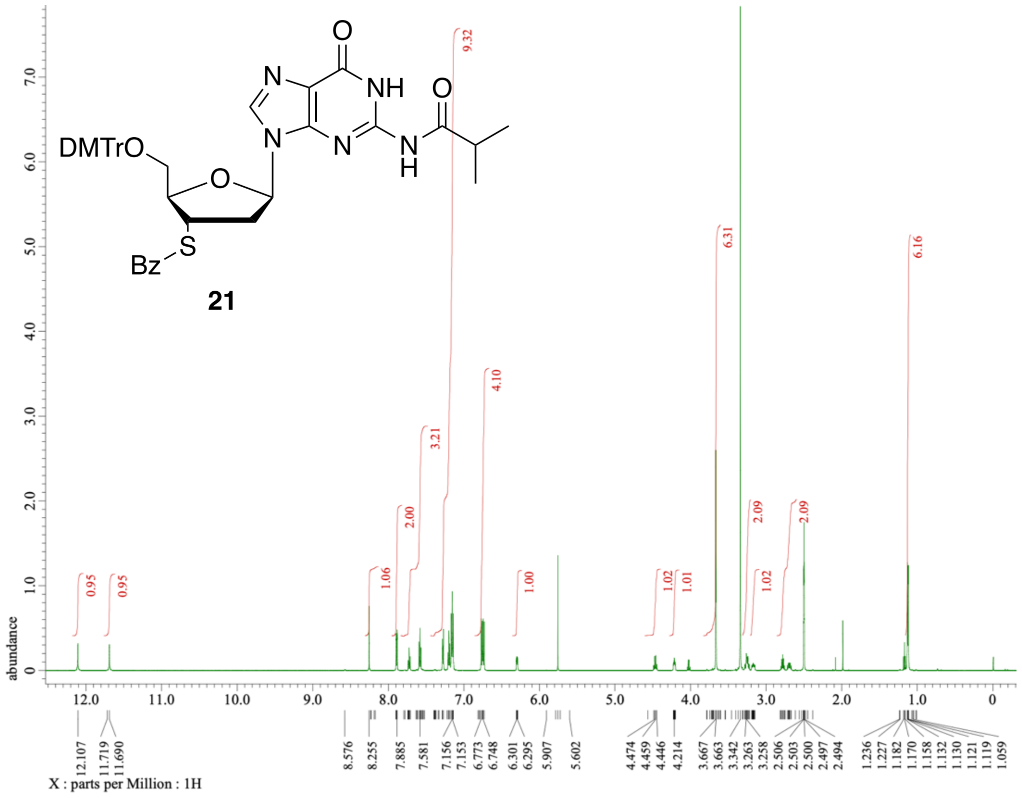

^1H NMR Spectrum of Compound **23** (600 MHz, $CDCl_3$)

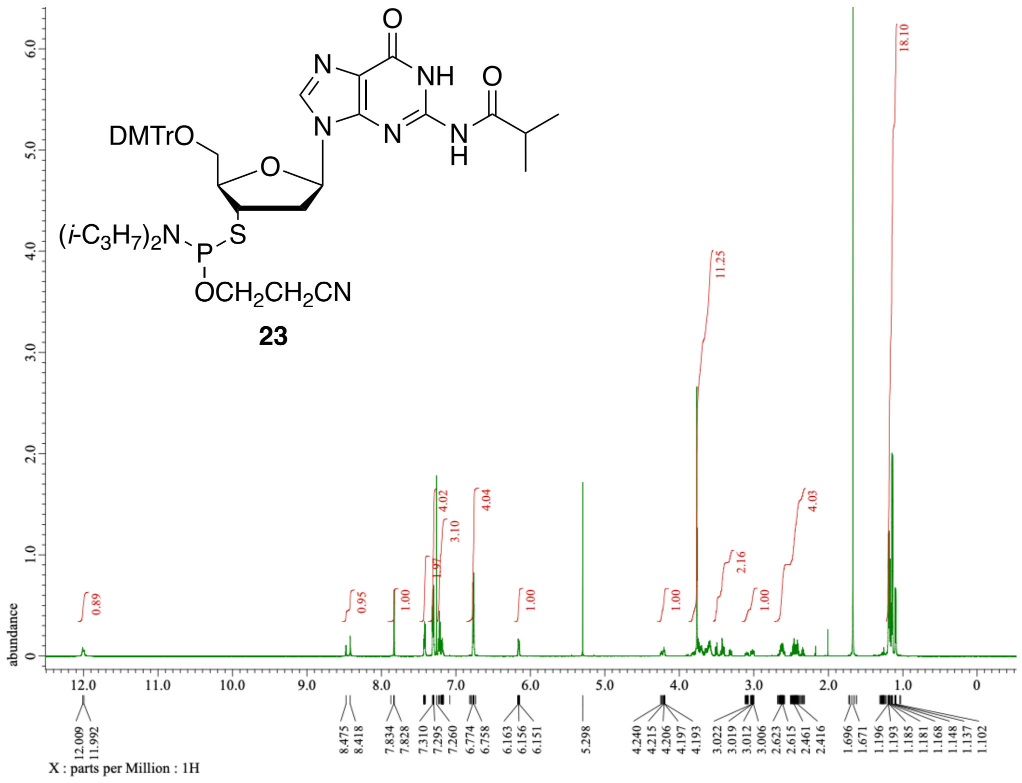

^{31}P NMR Spectrum of Compound **23** (243 MHz, $CDCl_3$)

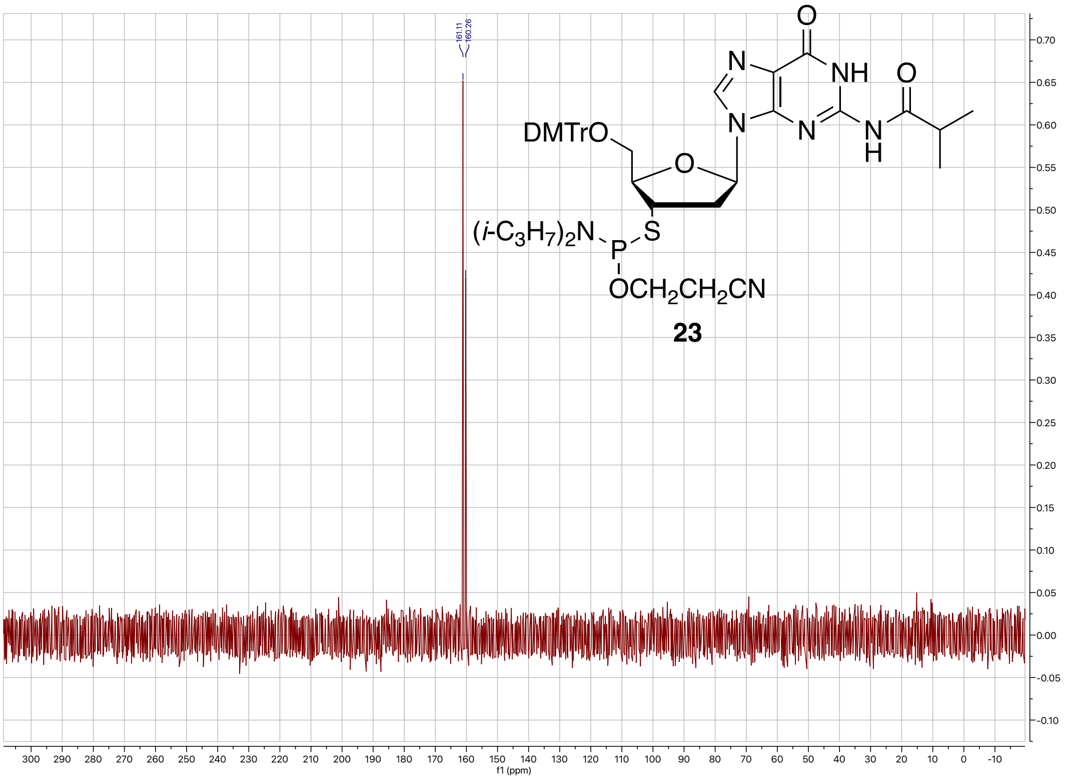

4. Optimization for Synthesis of 3'-Thiobenzoyl Compound

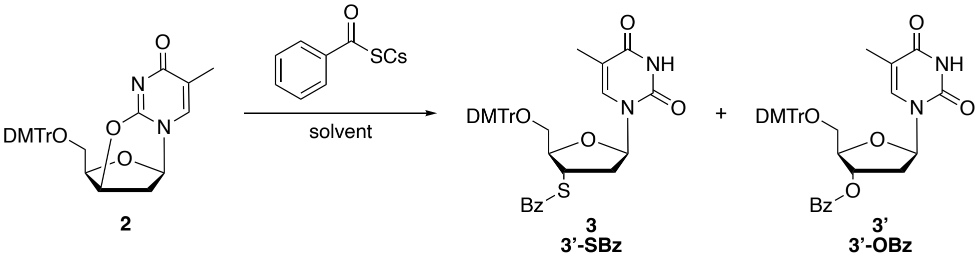

Entry	solvent	CsSBz (eq.)	Reaction Temp.	Reaction Time	Yield	Product
1	N,N-dimethylformamide (DMF)	5.0→9.0 (4 portions)	120°C	17 h	89%	3'-SBz and 3'-OBz
2	Dimethylacetamide (DMA)	5.0	130 °C	1 day	30%	3'-SBz and 3'-OBz
3	acetonitrile	3.7→7.4 (2 portions)	reflux	2 days	50%	3'-SBz Only
4	dioxane	3.7→7.4 (2 portions)	reflux	2 days	91%	3'-SBz Only
5	dioxane	8.0→14 (2 portions)	reflux	2 days	77%	3'-SBz Only

Figure S1. Results of solvent screening for the synthesis of the 3'-thiobenzoyl compound (**3**) showed improved purity and yield. Reactions performed in DMF and DMA produced the 3'-oxybenzoyl compound (**3'**) as a side-product. In contrast, reactions in acetonitrile and dioxane yielded the desired 3'-thiobenzoyl compound (**3**) without side reactions. Using dioxane provided better conversion than acetonitrile, and the portion-wise addition of 7.4 equivalents of cesium benzoate achieved the highest yield of 91% for the desired 3'-thiobenzoyl compound (**3**).

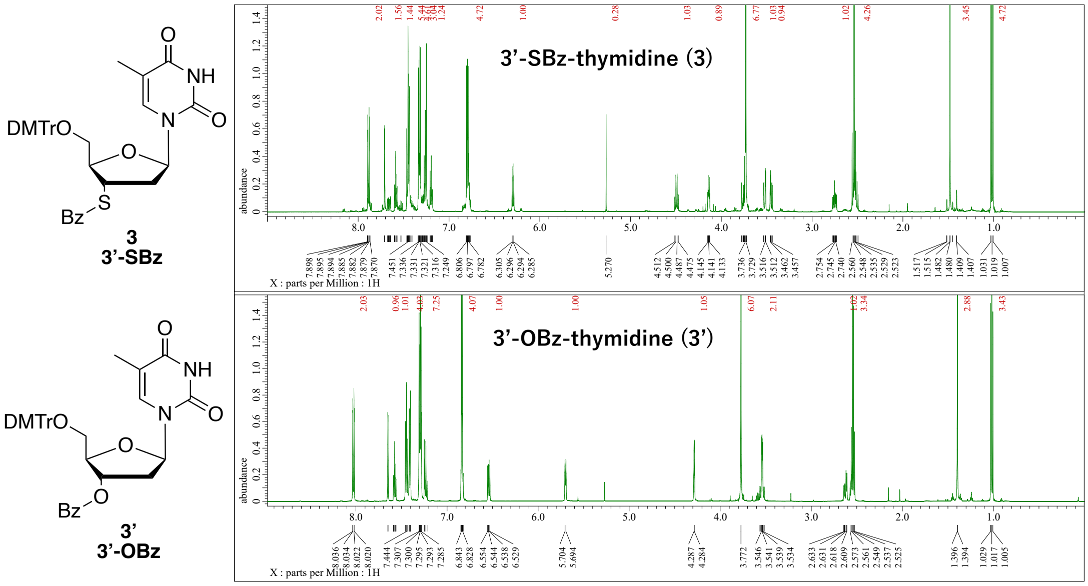

Figure S2. Comparison of ^{1}H NMR Spectra (600 MHz, $CDCl_3$) of the 3'-thiobenzoyl compound (**3**) and the 3'-oxybenzoyl compound (**3'**).

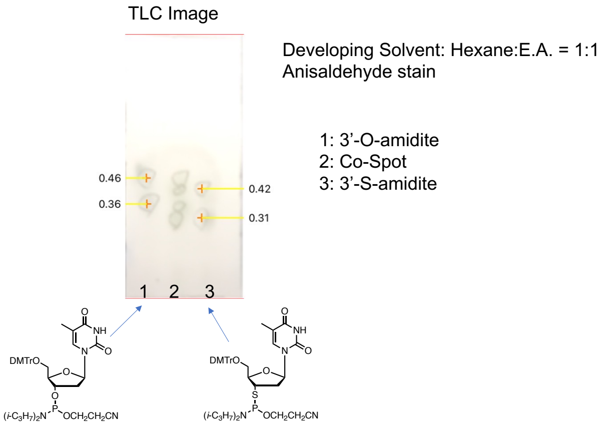

Figure S3. TLC analysis of the 3'-thiothymidine phosphoramidite (5, Lane 3) and its desulfurized compound (Lane 1) revealed that both compounds are mixtures of two diastereomers with very similar Rf values (Lane 2, co-spot). Separation by silica gel column chromatography is challenging due to the close Rf values.

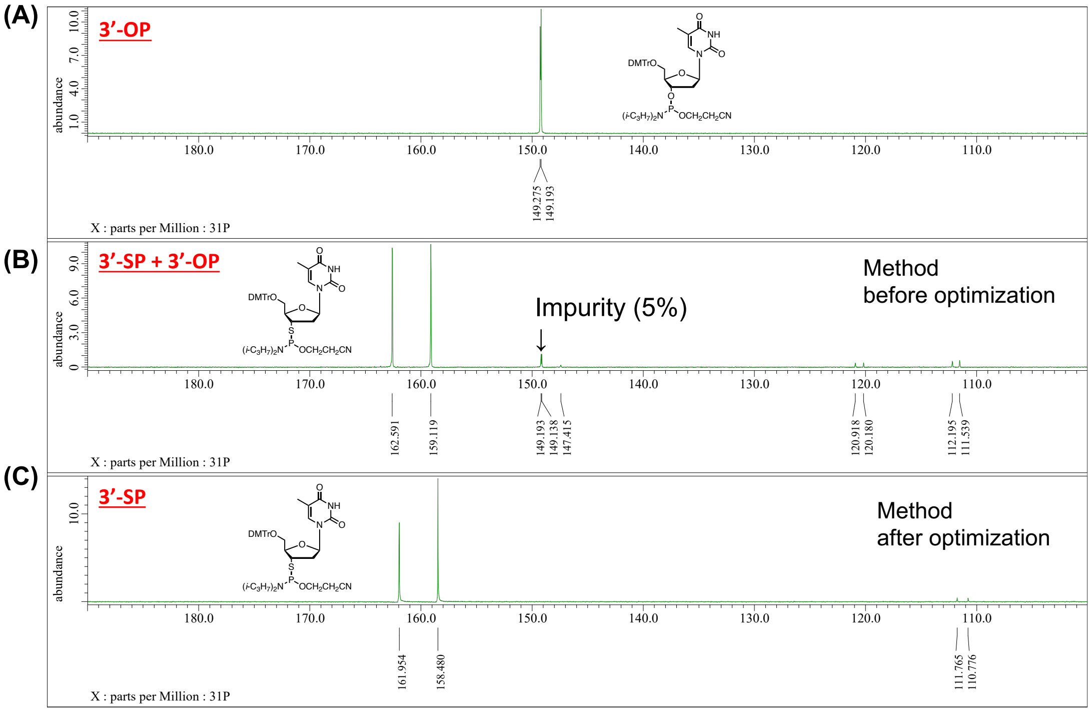

Figure S4. Comparison of ^{31}P NMR spectra (101 MHz, CD_3CN) of the thymidine phosphoramidite (standard sample, ChemGenes) (A), the 3'-thiothymidine phosphoramidite (**5**) prepared from the 3'-thiobenzoyl compound (**3**) synthesized in DMF (before optimization) (B), and the 3'-thiothymifine phosphoramidite (**5**) prepared from the 3'-thiobenzoyl compound (**3**) synthesized in DMF (after optimization) (C). The optimized synthetic method produced the desired 3'-thiothymidine phosphoramidite (**5**) in good purity.

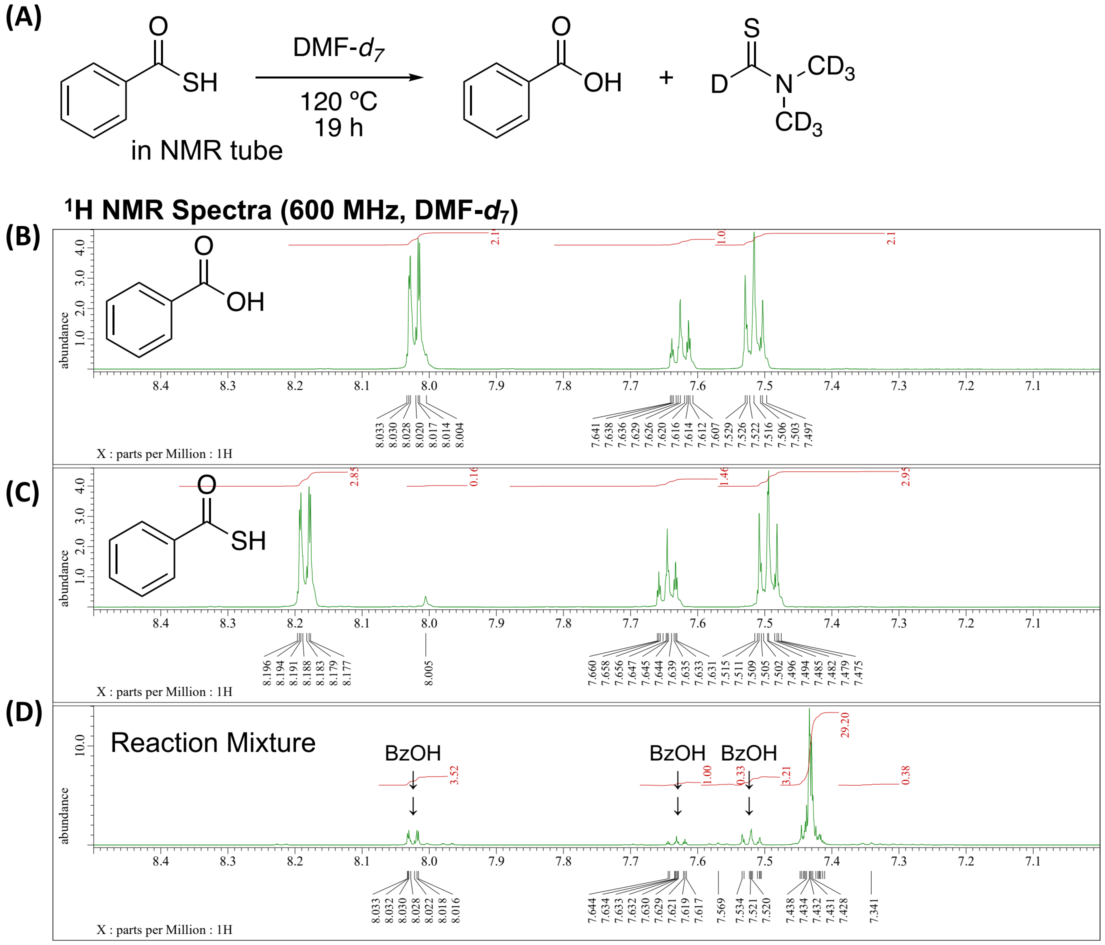

Figure S5. (A) Reaction scheme for the desulfurization of thiobenzoic acid in DMF-d_7. ^{1}H NMR spectra (600 MHz, DMF-d_7) of (B) benzoic acid (authentic sample), (C) thiobenzoic acid (authentic sample), and (D) the reaction product obtained after treating thiobenzoic acid in DMF-d_7 for 19 hours at 120 ºC. The ^{1}H NMR spectrum of the reaction mixture (D) matched the ^{1}H NMR spectrum of benzoic acid (B). This result indicates that the desulfurization of thiobenzoic acid was mediated by DMF, converting it into benzoic acid.

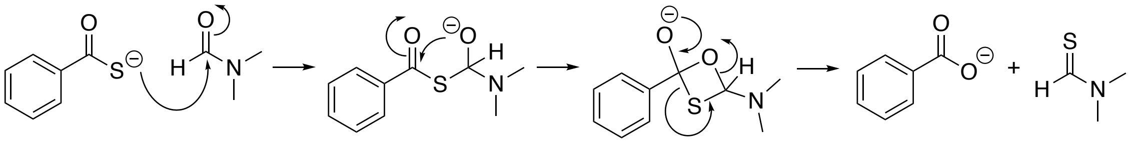

Scheme S5. Plausible mechanism for the desulfurization of thiobenzoate to benzoate via its reaction with DMF. A similar reaction mechanism has been reported, such as the thionation of carbonyl compounds with Lawesson's reagent (7).

5. Synthesized 3'*S*-DNA Characterization by HPLC, MALDI-TOF-MS, and dPAGE

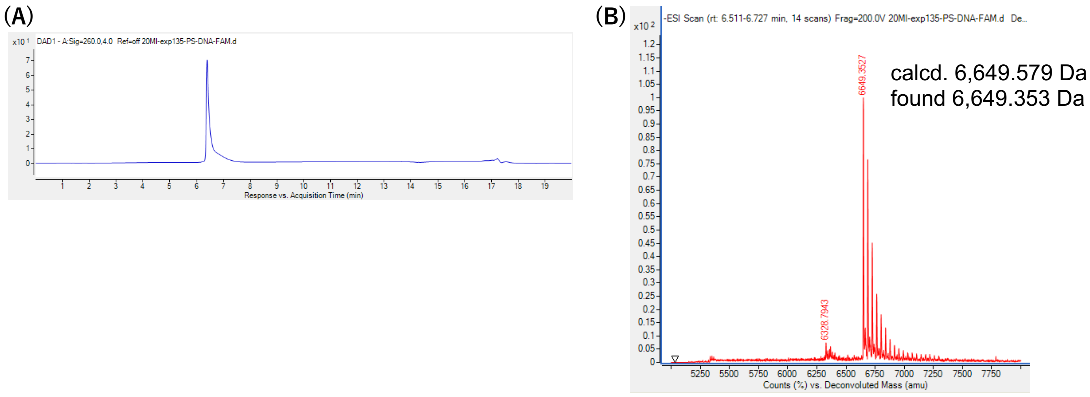

Figure S6. LC-MS analysis of **3'*S* -DNA1**. (A) UPLC profile and (B) Deconvoluted mass spectrum. UPLC System: Agilent 1290 Infinity II, MS System: Agilent 6530 LC/Q-TOF, Column: ACQUITY UPLC Oligonucleotide BEH C18 Column, 130A, 1.7 um, 2.1 mm x 50 mm Part No., 186003949; Serial No., 04133201918322, Solvent A: 100 mM HIFP (pH 8.3) + 8.6 mM TEA Solvent B: 100% MeOH, Column Temperature: 60 ºC, Detection wavelength: 260 nm, Flow rate: 0.3 mL/min, Gradient Program: 0–50%B (0–12 minutes), 50–90%B (12–12.1 minutes), 90%B (12.1–15 minutes), 90–0%B (15–15.1 minutes), 0%B (15.1–20 minutes).

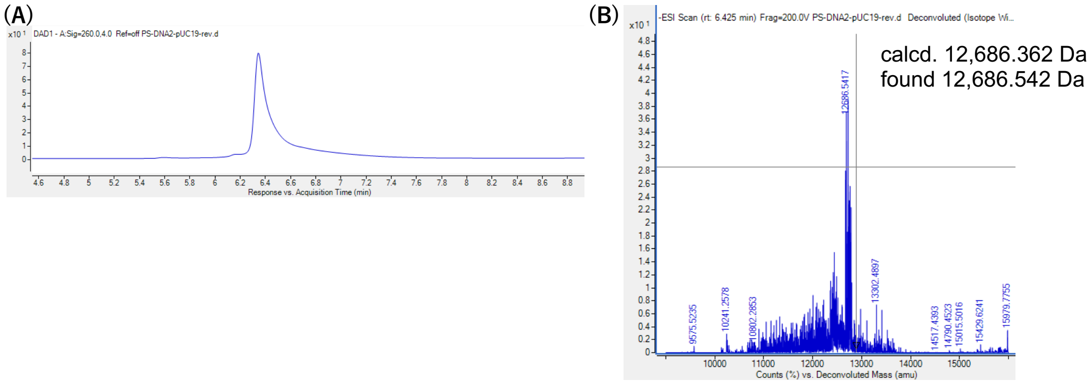

Figure S7. LC-MS analysis of **3'*S* -DNA2**. (A) UPLC profile and (B) Deconvoluted mass spectrum. UPLC System: Agilent 1290 Infinity II, MS System: Agilent 6530 LC/Q-TOF, Column: ACQUITY UPLC Oligonucleotide BEH C18 Column, 130A, 1.7 um, 2.1 mm x 50 mm Part No., 186003949; Serial No., 04133201918322, Solvent A: 100 mM HIFP (pH 8.3) + 8.6 mM TEA Solvent B: 100% MeOH, Column Temperature: 60 ºC, Detection wavelength: 260 nm, Flow rate: 0.3 mL/min, Gradient Program: 0–50%B (0–12 minutes), 50–90%B (12–12.1 minutes), 90%B (12.1–15 minutes), 90–0%B (15–15.1 minutes), 0%B (15.1–20 minutes).

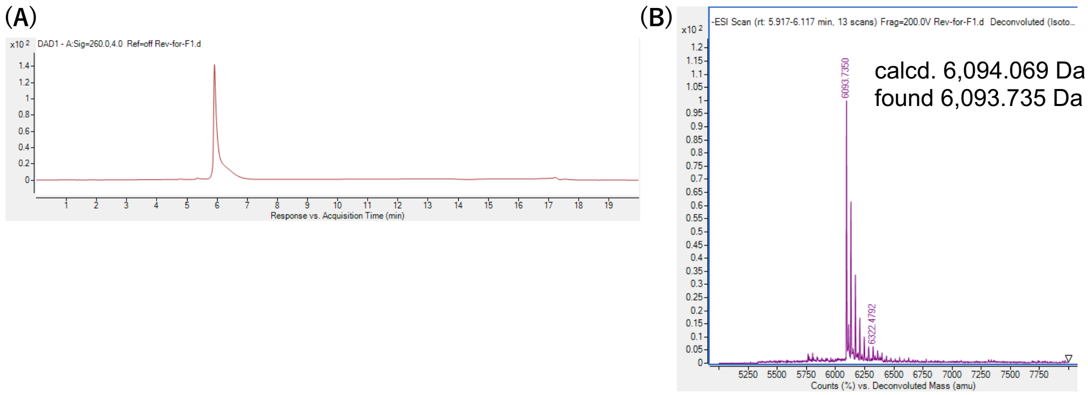

Figure S8. LC-MS analysis of **3'*S* -DNA3**. (A) UPLC profile and (B) Deconvoluted mass spectrum. UPLC System: Agilent 1290 Infinity II, MS System: Agilent 6530 LC/Q-TOF, Column: ACQUITY UPLC Oligonucleotide BEH C18 Column, 130A, 1.7 um, 2.1 mm x 50 mm Part No., 186003949; Serial No., 04133201918322, Solvent A: 100 mM HIFP (pH 8.3) + 8.6 mM TEA Solvent B: 100% MeOH, Column Temperature: 60 ºC, Detection wavelength: 260 nm, Flow rate: 0.3 mL/min, Gradient Program: 0–50%B (0–12 minutes), 50–90%B (12–12.1 minutes), 90%B (12.1–15 minutes), 90–0%B (15–15.1 minutes), 0%B (15.1–20 minutes).

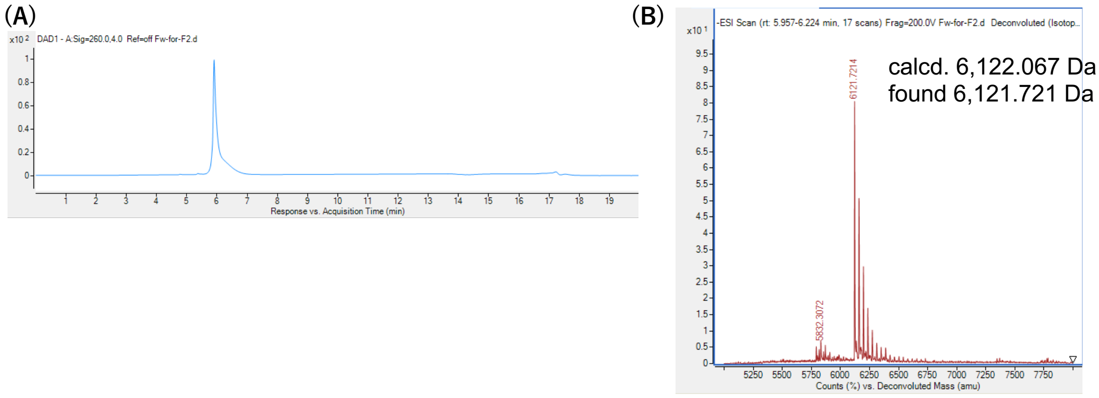

Figure S9. LC-MS analysis of **3'*S* -DNA4**. (A) UPLC profile and (B) Deconvoluted mass spectrum. UPLC System: Agilent 1290 Infinity II, MS System: Agilent 6530 LC/Q-TOF, Column: ACQUITY UPLC Oligonucleotide BEH C18 Column, 130A, 1.7 um, 2.1 mm x 50 mm Part No., 186003949; Serial No., 04133201918322, Solvent A: 100 mM HIFP (pH 8.3) + 8.6 mM TEA Solvent B: 100% MeOH, Column Temperature: 60 ºC, Detection wavelength: 260 nm, Flow rate: 0.3 mL/min, Gradient Program: 0–50%B (0–12 minutes), 50–90%B (12–12.1 minutes), 90%B (12.1–15 minutes), 90–0%B (15–15.1 minutes), 0%B (15.1–20 minutes).

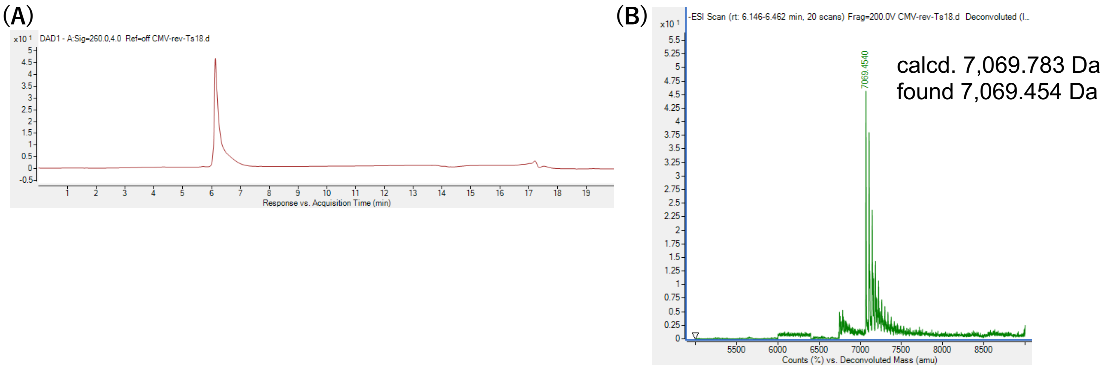

Figure S10. LC-MS analysis of **3'*S*-DNA5.** (A) UPLC profile and (B) Deconvoluted mass spectrum. UPLC System: Agilent 1290 Infinity II, MS System: Agilent 6530 LC/Q-TOF, Column: ACQUITY UPLC Oligonucleotide BEH C18 Column, 130A, 1.7 um, 2.1 mm x 50 mm Part No., 186003949; Serial No., 04133201918322, Solvent A: 100 mM HIFP (pH 8.3) + 8.6 mM TEA Solvent B: 100% MeOH, Column Temperature: 60 ºC, Detection wavelength: 260 nm, Flow rate: 0.3 mL/min, Gradient Program: 0–50%B (0–12 minutes), 50–90%B (12–12.1 minutes), 90%B (12.1–15 minutes), 90–0%B (15–15.1 minutes), 0%B (15.1–20 minutes).

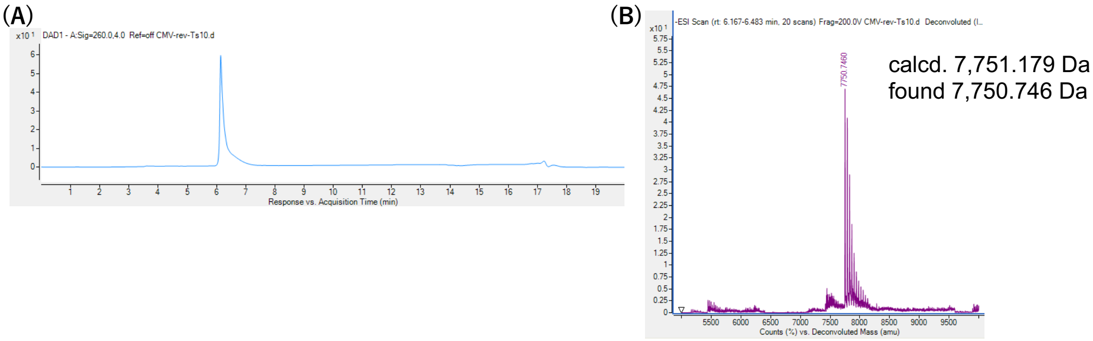

Figure S11. LC-MS analysis of **3'*S*-DNA6.** (A) UPLC profile and (B) Deconvoluted mass spectrum. UPLC System: Agilent 1290 Infinity II, MS System: Agilent 6530 LC/Q-TOF, Column: ACQUITY UPLC Oligonucleotide BEH C18 Column, 130A, 1.7 um, 2.1 mm x 50 mm Part No., 186003949; Serial No., 04133201918322, Solvent A: 100 mM HIFP (pH 8.3) + 8.6 mM TEA Solvent B: 100% MeOH, Column Temperature: 60 ºC, Detection wavelength: 260 nm, Flow rate: 0.3 mL/min, Gradient Program: 0–50%B (0–12 minutes), 50–90%B (12–12.1 minutes), 90%B (12.1–15 minutes), 90–0%B (15–15.1 minutes), 0%B (15.1–20 minutes).

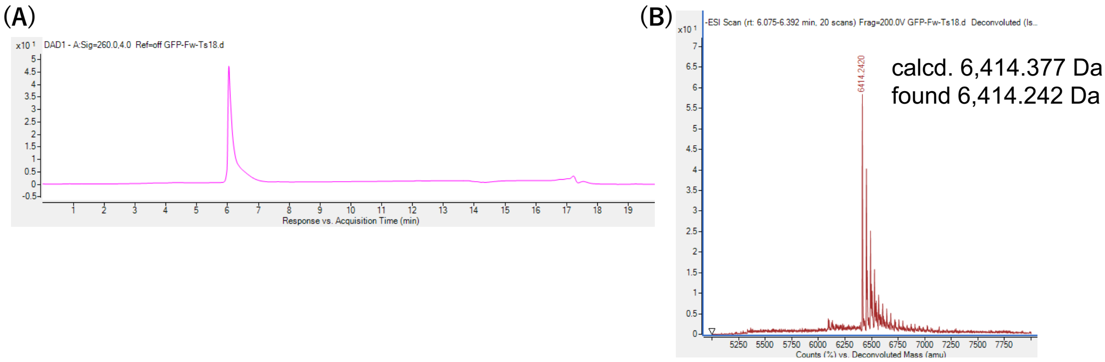

Figure S12. LC-MS analysis of **3'*S*-DNA7**. (A) UPLC profile and (B) Deconvoluted mass spectrum. UPLC System: Agilent 1290 Infinity II, MS System: Agilent 6530 LC/Q-TOF, Column: ACQUITY UPLC Oligonucleotide BEH C18 Column, 130A, 1.7 um, 2.1 mm x 50 mm Part No., 186003949; Serial No., 04133201918322, Solvent A: 100 mM HIFP (pH 8.3) + 8.6 mM TEA Solvent B: 100% MeOH, Column Temperature: 60 ºC, Detection wavelength: 260 nm, Flow rate: 0.3 mL/min, Gradient Program: 0–50%B (0–12 minutes), 50–90%B (12–12.1 minutes), 90%B (12.1–15 minutes), 90–0%B (15–15.1 minutes), 0%B (15.1–20 minutes).

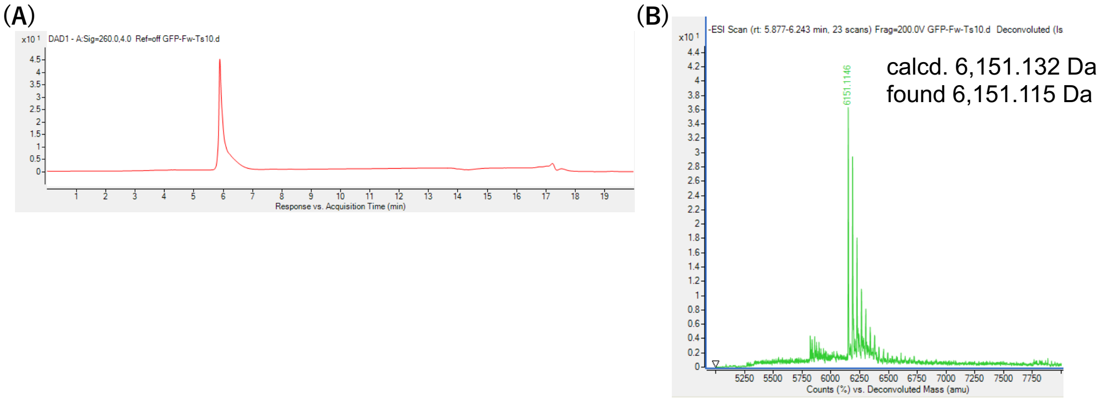

Figure S13. LC-MS analysis of **3'*S*-DNA8**. (A) UPLC profile and (B) Deconvoluted mass spectrum. UPLC System: Agilent 1290 Infinity II, MS System: Agilent 6530 LC/Q-TOF, Column: ACQUITY UPLC Oligonucleotide BEH C18 Column, 130A, 1.7 um, 2.1 mm x 50 mm Part No., 186003949; Serial No., 04133201918322, Solvent A: 100 mM HIFP (pH 8.3) + 8.6 mM TEA Solvent B: 100% MeOH, Column Temperature: 60 ºC, Detection wavelength: 260 nm, Flow rate: 0.3 mL/min, Gradient Program: 0–50%B (0–12 minutes), 50–90%B (12–12.1 minutes), 90%B (12.1–15 minutes), 90–0%B (15–15.1 minutes), 0%B (15.1–20 minutes).

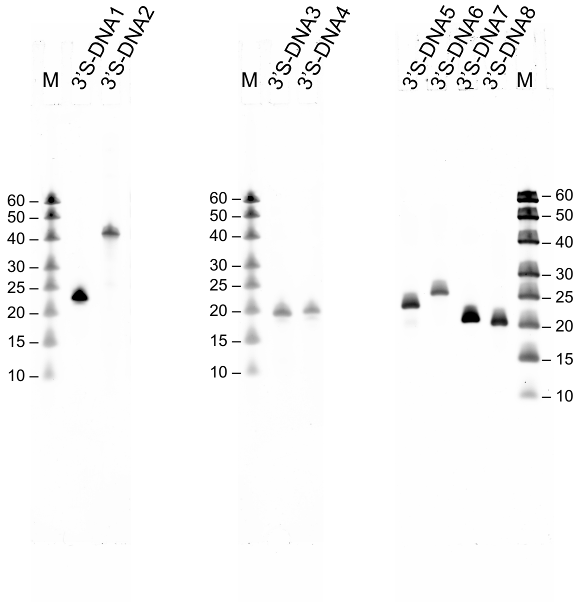

Figure S14. 15% dPAGE analysis of synthesized **3'*S*-DNA1–8** (30 mA constant for 20 minutes, SYBR Green II staining, M: IDT ssDNA 10/60 ladder).

6. Oligonucleotide Strand Cleavage Reaction by Silver Nitrate

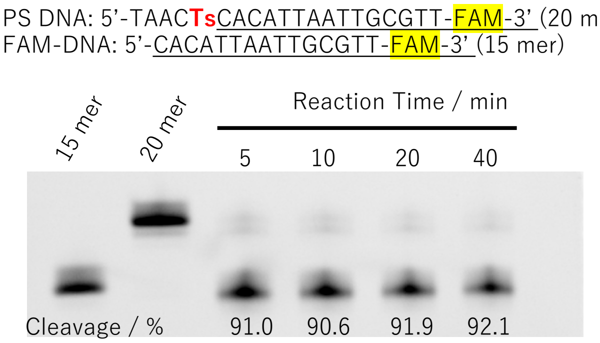

Figure S15. dPAGE analysis of **3'*S*-DNA1** cleavage by silver nitrate treatment (8),(9). The production of 15-mer DNA 5'-pCACATTAATTGCGTT-FAM-3', which is a strand cleavage product by silver nitrate treatment of 20-mer **3'*S*-DNA1**: 5'-TAA**Ts**CACATTAATTGCGTT-FAM-3', was analyzed. The cleavage reaction was carried out at room temperature for 5-40 minutes. The DNA bands were visualized using FAM-derived fluorescence. The result demonstrated that more than 90% of the **3'*S*-DNA1** cleavage within 5 minutes.

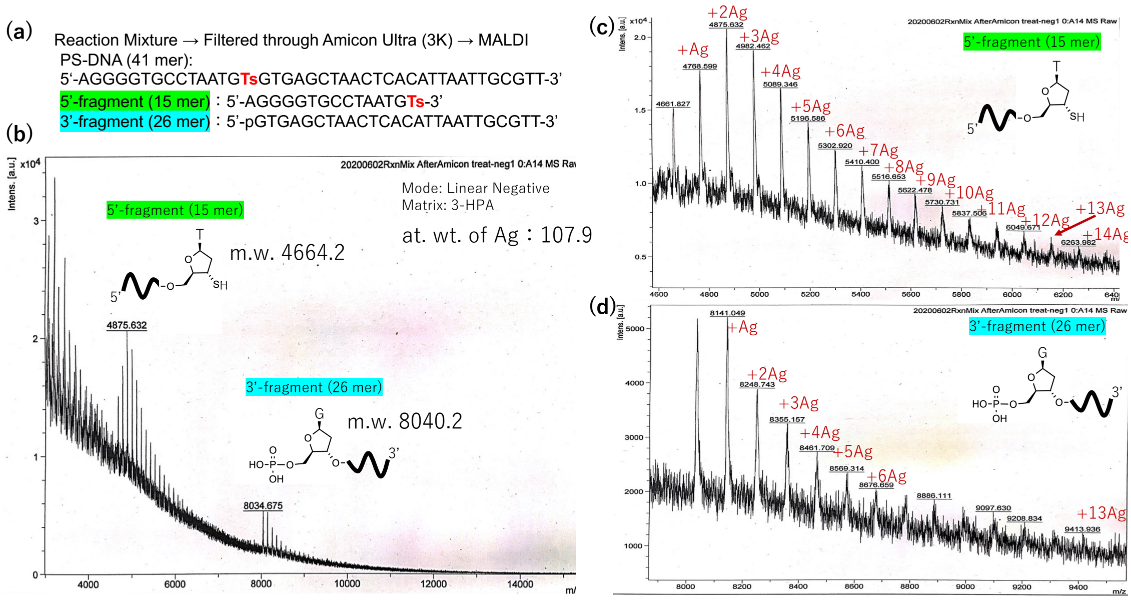

Figure S16. MALDI-TOF-MS spectra showing DNA strand cleavage products by silver nitrate treatment: The cleavage reaction was performed by adding 50 mM silver nitrate aqueous solution to the cleavage target DNA and treating it at 37ºC for 10 hours. The reaction solution was filtered using an ultrafiltration centrifugal filter (Amicon Ultra 3K, manufactured by Merck), and molecules with a molecular weight smaller than 3K were cut off according to the manufacturer's recommended protocol. By this operation, silver ions can be roughly removed from the reaction mixture. The sample after ultrafiltration was collected and subjected to MALDI-TOF-MS analysis (linear negative mode using 3-hydroxypicolinic acid as a matrix). (a) Shows the sequences of the **3'*S*-DNA2** (41-mer) 5'-AGGGGTGCCTAATG**Ts**GTGAGCTAACTCACATTAATTGCGTT-3' and its cleavage product DNA fragment. It is cleaved at the 3'-phosphorothiolate linkage indicated by the arrow in the **Figure**

S16. The 5'-fragment (15-mer) and 3'-fragment (26-mer) are bearing 3'-thiol and 5'-phosphate, respectively. (b) MALDI-TOF-MS spectrum of the cleavage reaction product by silver nitrate treatment. Peaks corresponding to the molecular weights of 466.2 and 8040.2 of the 5'-fragment (15-mer) and 3'-fragment (26-mer) produced by cleavage were observed, suggesting that the desired DNA fragments were produced. (c) An enlarged view of the MALDI-TOF-MS spectrum peak region derived from the cleavage product 5'-fragment (15-mer). (d) An enlarged view of the MALDI-TOF-MS spectrum peak region derived from the cleavage product 3'-fragment (26-mer). (c) and (d) show that the cleavage product exists in the form of multiple silver ions added to the phosphate binding site. On the MALDI-TOF-MS spectrum, peaks with a cluster-like molecular weight distribution with different numbers of added silver ions were detected. These results showed that in 3'-phosphorothiolate-DNA strand cleavage by silver nitrate treatment, it is difficult to remove excessively added silver ions by a simple ultrafiltration process (10). In addition, silver ions strongly bind to chloride ions (Cl^-) contained in buffers used in physiological conditions and biochemical experiments, forming poorly water-soluble AgCl and causing a precipitate (the water solubility of AgCl is 192 µg / 100 mL) (11). Therefore, it has been challenging to apply a cleavage system using silver ions—where silver ions remain in the solution even if the DNA cleavage reaction proceeds with high efficiency—to a ligation reaction following DNA cleavage.

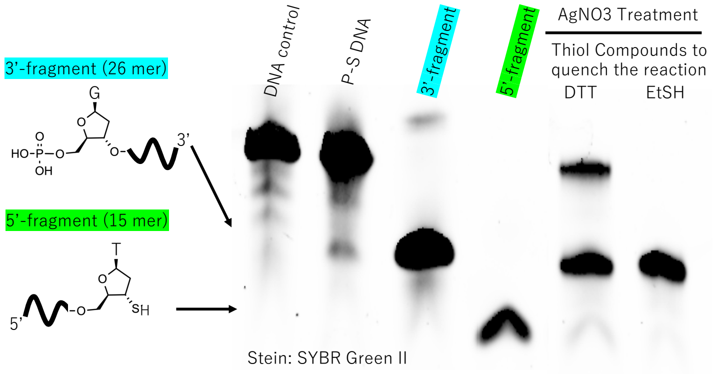

Thiol Compounds	Total DNA Amounts	Recovery of Cleaved DNA Fragments
DTT Treatment	92 pmol	42%
EtSH Treatment	88 pmol	40%

Figure S17. dPAGE analysis of the silver nitrate mediated DNA cleavage reaction and quantification of the cleavage DNA products after the silver ion removal using thiol compounds addition. It has been reported that silver ions can be removed as precipitates due to their high interaction with thiol compounds (12). Therefore, the reaction was stopped by adding dithiothreitol (DTT) or ethanethiol (EtSH) to the reaction mixture, and the produced silver-derived precipitate was removed by centrifugation (8). The supernatant was collected and analyzed by denaturing gel electrophoresis. The 41-mer **3'*S*-DNA2** 5'-AGGGGTGCCTAATG**Ts**GTGAGCTAACTCACATTAATTGCGTT-3' was used as the cleavage target DNA, and it was cleaved at the 3'-phosphorothiolate linkage, resulting in a 5'-fragment (15-mer) 5'-AGGGGTGCCTAATGTs-3') and 3'-fragment (26-mer) 5'-pGTGAGCTAACTCACATTAATTGCGTT-3'. Strand cleavage by silver nitrate was performed by incubation for 1 day at room temperature. SYBR Green II was used for DNA staining by gel analysis, and bands on the gel were visualized by detecting fluorescence derived from SYBR Green II bound to DNA. When DTT was used to

terminate the reaction, the bands derived from the cleavage products 5'-fragment and 3'-fragment as well as the thiol moiety of the 5'-fragment dimerized by disulfide bonds were observed. The band derived from dimerization had almost the same gel mobility as the 41-mer **3'*S*-DNA2**, it was difficult to distinguish. However, the gel band was cut and extracted the DNA from the gel piece. After extraction, MALDI-TOF-MS analysis revealed that it was a dimerized form of 5'-fragment. When EtSH was used to terminate the reaction, the band derived from the 41-mer **3'*S*-DNA2**, was completely disappeared, and the 3'-fragment (26-mer), 5'-pGTGAGCTAACTCACATTAATTGCGTT-3' was only observed on the gel. The cleavage product on the 5' side, 5'-fragment (15-mer) 5'-AGGGGTGCCTAATGTs-3', tightly bound to silver ion and formed the precipitate with EtSH-Ag complex. Furthermore, the amount of DNA contained in the supernatant after removal of silver ion precipitation by addition of thiols was determined by measuring the absorbance of DNA at 260 nm, and the results are shown in the table. The total amount of DNA per reaction solution was 220 pmol, but the amount of cleavage product DNA recovered was 92 pmol in the DTT addition system and 88 pmol in the EtSH addition system, and the recovery rate was 40% in both systems. These results show that in the silver ion precipitation removal method using thiols, about 60% of the cleavage product DNA co-precipitates with silver ions, reducing the yield of the cleavage product. Therefore, it is considered that silver nitrate mediated 3'*S*-DNA cleavage is difficult to apply as the practical technique.

7. Comparison of Silver Nanoparticle and Silver Nitrate for Oligonucleotide Strand Cleavage

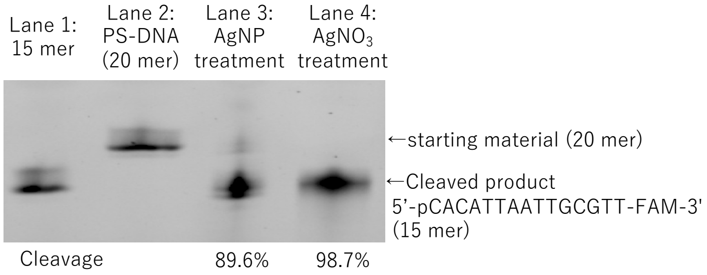

Figure S18. Comparison of silver nitrate treatment and silver nanoparticle treatment in 3'*S*-DNA strand cleavage reaction. In the gel image, Lane 1 is the analysis of DNA (15-mer) 5'-CACATTAATTGCGTT-FAM-3', which has the same base length as the DNA generated after cleavage. Lane 2 is the analysis of **3'*S*-DNA2** (20-mer) 5'-TAA**Ts**CACATTAATTGCGTT-FAM-3'. Lane 3 is the analysis of the cleavage product when 3'*S*-DNA strand cleavage was performed using silver nanoparticles with a particle size of 100 nm. The reaction was performed by adding silver nanoparticles and incubating at 90 ºC for 25 hours. The silver nanoparticles in the reaction solution were removed as a precipitate by centrifugation at 15,000 rpm for 1 hour, and then electrophoresis was performed. The results showed that almost all of the **3'*S*-DNA2** (20 mer) disappeared and was converted to cleavage product DNA (15-mer) (89.6%). Lane 4 is the analysis of the cleavage product when 3'*S*-DNA strand cleavage was performed using silver nitrate. The reaction was performed by adding silver nitrate and then incubating at room temperature for 1.5 hours. Silver ions in the reaction solution were removed as a precipitate by adding DTT, and then electrophoresis was performed. The results showed that the **3'*S*-DNA2** (20-mer) was cleaved and converted to the cleavage product DNA (15-mer) (98.7%).

Table S2. Comparison of recovery amount of DNA fragments after cleavage between silver nitrate treatment and silver nanoparticle treatment.

Cleaving Agent	Molar Amount of Cleaved Product DNA (pmol)	Recovery Yield of Cleaved Product (%)
$AgNO_3$	35.1	14.4
Silver Nanoparticle (100 nm)	240	98.4

In the case of silver nitrate treatment, the DNA content in the supernatant after the precipitation of silver ions—removed by the addition of DTT—was quantified by measuring the DNA-derived absorbance at 260 nm. In the case of silver nanoparticle treatment, after removing the silver nanoparticles as a precipitate by centrifugation, the amount of DNA contained in the supernatant was quantified by measuring the absorbance of the DNA at 260 nm. As a result, the total amount of DNA per reaction solution was 244 pmol, whereas in the case of silver nitrate treatment, the amount of cleavage product DNA recovered was 35.1 pmol, the recovery rate was only 14.4%. This result indicates that the most of the cleavage products was co-precipitated with silver-DTT complex during the removal of silver ions by precipitation. On the other hand, in the case of silver nanoparticle treatment, the amount was 240 pmol, and the cleavage product DNA was recovered almost quantitatively. These results show that the 3'*S*-DNA cleavage method using silver nitrate is difficult to use due to a significant decrease in yield. Still, the cleavage method using silver nanoparticles can recover the cleavage products efficiently and is practically applicable to the novel DNA cleavage techniques.

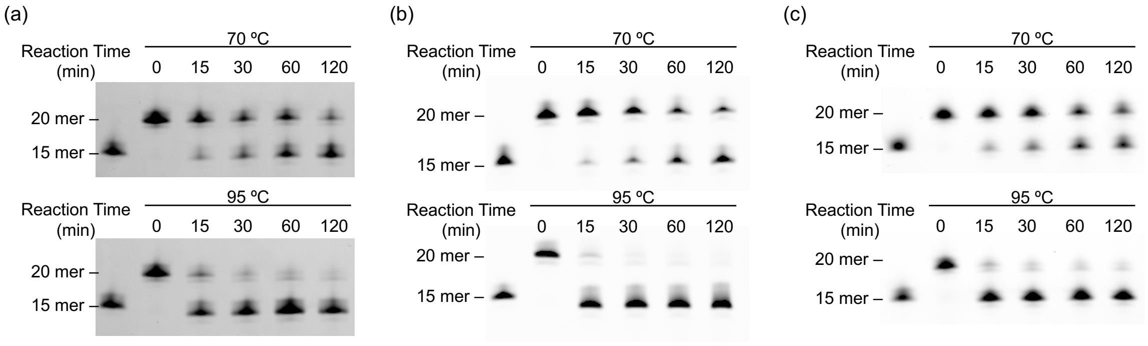

(d)

Cleavage / %					
Reaction Time	0 min	15 min	30 min	60 min	120 min
70°C	0	19.5	42.7	51.8	72.7
	0	19.7	35.7	51.4	59.7
	0	19.7	30.4	49.4	55.8
average	0	19.6	36.3	50.9	62.7
S.D.	0	0.1	6.2	1.3	8.8
95°C	0	68.2	88.1	90.4	91.4
	0	78.5	87.8	89.9	92.1
	0	81.9	87.7	89.4	91.1
average	0	76.2	87.9	89.9	91.5
S.D.	0	7.1	0.2	0.5	0.5

Figure S19. Silver nanoparticle-mediated DNA strand cleavage. **3'*S*-DNA1** (5'-TAA**Ts**CACATTAATTGCGTT-FAM-3', 3μM, 10 μL) was incubated with an AgNP dispersion (10 nm, 0.02 mg/mL, 50 μL) at either 70 °C or 95 °C for 1–120 minutes. The reaction mixtures were analyzed by 15% denaturing PAGE (30 mA, 20 min). To confirm reproducibility, the same experiment was repeated three times (a–c). (d) DNA strand cleavage efficiencies and calculation of the standard deviation (S.D.).

8. Absorption Spectra of Silver Nanoparticles

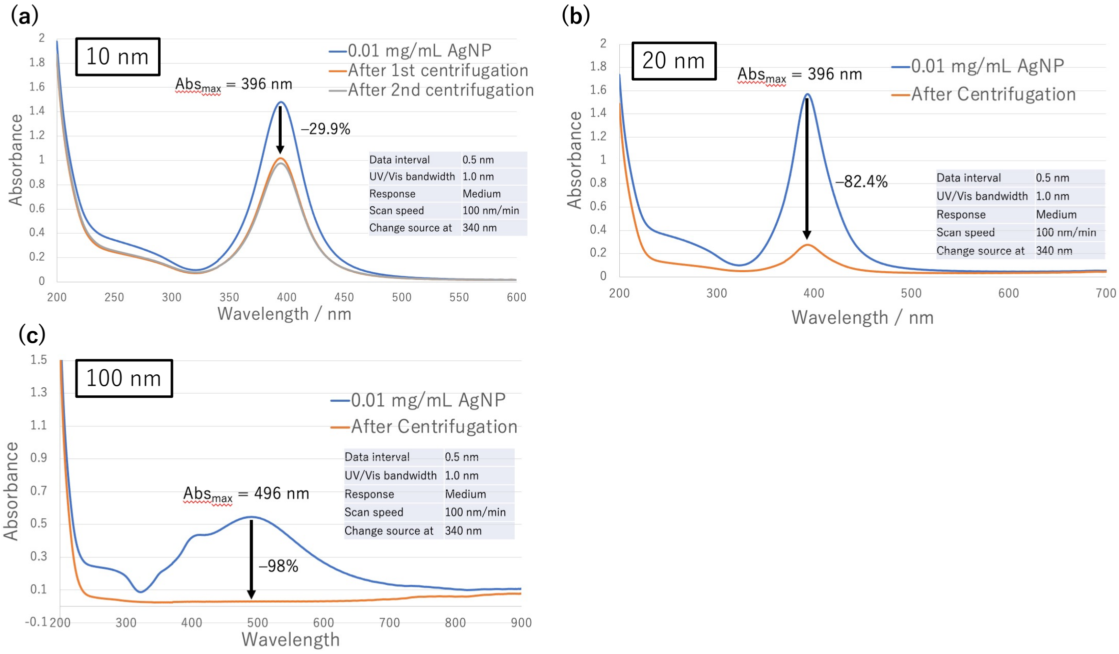

Figure S20. Absorption spectra of silver nanoparticle dispersions before and after centrifugation showing nanoparticle size dependency for the removal of silver nanoparticles. Silver nanoparticles are known to exhibit absorption in the range of 350 to 700 nm depending on the particle size due to the surface plasmon resonance effect. Therefore, by obtaining the absorption spectrum of the dispersion, the amounts of nanoparticles present in the dispersion can be estimated from the absorbance. (a) Absorption spectra of silver nanoparticles dispersion with a particle size of 10 nm. (b) Absorption spectra of silver nanoparticles dispersion with a particle size of 20 nm. (c) Absorption spectra of silver nanoparticle dispersions with a particle size of 100 nm. The blue spectrum in the **Figure S20** is derived from a 0.01 mg/mL silver nanoparticle dispersion. The orange spectrum in the **Figure S20** is the result after centrifuging the nanoparticle dispersion at 15,000 rpm for 1 hour to precipitate the nanoparticles. By comparing the Abs_{Max} before and after centrifugation, we can evaluate the removal efficiency of nanoparticles by centrifugation. As a result, the calculated removal efficiency of the silver nanoparticle with a particle diameter of 10 nm was 29.9%, a particle diameter of 20 nm was 82.4%, and a particle diameter of 100 nm was almost 100%. This result means that the nanoparticles floating in the dispersion liquid were precipitated by the centrifugation operation. Especially, silver nanoparticles with a particle size of 20–100 nm can be clearly removed from the reaction solution as a precipitate by centrifugation.

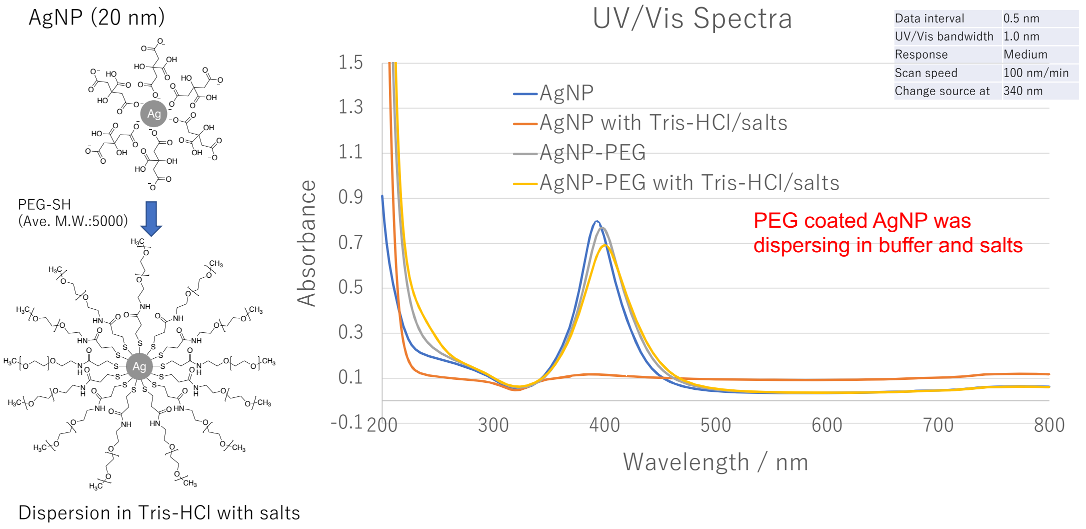

Figure S21. Absorption spectra of silver nanoparticle dispersions demonstrating the improved dispersion property of nanoparticles by PEGylation even though the presence of buffer/salts (13). Surface modification of the silver nanoparticle was performed by mixing the silver nanoparticle dispersion (0.02 mg/mL, particle size 20 nm, in sodium citrate buffer) and 23.9 g/L aqueous solution of *O*-[2-(3-mercaptopropionylamino)ethyl]-*O*'-methylpolyethylene glycol (average molecular weight 5,000, Sigma-Aldrich). The dispersibility of nanoparticles was evaluated by measuring the absorption spectrum based on the surface plasmon resonance effect of the nanoparticle dispersion. The blue spectrum in the **Figure S21** is an absorption spectrum derived from a commercially available silver nanoparticle dispersion, and maximum absorption was observed at 396 nm. In the presence of Tris-HCl buffer (pH 8.3) which is commonly used in biochemical experiments such as ligation using DNA ligases, and aqueous solutions of potassium chloride/magnesium chloride as salts, the absorption at around 396 nm of silver nanoparticle dispersion disappeared. This result indicates that the surface of the silver nanoparticles was coated with AgCl by adding a buffer or salt, and the nanoparticles bonded to each other due to van der Waal's interaction and precipitated as aggregates (11). On the other hand, surface PEG-modified silver nanoparticles also have absorption characteristics comparable to commercially available silver nanoparticles, as shown in the gray spectrum, but surface PEG-modified silver nanoparticles do not absorb buffers or salts, as shown in the orange spectrum. Even in addition, strong absorption was observed around 396 nm. This indicates that the surface of the PEGylated silver nanoparticles is protected by the excluded volume effect of PEG, and exhibits high dispersibility even in the presence of buffers and salts (13).

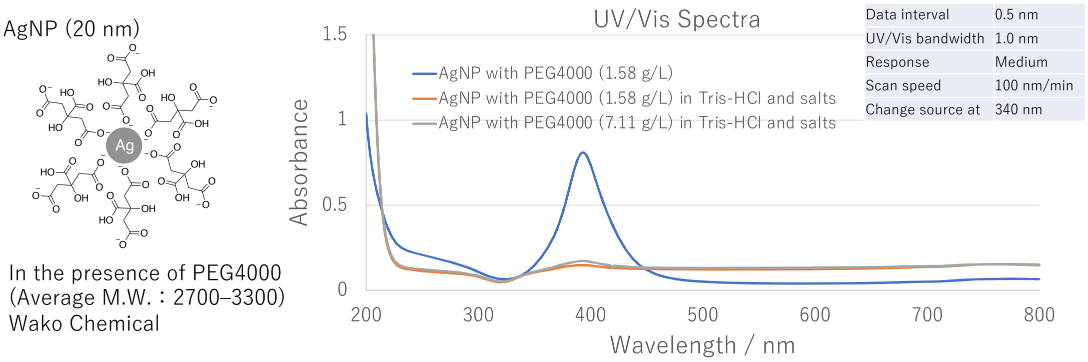

Figure S22. Absorption spectra to prove that the thiol group of surface-modifying PEG is important for interaction with silver nanoparticles, and that surface PEG modification improved the dispersibility of silver nanoparticles. In this experiment, silver nanoparticles (particle size 20 nm) were used in the presence of PEG4000 (average molecular weight 2700-3300, Fujifilm-Wako Pure Chemical Industries, without thiol function), which is considered not to directly interact with silver nanoparticles. The blue spectrum in the **Figure S22** shows the absorption behavior of a silver nanoparticle dispersion in which 1.58 g/L of PEG4000 was added. A spectrum with maximum absorption at 396 nm was observed, similar to the commercially available silver nanoparticle dispersion itself. However, when Tris-HCl buffer (pH 8.3) and an aqueous solution of potassium chloride and magnesium chloride were added as salts to this dispersion, the absorption near 396 nm almost disappeared, as shown by the gray and red spectra. It was shown that PEG4000, which does not bind the surface of nanoparticles, cannot protect the surface of silver nanoparticles meaning doesn't have an improved ability for the dispersibility of nanoparticles. This suggests that surface modification with thiolated PEG is essential for improving and maintaining the activity of silver nanoparticles.

9. Characterization of Silver Nanoparticles

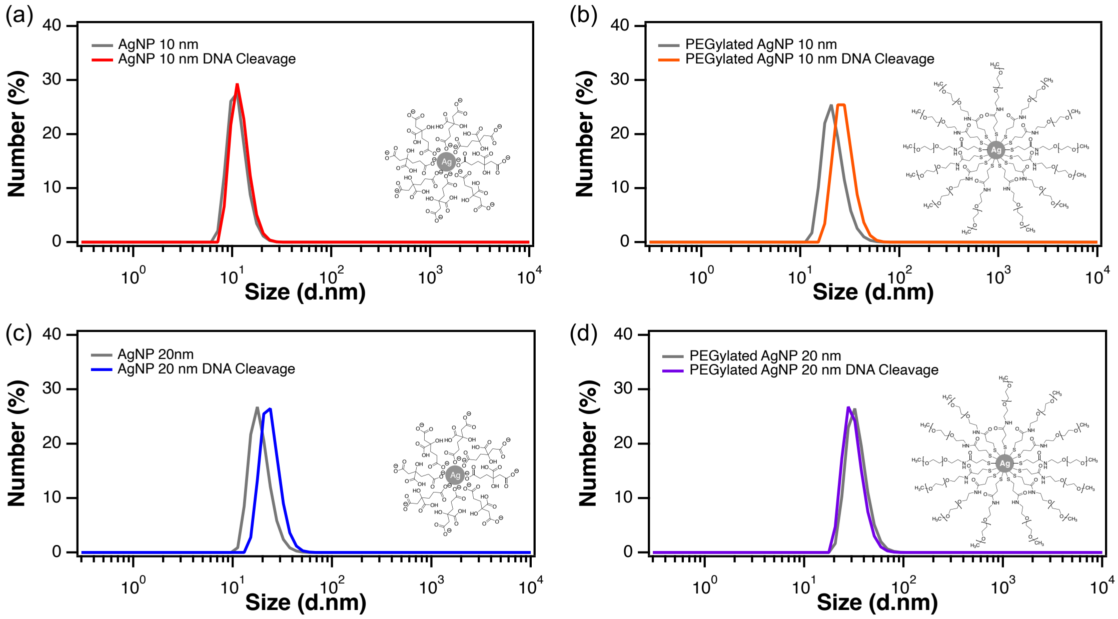

Figure S23. Nanoparticle sizes determined by DLS: (a) AgNPs (10 nm) before and after DNA strand cleavage; (b) PEGylated AgNPs (10 nm) before and after DNA strand cleavage; (c) AgNPs (20 nm) before and after DNA strand cleavage; (d) PEGylated AgNPs (20 nm) before and after DNA strand cleavage.

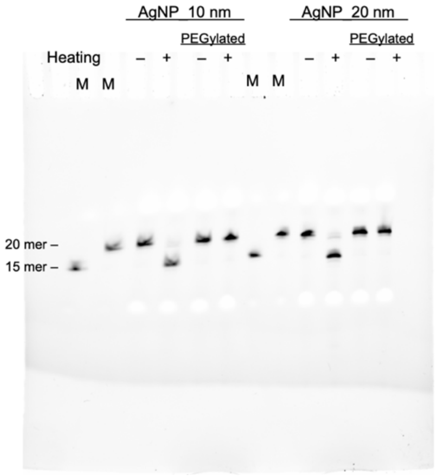

Figure S24. 15% dPAGE analysis to confirm the DNA strand cleavage occurred for DLS analysis of AgNP after DNA strand cleavage (mini-gel, 15% dPAGE, 30 mA, 20 min). A 20-nt 3'-FAM-labeled 3'-phosphorothiolate DNA (**3'S-DNA1**: 5'-TAAC**Ts**CACATTAATTGCGTT-FAM-3', where **Ts** denotes a 3′ -phosphorothiolate) was added to the AgNP dispersions and incubated at 95 ° C for 1 h (AgNP, 10 nm), 95 ° C for 2 h (AgNP, 20 nm), 50 ° C for 1 h (PEGylated AgNP, 10 nm), or 50 ° C for 2 h (PEGylated AgNP, 20 nm) to induce DNA cleavage. The resulting reaction solutions were then subjected to DLS measurements.

(a)

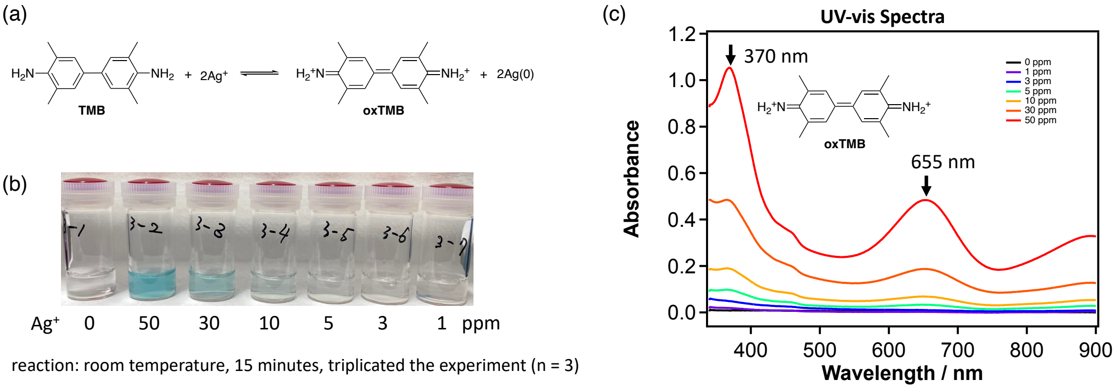

(d)

Ag^+ (ppm)	Abs. (655 nm)	SD Abs_{655}
0	0.0030	0.0014
1	0.0023	0.0026
3	0.0100	0.0059
5	0.0330	0.0065
10	0.0680	0.0102
30	0.1870	0.0182
50	0.4833	0.0548

n = 3

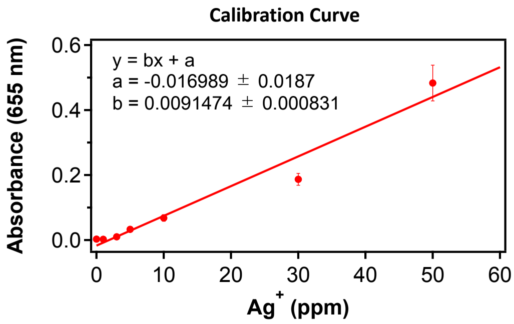

Figure S25. Preparation of a calibration curve for quantifying residual silver ions in AgNP dispersions. The experiment was conducted according to the method reported by González-Fuenzalida et al. (2013) (6). (a) Oxidation of 3,3',5,5'-tetramethylbenzidine (TMB) by silver ions to produce oxTMB. (b) Photograph of reaction mixtures containing 667 μM TMB and 0–467 μM silver nitrate (0–50 ppm Ag^+) in 133 mM NaOAc–AcOH buffer (pH 4). (c) UV–vis spectra of the mixtures shown in (b). Each experiment was performed in triplicate (n = 3). Data are presented as mean values (n = 3). (d) Table showing the mean absorbance at 655 nm and calculated standard deviation (S.D.) of oxTMB at various silver ion concentrations. A calibration curve was generated by linear fitting of these data. Error bars indicate variability among three independent experiments.

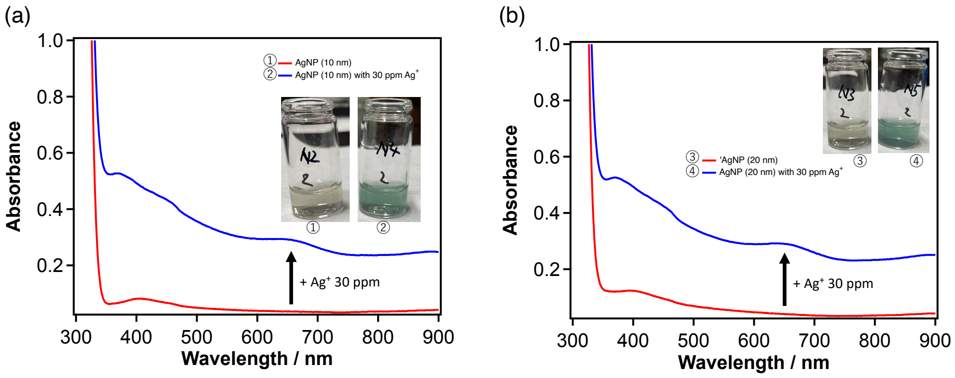

(c)

	Abs$_{655}$	Sample Solution Calcd. [Ag^{+}]	AgNP dispersion stock [Ag^{+}] (ppm)
AgNP (10 nm)	0.036	5.79 ppm	11.6 ppm
AgNP (20 nm)	0.040	6.23 ppm	12.5 ppm
AgNP (10 nm) with 30 ppm Ag^{+}	0.291	33.7 ppm	
AgNP (10 nm) with 30 ppm Ag^{+}	0.290	33.7 ppm	

(d) UV measurement sample solution

	AgNP
10 mM TMB in ethanol	100
1 M NaOAc/AcOH buffer (pH 4)	200
50 mM $AgNO_3$ aq.	-
AgNP dispersion (10 or 20 nm)	750
miliQ water	450
	1500 μL

Figure S26. Quantification of silver ions in AgNP dispersions based on oxTMB absorbance. (a) UV–vis spectra of AgNP dispersions (10 nm), (b) UV–vis spectra of AgNP dispersions (20 nm). The addition of 30 ppm Ag^{+} increased the absorbance, indicating that the absorption is sensitive to silver ions and enabling their quantification in AgNP suspensions. (c) Table showing absorbance at 655 nm derived from oxTMB and calculated silver ion concentrations in sample solutions and AgNP stock dispersions using the calibration curve (y = 0.0091474x – 0.016989). (d) Composition of UV measurement samples containing AgNPs.

10. Silver Nanoparticle Mediated Double Strand DNA Cleavage to Produce Overhang Structure

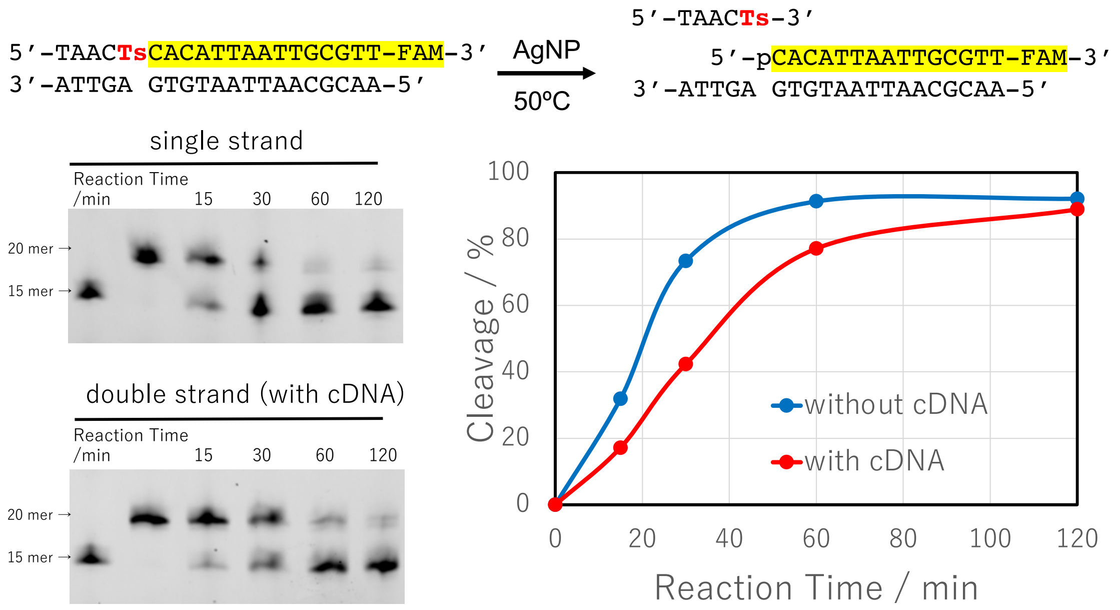

Figure S27. Preparation of 3' overhang sticky end by silver nanoparticle treatment of short double-stranded DNA. Cleavage of a 20-mer duplex DNA with a 3'-phosphorothiolate bond was performed using 1 nm PEGylated silver nanoparticle. The 3'-phosphorothiolate bond is positioned between the 5th and 6th bases from the 5' end, designed to produce a sticky end with a 5-base overhang upon cleavage. The reaction was performed at 50 ºC for 15, 30, and 60 minutes, and each reaction solution was analyzed by denaturing gel electrophoresis, and the cleavage efficiency at each reaction time was calculated from gel band intensity analysis using FAM-derived fluorescence detection. The cleavage efficiency was compared with and without complementary strands, and the cleavage efficiency at each reaction time was plotted on the vertical axis and the reaction time on the horizontal axis. As a result, in the presence of the complementary strand, although the cleavage activity was slightly lower than that of the case without the complementary strand, the cleavage activity was approximately the same 2 hours after the start of the reaction, and more than 90% of the DNA was cleaved. This revealed that DNA strand cleavage using PEGylated silver nanoparticles can be applied to the preparation of sticky-end DNA.

11. Application of 3'*S*-DNA for PCR

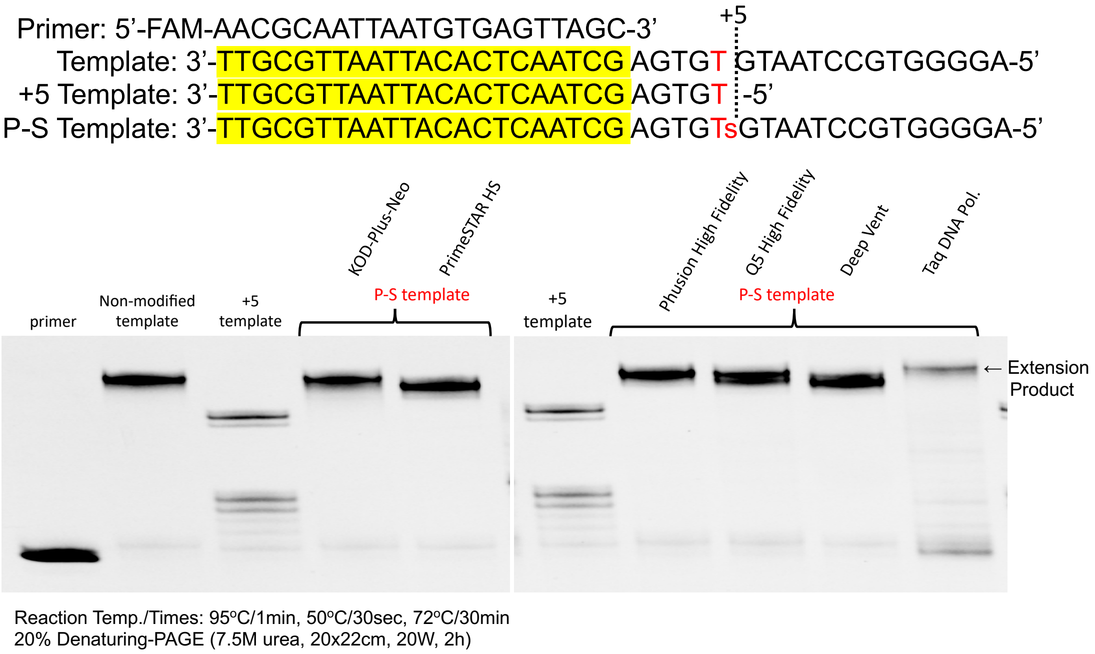

Figure S28. dPAGE analysis of PCR products when 3'-phosphorothiolate linkage was introduced into a DNA template. 22-mer 5'-FAM labeled DNA primer (5'-FAM-AACGCAATTAATGTGAGTTAGC) and template DNA (**3'*S*-DNA2**: 5'-AGGGGTGCCTAATG**Ts**GTGAGCTAACTCACATTAATTGCGTT-3') was annealed, and a polymerase extension reaction was performed using various polymerases (KOD-Plus-Neo, PrimeSTAR HS, Phusion High Fidelity, Q5 High Fidelity, Deep Vent, Taq DNA polymerase). When chain elongation stops at the 3'-phosphorothiolate modification site, a 4- or 5-base extension product is generated from the 22-mer primer. On the other hand, if the 3'-phosphorothiolate modification site is extended without any problems, full-length 41-mer DNA will be generated. The reaction solution was analyzed by 20% denaturing gel electrophoresis (containing 7.5 M urea, 20x22 cm, 20W, 2 hours), and the bands were visualized using a gel image analyzer (BioRad) using fluorescence detection derived from 5'-FAM. Experimental results showed that a band corresponding to the full-length 41-mer DNA was observed regardless of the polymerase used, with no elongation termination product detected at the 3'-phosphorothiolate site. This revealed that the 3'-thio-phosohorothiolate-modified DNA can be applicable for PCR.

12. Design and Synthesis of Long-Chain DNA by Ligation

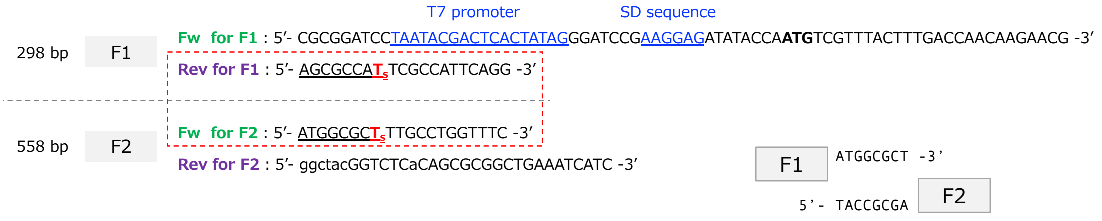

ATGTCGTTTACTTTGACCAACAAGAACGTGATTTTCGTTGCCGGTCTGGGAGGCATTGGTCTGGACACCAGCAAGGAGCTGCTCAAGCGCGATCCCGTCGTTTTA
CAACGTCGTGACTGGGAAAACCCTGGCGTTACCCAACTTAATCGCCTTGCAGCACATCCCCCTTTCGCCAGCTGGCGTAATAGCGAAGAGGCCCGCACCGATCGCCC
TTCCCAACAGTTGCGCAGCCTGAATGGCGAATGGCGCTTTGCCTGGTTTCCGGCACCAGAAGCGGTGCCGGAAAGCTGGCTGGAGTGCGATCTTCCTGAGGCCG
ATACTGTCGTCGTCCCCTCAAACTGGCAGATGCACGGTTACGATGCGCCCATCTACACCAACGTAACCTATCCCATTACGGTCAATCCGCCGTTTGTTCCCACGGAGA
ATCCGACGGGTTGTTACTCGCTCACATTTAATGTTGATGAAAGCTGGCTACAGGAAGGCCAGACGCGAATTATTTTTGATGGCGTTAACTCGGCGTTTCATCTGTGGT
GCAACGGGCGCTGGGTCGGTTACGGCCAGGACAGTCGTTTGCCGTCTGAATTTGACCTGAGCGCATTTTTACGCGCCGGAGAAAACCGCCTCGCGGTGATGGTGCT
GCGTTGGAGTGACGGCAGTTATCTGGAAGATCAGGATATGTGGCGGATGAGCGGCATTTTCCGTGACGTCTCGTTGCTGCATAAACCGACTACACAAATCAGCGATT
TCCATGTTGCCACTCGCTTTAATGATGATTTCAGCCGCGCTGtGAGACCgtagcc

Figure S29. The sequence design of a DNA ligation reaction utilizes the generation of sticky ends by DNA strand cleavage with silver nanoparticles. The total length of the ligated product DNA is 848 base pairs. To amplify the 298 bp F1 region, DNA primer (Fw for F1) and 3'*S*-DNA primer (**3'*S* -DNA3**: Rev for F1: 5'-AGCGCCA**Ts**TCGCCATTCAGG-3') were used for PCR. To amplify the 558 bp F2 region, 3'*S*-DNA primer (**3'*S*-DNA4**: Fw for F1: 5'-ATGGCGC**Ts**TTGCCTGGTTTC-3') and DNA primer (Rev for F2) were used for PCR. The design allows the preparation of sticky ends with 8 bases overhang from the 5'-terminus by amplifying DNA fragments of 298 base pairs and 558 base pairs by PCR and the following silver nanoparticle treatment. The red underlined part (ATGGCGCT) in the ligation product sequence corresponds to the sequence of the 8-base overhang, by treating the 298-base pair and 558-base pair PCR products with silver nanoparticles and ligating them with T4 DNA ligase, a full-length 848-base pair DNA can be synthesized.

Table S3. Primer Sequences for the BsaI Digestion Control Experiment Related to Figure 5.

name	Sequence (5'→3')
**Rev for F1**	GGCTACGGTCTCCGCCATTCGCCATTCAGGC
**Fw for F2**	GGCTACGGTCTCATGGCGCTTTGCCTGGTTTC

(a)

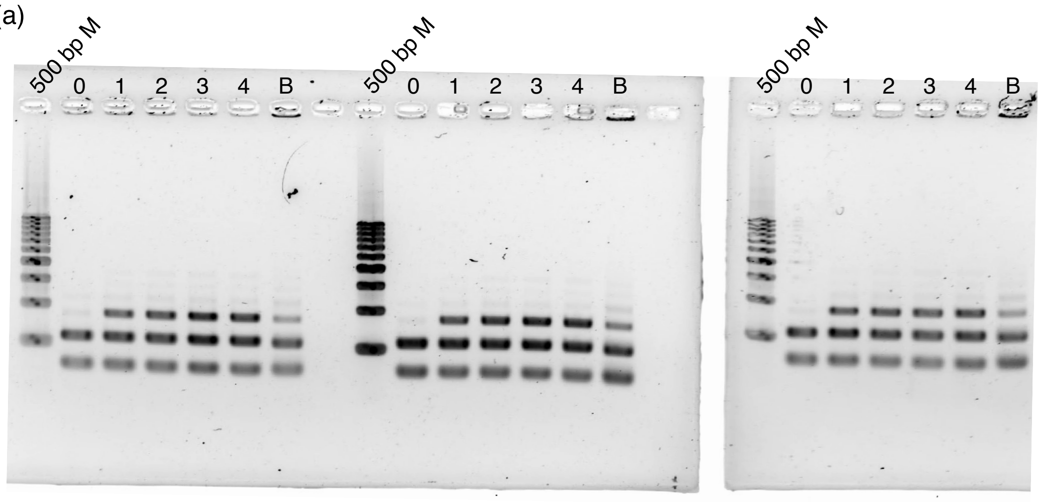

0-4：AgNP-treated samples reacted at 50 °C for various durations 0–4 hours（8 mer overhang）
B：BsaI-treated sample（4 mer overhang）

(b)

raw data	0h				
	n = 1	n = 2	n = 3	n = 4	
848	7184.52	13125.43	13773.13	13282.43	
557	21281.86	24388.38	30950.85	25173.00	
298	15820.07	21216.22	26661.03	22940.35	
background	6374.12	12745.37	13502.40	13247.19	
**-background**	**0h**				
	n = 1	n = 2	n = 3	n = 4	
848	810.40	380.06	270.73	35.25	
557	14907.74	11643.01	17448.45	11925.81	
298	9445.95	8470.85	13158.63	9693.16	
**/ length**	**0h**				
	n = 1	n = 2	n = 3	n = 4	
848	0.96	0.45	0.32	0.04	
557	26.76	20.90	31.33	21.41	
298	31.70	28.43	44.16	32.53	
bp	3.17%	1.78%	0.84%	0.15%	1.49%
					0.013
					0.93%
					0.008

raw data	1h				
	n = 1	n = 2	n = 3	n = 4	
848	10085.31	20679.91	22601.21	21228.23	
557	20060.93	25742.16	29849.10	26950.08	
298	15498.22	22759.40	25030.12	23397.18	
background	6255.84	13107.11	13390.92	13699.69	
**-background**	**1h**				
	n = 1	n = 2	n = 3	n = 4	
848	3829.48	7572.80	9210.29	7528.55	
557	13805.09	12635.05	16458.18	13250.40	
298	9242.38	9652.29	11639.20	9697.50	
**/ length**	**1h**				
	n = 1	n = 2	n = 3	n = 4	
848	4.52	8.93	10.86	8.88	
557	24.78	22.68	29.55	23.79	
298	31.01	32.39	39.06	32.54	
bp	13.93%	24.49%	24.05%	23.97%	21.61%
					0.051
					24.17%
					0.003

raw data	2h				
	n = 1	n = 2	n = 3	n = 4	
848	11442.68	22642.33	25034.65	22441.39	
557	20508.05	26291.20	29556.46	25721.97	
298	15357.49	23087.22	25281.03	22665.90	
background	6267.40	13374.14	13522.40	13741.94	
**-background**	**2h**				
	n = 1	n = 2	n = 3	n = 4	
848	5175.28	9268.19	11512.26	8699.45	
557	14240.66	12917.06	16034.06	11980.03	
298	9090.10	9713.08	11758.63	8923.96	
**/ length**	**2h**				
	n = 1	n = 2	n = 3	n = 4	
848	6.10	10.93	13.58	10.26	
557	25.57	23.19	28.79	21.51	
298	30.50	32.59	39.46	29.95	
bp	17.88%	28.15%	28.46%	28.51%	25.75%
					0.053
					28.37%
					0.002

raw data	3h				
	n = 1	n = 2	n = 3	n = 4	
848	11317.4	25061.9	25619.2	21831.6	
557	20094.8	28710.8	29666.9	24527.6	
298	14870.3	24596.8	24777.3	22059.8	
background	6333.3	13360.6	13314.7	13930.6	
**-background**	**3h**				
	n = 1	n = 2	n = 3	n = 4	
848	4984.13	11701.25	12304.48	7900.98	
557	13761.45	15350.13	16352.17	10596.94	
298	8536.99	11236.18	11462.54	8129.21	
**/ length**	**3h**				
	n = 1	n = 2	n = 3	n = 4	
848	5.88	13.80	14.51	9.32	
557	24.71	27.56	29.36	19.03	
298	28.65	37.71	38.46	27.28	
bp	18.05%	29.72%	29.97%	28.70%	26.61%
					0.057
					29.46%
					0.007

raw data	4h				
	n = 1	n = 2	n = 3	n = 4	
848	11475.65	24148.44	25121.27	23051.18	
557	19197.22	27734.09	28595.53	26024.19	
298	14660.44	23778.08	24690.93	23183.01	
background	6335.73	13614.05	13186.08	14024.29	
**-background**	**4h**				
	n = 1	n = 2	n = 3	n = 4	
848	5139.92	10534.39	11935.19	9026.89	
557	12861.49	14120.04	15409.45	11999.90	
298	8324.71	10164.03	11504.85	9158.72	
**/ length**	**4h**				
	n = 1	n = 2	n = 3	n = 4	
848	6.06	12.42	14.07	10.64	
557	23.09	25.35	27.67	21.54	
298	27.94	34.11	38.61	30.73	
bp	19.20%	29.47%	29.81%	28.94%	26.85%
					0.051
					29.41%
					0.004

raw data	BsaI				
	n = 1	n = 2	n = 3	n = 4	
848	12125.39	16680.76	18578.56	17018.99	
557	22964.05	24321.37	26948.54	24169.96	
298	16248.17	22305.22	27006.11	24501.61	
background	6401.49	13706.32	13243.28	13942.43	
**-background**	**BsaI**				
	n = 1	n = 2	n = 3	n = 4	
848	5723.91	2974.44	5335.28	3076.57	
557	16562.56	10615.05	13705.26	10227.53	
298	9846.68	8598.90	13762.83	10559.18	
**/ length**	**BsaI**				
	n = 1	n = 2	n = 3	n = 4	
848	6.75	3.51	6.29	3.63	
557	29.74	19.06	24.61	18.36	
298	33.04	28.86	46.18	35.43	
bp	17.70%	12.77%	15.09%	11.89%	14.36%
					0.026
					13.25%
					0.017

Figure S30. (a) Agarose gel analysis of the ligation reaction of DNA fragments by T4 DNA ligase. DNA fragments with sticky ends were prepared by PEGylated AgNP treatment for 0, 1, 2, 3, and 4 h or BsaI treatment. PCR by using 3'*S*-DNA primers was conducted with 0.50 μM 3'*S*-DNA forward primer, 0.50 μM 3'*S*-DNA reverse primer, 0.50 ng/μL template DNA, 0.20 mM dNTP mixture, 1.5 mM $MgSO_4$, 1xPCR Buffer, and KOD-Plus-Neo (1 U). After PCR, the amplified 7.5 nM 3'*S*-DNA was cleaved by 0.017 mg/mL PEGylated AgNPs with 10 nm particle size at 50 °C for 1–4 h to prepare sticky ends. Ligation of DNA fragments with sticky ends was carried out with 3.0 nM DNA fragment1, 3.0 nM DNA fragment2, 1xReaction buffer, and T4 DNA ligase (900 U) at 25 ºC for 3 h. (b) Raw data for Figure 5(d). The same experiment was repeated four times and calculated the standard deviation.

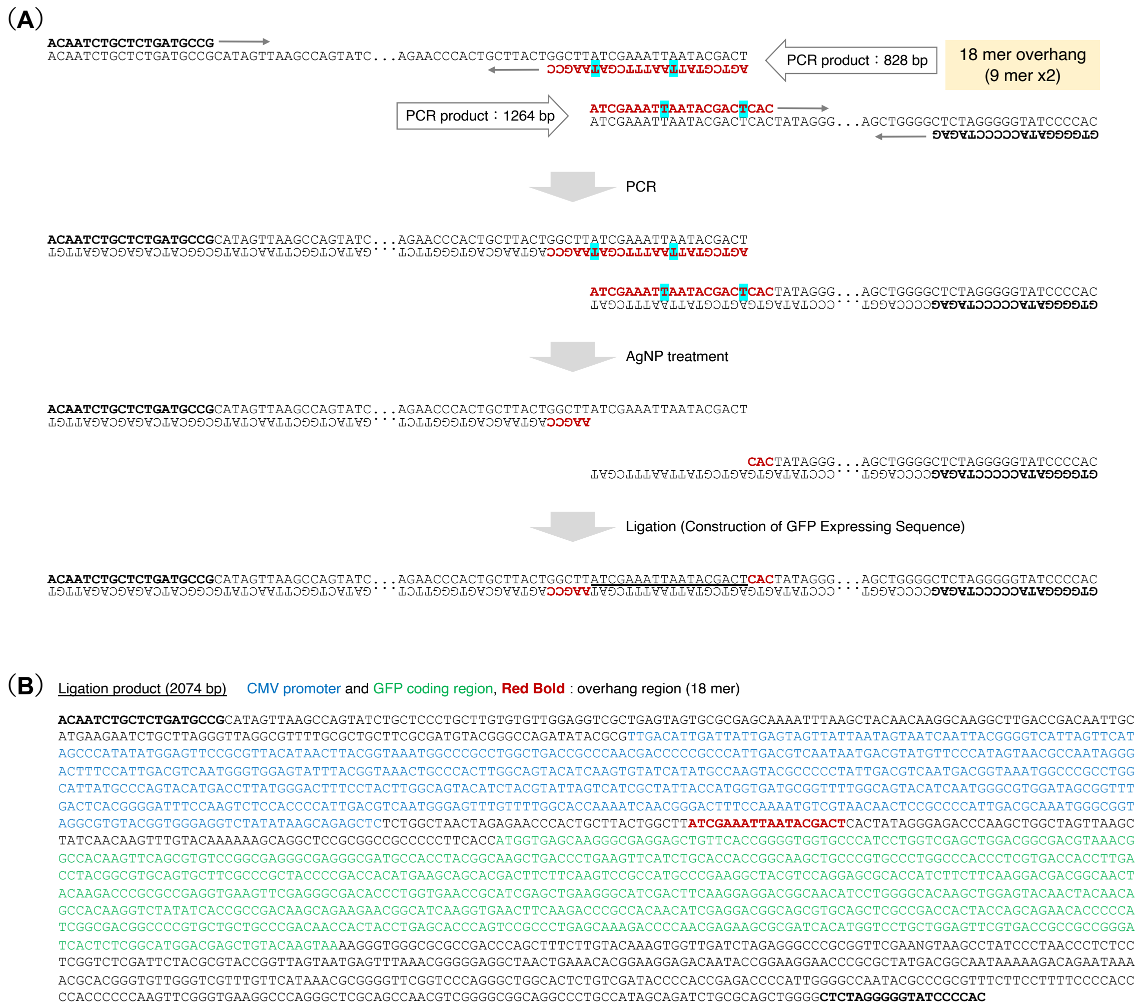

Figure S31. The sequence design of a DNA ligation reaction to synthesize DNA, consisting of a CMV promoter and GFP coding sequence, utilizes the generation of sticky ends through DNA strand cleavage with PEGylated silver nanoparticle (10 nm) at 50 ºC for 4 hours and and dissociation of cleaved fragments by heating at 90 ºC. (A) To amplify the 828 bp CMV coding region, DNA primer (CMV-Fw) and 3'*S*-DNA primer (**3'*S*-DNA5**: CMV-Rev-Ts18: 5'-AGTCGTAT**Ts**AATTTCGA**Ts**AAGCC-3') were used for PCR. To amplify the 1,264 bp GFP region, 3'*S*-DNA primer (**3'*S*-DNA7**: GFP-fw-Ts18: 5'- ATCGAAAT**Ts**AATACGAC**Ts**CAC-3') and DNA primer (GFP-Rev) were used for PCR. The design allows the preparation of sticky ends with 18 bases overhang from the 5'-terminus by amplifying DNA fragments of 828 base pairs and 1,264 base pairs by PCR and the following PEGylated silver nanoparticle (10 nm) treatment. The red capitals (ATCGAAATTAATACGACT) in the ligation product sequence corresponds to the sequence of the 18-base overhang, by treating the 828-base pair and 1,264-base pair PCR products with silver nanoparticles and ligating them with T4 DNA ligase, a full-length 2,074-base pair DNA can be synthesized. (B) The total length of the ligated product DNA is 2,074 base pairs.

(A)

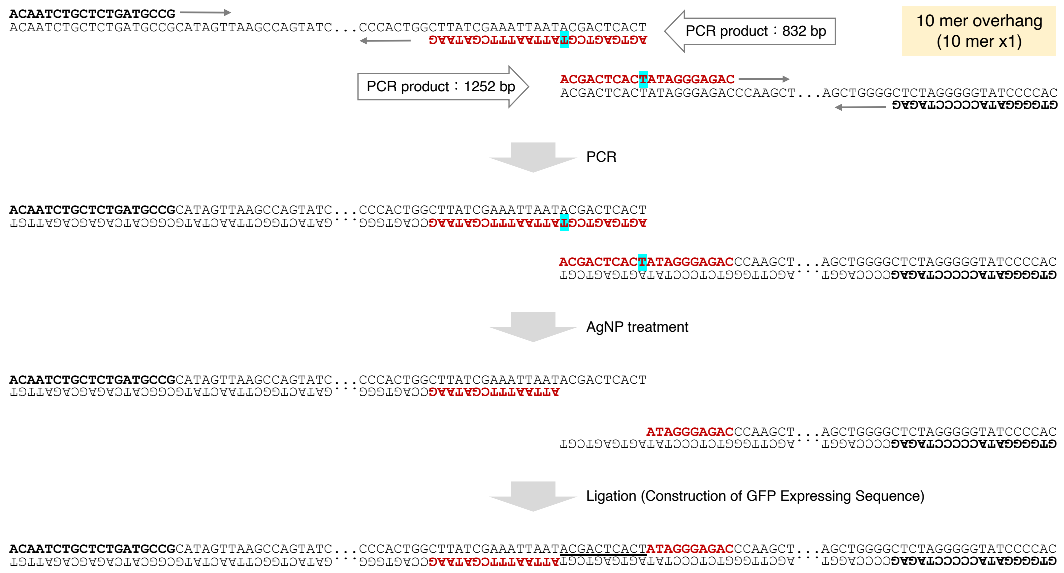

(B)

Ligation product (2074 bp) CMV promoter and GFP coding region, **Red Bold** : overhang region (10 mer)

```
ACAATCTGCTCTGATGCCGCATAGTTAAGCCAGTATCTGCTCCCTGCTTGTGTGTTGGAGGTCGCTGAGTAGTGCGCGAGCAAAATTTAAGCTACAACAAGGCAAGGCTTGACCGACAATTGC
ATGAAGAATCTGCTTAGGGTTAGGCGTTTTGCGCTGCTTCGCGATGTACGGGCCAGATATACGCGTTGACATTGATTATTGAGTAGTTATTAATAGTAATCAATTACGGGGTCATTAGTTCAT
AGCCCATATATGGAGTTCCGCGTTACATAACTTACGGTAAATGGCCCGCCTGGCTGACCGCCCAACGACCCCCGCCCATTGACGTCAATAATGACGTATGTTCCCATAGTAACGCCAATAGGG
ACTTTCCATTGACGTCAATGGGTGGAGTATTTACGGTAAACTGCCCACTTGGCAGTACATCAAGTGTATCATATGCCAAGTACGCCCCCTATTGACGTCAATGACGGTAAATGGCCCGCCTGG
CATTATGCCCAGTACATGACCTTATGGGACTTTCCTACTTGGCAGTACATCTACGTATTAGTCATCGCTATTACCATGGTGATGCGGTTTTGGCAGTACATCAATGGGCGTGGATAGCGGTTT
GACTCACGGGGATTTCCAAGTCTCCACCCCATTGACGTCAATGGGAGTTTGTTTTGGCACCAAAATCAACGGGACTTTCCAAAATGTCGTAACAACTCCGCCCCATTGACGCAAATGGGCGGT
AGGCGTGTACGGTGGGAGGTCTATATAAGCAGAGCTCTCTGGCTAACTAGAGAACCCACTGCTTACTGGCTTATCGAAATTAATACGACTCACTATAGGGAGACCCAAGCTGGCTAGTTAAGC
TATCAACAAGTTTGTACAAAAAAGCAGGCTCCGCGGCCGCCCCCTTCACCATGGTGAGCAAGGGCGAGGAGCTGTTCACCGGGGTGGTGCCCATCCTGGTCGAGCTGGACGGCGACGTAAACG
GCCACAAGTTCAGCGTGTCCGGCGAGGGCGAGGGCGATGCCACCTACGGCAAGCTGACCCTGAAGTTCATCTGCACCACCGGCAAGCTGCCCGTGCCCTGGCCCACCCTCGTGACCACCTTGA
CCTACGGCGTGCAGTGCTTCGCCCGCTACCCCGACCACATGAAGCAGCACGACTTCTTCAAGTCCGCCATGCCCGAAGGCTACGTCCAGGAGCGCACCATCTTCTTCAAGGACGACGGCAACT
ACAAGACCCGCGCCGAGGTGAAGTTCGAGGGCGACACCCTGGTGAACCGCATCGAGCTGAAGGGCATCGACTTCAAGGAGGACGGCAACATCCTGGGGCACAAGCTGGAGTACAACTACAACA
GCCACAAGGTCTATATCACCGCCGACAAGCAGAAGAACGGCATCAAGGTGAACTTCAAGACCCGCCACAACATCGAGGACGGCAGCGTGCAGCTCGCCGACCACTACCAGCAGAACACCCCCA
TCGGCGACGGCCCCGTGCTGCTGCCCGACAACCACTACCTGAGCACCCAGTCCGCCCTGAGCAAAGACCCCAACGAGAAGCGCGATCACATGGTCCTGCTGGAGTTCGTGACCGCCGCCGGGA
TCACTCTCGGCATGGACGAGCTGTACAAGTAAAAGGGTGGGCGCGCCGACCCAGCTTTCTTGTACAAAGTGGTTGATCTAGAGGGCCCGCGGTTCGAANGTAAGCCTATCCCTAACCCTCTCC
TCGGTCTCGATTCTACGCGTACCGGTTAGTAATGAGTTTAAACGGGGGAGGCTAACTGAAACACGGAAGGAGACAATACCGGAAGGAACCCGCGCTATGACGGCAATAAAAAGACAGAATAAA
ACGCACGGGTGTTGGGTCGTTTGTTCATAAACGCGGGGTTCGGTCCCAGGGCTGGCACTCTGTCGATACCCCACCGAGACCCCATTGGGGCCAATACGCCCGCGTTTCTTCCTTTTCCCCACC
CCACCCCCCAAGTTCGGGTGAAGGCCCAGGGCTCGCAGCCAACGTCGGGGCGGCAGGCCCTGCCATAGCAGATCTGCGCAGCTGGGGCTCTAGGGGGTATCCCCAC
```

Figure S32. The sequence design of a DNA ligation reaction to synthesize DNA, consisting of a CMV promoter and GFP coding sequence, utilizes the generation of sticky ends through DNA strand cleavage with PEGylated silver nanoparticle (10 nm) at 50 ºC for 4 hours and and dissociation of cleaved fragments by heating at 90 ºC. (A) To amplify the 832 bp CMV coding region, DNA primer (CMV-Fw) and 3'*S*-DNA primer (**3'*S*-DNA6**: CMV-Rev-Ts10: 5'-AGTGAGTCG**Ts**ATTAATTTCGATAAG-3') were used for PCR. To amplify the 1,252 bp GFP region, 3'*S*-DNA primer (**3'*S*-DNA8**: GFP-fw-Ts10: 5'-ACGACTCAC**Ts**ATAGGGAGAC-3') and DNA primer (GFP-Rev) were used for PCR. The design allows the preparation of sticky ends with 10 bases overhang from the 5'-terminus by amplifying DNA fragments of 832 base pairs and 1,252 base pairs by PCR and the following PEGylated silver nanoparticle (10 nm) treatment. The red capitals (ACGACTCACT) in the ligation product sequence corresponds to the sequence of the 10-base overhang, by treating the 832-base pair and 1,252-base pair PCR products with silver nanoparticles and ligating them with T4 DNA ligase, a full-length 2,074-base pair DNA can be synthesized. (B) The total length of the ligated product DNA is 2,074 base pairs.

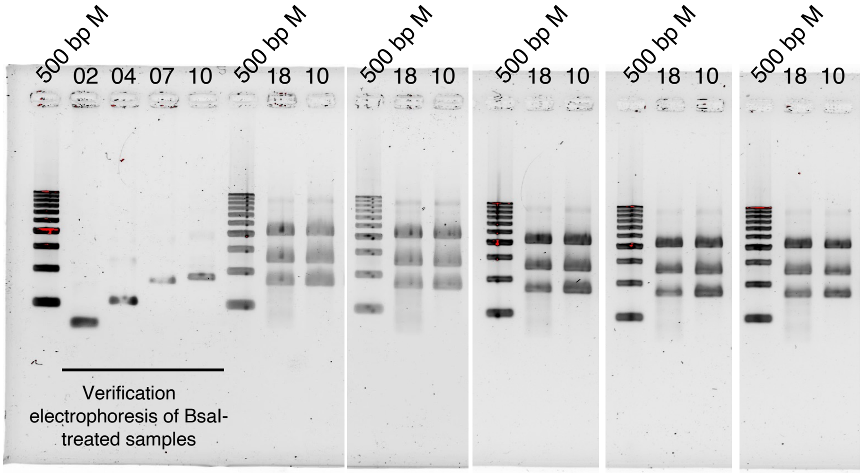

18, 10: Ligation samples of 18- and 10-mer overhang fragments obtained by AgNP treatment

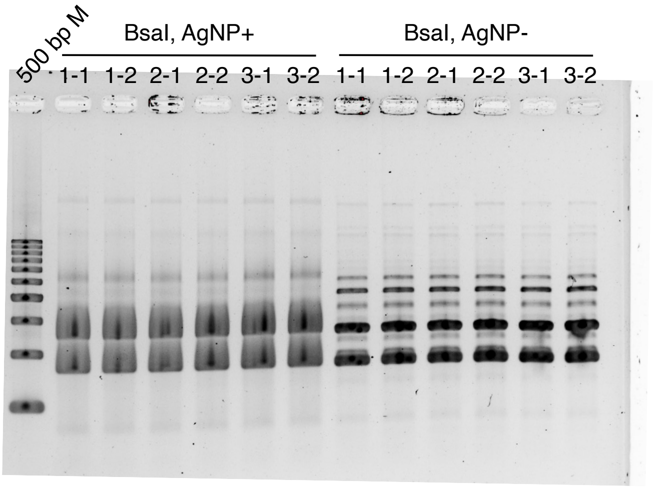

Ligation samples of BsaI-treated fragments (+: with AgNP treatment process, -: without)

Figure S33. Agarose gel analysis of the ligation reaction of the DNA fragments with 18, 10, and 4 (BsaI-treated) bases overhanging sticky ends. The same experiment was repeated six or seven times and calculated the standard deviation.

raw data	18 mer, AgNP+						
	n = 1	n = 2	n = 3	n = 4	n = 5	n = 6	
2074	21011.18	16109.07	16487.99	21139.85	19580.60	19794.53	
1264	18971.35	15547.75	15579.05	18246.24	17004.41	16327.69	
828	14204.93	14545.02	14254.08	15780.49	14208.92	14352.45	
background	11477.92	9392.18	9843.09	8367.74	8843.76	7947.53	
	8467.78	10493.88	11293.17	10061.70	8978.32	9299.55	
	5457.64	10667.39	11151.81	10492.96	9712.49	8623.99	

-background	18 mer, AgNP+						
	n = 1	n = 2	n = 3	n = 4	n = 5	n = 6	
2074	9533.26	6716.88	6644.90	12772.11	10736.84	11847.00	
1264	10503.56	5053.87	4285.88	8184.53	8026.08	7028.14	
828	8747.28	3877.63	3102.27	5287.53	4496.42	5728.46	

/ length	18 mer, AgNP+						
	n = 1	n = 2	n = 3	n = 4	n = 5	n = 6	
2074	4.60	3.24	3.20	6.16	5.18	5.71	
1264	8.31	4.00	3.39	6.48	6.35	5.56	
828	10.56	4.68	3.75	6.39	5.43	6.92	
bp	32.75%	42.73%	47.31%	48.92%	46.78%	47.79%	44.38%
							0.061
							46.71%
							0.024

raw data	10 mer, AgNP+						
	n = 1	n = 2	n = 3	n = 4	n = 5	n = 6	
2074	18283.19	15073.28	16410.67	22288.73	20347.01	18142.53	
1252	18299.51	15407.77	15288.21	20307.31	18588.71	15914.55	
832	16304.37	15261.38	14567.24	19888.78	19100.83	15913.82	
background	8415.34	10673.09	10720.73	10535.39	9214.59	7917.96	
	7644.87	11852.36	12317.17	12079.00	10449.88	9512.49	
	6874.40	12059.06	11342.23	10201.93	9976.78	8868.27	

-background	10 mer, AgNP+						
	n = 1	n = 2	n = 3	n = 4	n = 5	n = 6	
2074	9867.86	4400.19	5689.95	11753.34	11132.42	10224.57	
1252	10654.64	3555.42	2971.04	8228.31	8138.83	6402.06	
832	9429.97	3202.32	3225.00	9686.86	9124.05	7045.55	

/ length	10 mer, AgNP+						
	n = 1	n = 2	n = 3	n = 4	n = 5	n = 6	
2074	4.76	2.12	2.74	5.67	5.37	4.93	
1252	8.51	2.84	2.37	6.57	6.50	5.11	
832	11.33	3.85	3.88	11.64	10.97	8.47	
bp	32.41%	38.81%	46.75%	38.36%	38.07%	42.06%	39.41%
							0.048
							40.81%
							0.037

raw data	Bsal, AgNP+							
	n = 1	n = 2	n = 3	n = 4	n = 5	n = 6	n = 7	
2074	8070.34	2236.45	2160.84	2128.60	2131.76	2222.56	2212.10	
1261	22079.76	3702.13	3792.04	3831.05	3909.99	4039.25	4286.13	
824	19652.92	3495.89	3665.57	3573.01	3869.17	3920.66	4115.11	
background	6568.39	1861.91	1780.51	1738.27	1697.51	1780.65	1800.73	
	5978.32	1843.15	1800.04	1742.65	1710.46	1762.51	1776.59	
	5388.26	1824.39	1819.57	1747.04	1723.41	1744.37	1752.45	

-background	Bsal, AgNP+							
	n = 1	n = 2	n = 3	n = 4	n = 5	n = 6	n = 7	
2074	1501.95	374.53	380.32	390.34	434.24	441.91	411.37	
1261	16101.44	1858.98	1991.99	2088.39	2199.53	2276.74	2509.54	
824	14264.66	1671.50	1845.99	1825.97	2145.76	2176.29	2362.66	

/ length	Bsal, AgNP+							
	n = 1	n = 2	n = 3	n = 4	n = 5	n = 6	n = 7	
2074	0.72	0.18	0.18	0.19	0.21	0.21	0.20	
1261	12.77	1.47	1.58	1.66	1.74	1.81	1.99	
824	17.31	2.03	2.24	2.22	2.60	2.64	2.87	
bp	4.59%	9.35%	8.76%	8.86%	8.78%	8.75%	7.55%	8.09%
								0.016
								8.67%
								0.006

raw data	Bsal, AgNP-							
	n = 1	n = 2	n = 3	n = 4	n = 5	n = 6	n = 7	
2074	8343.67	2635.17	2726.31	2700.51	2715.45	2539.83	2613.48	
1261	18452.19	4358.49	4723.01	4457.50	4702.91	4532.95	4855.32	
824	17707.25	4352.12	4690.57	4618.25	4781.38	4749.26	4666.29	
background	5496.95	1581.62	1547.37	1503.80	1445.65	1337.12	1295.47	
	5251.80	1599.26	1580.88	1534.81	1492.93	1420.20	1328.92	
	5006.65	1616.90	1614.38	1565.81	1540.21	1503.29	1362.37	

-background	Bsal, AgNP-							
	n = 1	n = 2	n = 3	n = 4	n = 5	n = 6	n = 7	
2074	2846.72	1053.55	1178.94	1196.71	1269.80	1202.71	1318.01	
1261	13200.39	2759.23	3142.14	2922.69	3209.98	3112.75	3526.40	
824	12700.60	2735.22	3076.19	3052.44	3241.16	3245.97	3303.91	

/ length	Bsal, AgNP-							
	n = 1	n = 2	n = 3	n = 4	n = 5	n = 6	n = 7	
2074	1.37	0.51	0.57	0.58	0.61	0.58	0.64	
1261	10.47	2.19	2.49	2.32	2.55	2.47	2.80	
824	15.41	3.32	3.73	3.70	3.93	3.94	4.01	
bp	9.59%	15.57%	15.44%	16.08%	15.90%	15.33%	15.74%	14.81%
								0.023
								15.68%
								0.003

Figure S34. Raw data for Figure 6(b). The same experiment was repeated six or seven times and calculated the standard deviation.

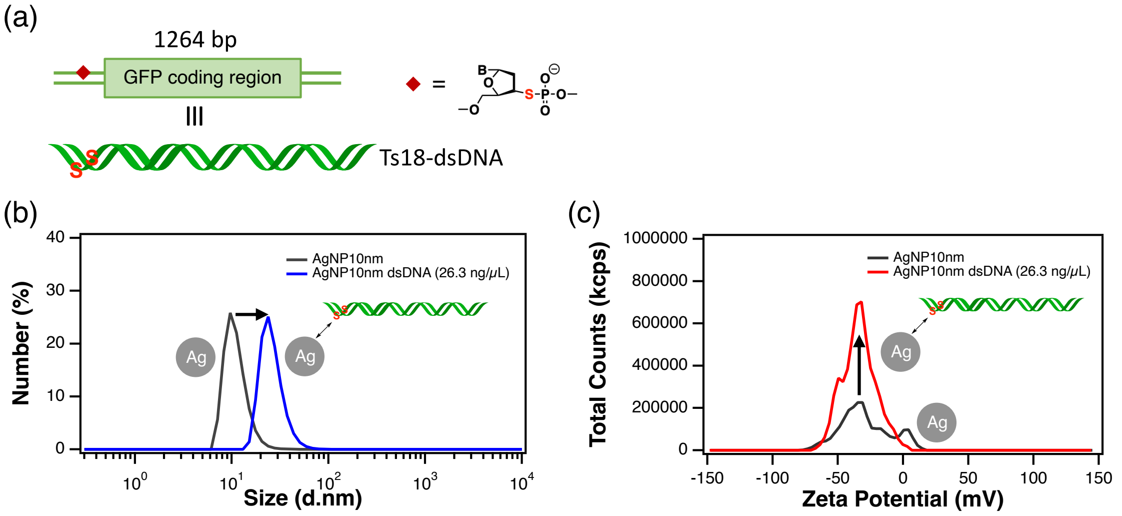

(d)

sample	Zeta Potential (mV)	Particle Size (nm)	PDI
AgNP10nm	–29.37	11.15	0.5432
AgNP10nm dsDNA (26.3 ng/μL)	–33.69	26.41	0.4688

Figure S35. Dynamic light scattering (DLS) was performed to analyze AgNP suspension in the presence of long dsRNA (1,264-bp). Measurements were taken immediately after mixing the AgNP suspension with the dsDNA solution at room temperature. It should be noted that DNA strand cleavage did not occur in the absence of heating. The objective was to assess differences in AgNP properties in the presence of long dsDNA. (a) Information of dsDNA. A 1,264 bp GFP coding dsDNA (PCR product) was used in this experiment. The red dot and "S" indicate 3'-phosphorothiolate nucleotide/linkage. (b) Particle size of AgNP (10 nm, 0.017 mg/mL) in the presence or absence of long dsDNA (26.3 ng/μL). (c) ζ-potential of AgNP (10 nm, 0.017 mg/mL) in the presence or absence of long dsDNA (26.3 ng/μL). (d) Summary table of ζ-potential, particle size, and polydispersity index (PDI) of the AgNP suspension.

13. References

1. Fulmer GR, Miller AJM, Sherden NH, Gottlieb HE, Nudelman A, Stoltz BM, Bercaw JE and Goldberg KI. NMR Chemical Shifts of Trace Impurities: Common Laboratory Solvents, Organics, and Gases in Deuterated Solvents Relevant to the Organometallic Chemist. *Organometallics* 2010;**29**:2176–79. https://doi.org/10.1021/om100106e
2. Sabbagh G, Fettes KJ, Gosain R, O'Neil IA and Cosstick R. Synthesis of phosphorothioamidites derived from 3'-thio-3'-deoxythymidine and 3'-thio-2',3'-dideoxycytidine and the automated synthesis of oligodeoxynucleotides containing a 3'-S-phosphorothiolate linkage. *Nucleic Acids Res* 2004;**32**:495–501. https://doi.org/10.1093/nar/gkh189
3. Gaynor JW, Bentley J and Cosstick R. Synthesis of the 3′-thio-nucleosides and subsequent automated synthesis of oligodeoxynucleotides containing a 3′-S-phosphorothiolate linkage. *Nat Protoc* 2007;**2**:3122–35. https://doi.org/10.1038/nprot.2007.451
4. Noble P and Tarbell DS. Thiobenzoic Acid - Benzoic Acid, Thio. *Org Synth* 1952;**32**:101-04. https://doi.org/10.15227/orgsyn.032.0101
5. Duschmale J, Hansen HF, Duschmale M, Koller E, Albaek N, Moller MR, Jensen K, Koch T, Wengel J and Bleicher K. In vitro and in vivo properties of therapeutic oligonucleotides containing non-chiral 3' and 5' thiophosphate linkages. *Nucleic Acids Res* 2020;**48**:63–74. https://doi.org/10.1093/nar/gkz1099
6. González-Fuenzalida RA, Moliner-Martínez Y, González-Béjar M, Molins-Legua C, Verdú-Andres J, Pérez-Prieto J and Campins-Falcó P. In Situ Colorimetric Quantification of Silver Cations in the Presence of Silver Nanoparticles. *Anal Chem* 2013;**85**:10013–16. https://doi.org/10.1021/ac402822d
7. Cava MP and Levinson MI. Thionation Reactions of Lawesson Reagents. *Tetrahedron* 1985;**41**:5061–87. https://doi.org/10.1016/S0040-4020(01)96753-5
8. Cosstick R and Vyle JS. Synthesis and Properties of Dithymidine Phosphate Analogs Containing 3'-Thiothymidine. *Nucleic Acids Res* 1990;**18**:829–35. https://doi.org/10.1093/nar/18.4.829
9. Vyle JS, Connolly BA, Kemp D and Cosstick R. Sequence-Specific and Strand-Specific Cleavage in Oligodeoxyribonucleotides and DNA Containing 3'-Thiothymidine. *Biochemistry* 1992;**31**:3012–18. https://doi.org/10.1021/bi00126a024
10. Jensen RH and Davidson N. Spectrophotometric Potentiometric and Density Gradient Ultracentrifugation Studies of Binding of Silver Ion by DNA. *Biopolymers* 1966;**4**:17–32. https://doi.org/10.1002/bip.1966.360040104
11. Axson JL, Stark DI, Bondy AL, Capracotta SS, Maynard AD, Philbert MA, Bergin IL and Ault AP. Rapid Kinetics of Size and pH-Dependent Dissolution and Aggregation of Silver Nanoparticles in Simulated Gastric Fluid. *J Phys Chem C* 2015;**119**:20632–41. https://doi.org/10.1021/acs.jpcc.5b03634
12. Bell RA and Kramer JR. Structural chemistry and geochemistry of silver-sulfur compounds: Critical review. *Environ Toxicol Chem* 1999;**18**:9–22. https://doi.org/10.1002/etc.5620180103
13. Simáková P, Gautier J, Procházka M, Hervé-Aubert K and Chourpa I. Polyethylene-glycol-Stabilized Ag Nanoparticles for Surface-Enhanced Raman Scattering Spectroscopy: Ag Surface Accessibility Studied Using Metalation of Free-Base Porphyrins. *J Phys Chem C* 2014;**118**:7690–97. https://doi.org/10.1021/jp5005709
